# Sentinel2GlobalLULC: A Sentinel-2 RGB image tile dataset for global land use/cover mapping with deep learning

**DOI:** 10.1038/s41597-022-01775-8

**Published:** 2022-11-09

**Authors:** Yassir Benhammou, Domingo Alcaraz-Segura, Emilio Guirado, Rohaifa Khaldi, Boujemâa Achchab, Francisco Herrera, Siham Tabik

**Affiliations:** 1 Department of Computer Science and Artificial Intelligence, Andalusian Research Institute in Data Science and Computational Intelligence, DaSCI, University of Granada, 18071 Granada, Spain; 2 Systems Analysis and Modeling for Decision Support Laboratory, Higher National School of Applied Sciences of Berrechid, Hassan 1st University, Berrechid, 218 Morocco; 3 LifeWatch-ERIC ICT Core, 41071 Seville, Spain; 4 Department of Botany, Faculty of Science, University of Granada, 18071 Granada, Spain; 5 iEcolab, Inter-University Institute for Earth System Research, University of Granada, 18006 Granada, Spain; 6 Andalusian Center for Assessment and Monitoring of Global Change (CAESCG), University of Almería, 04120 Almería, Spain; 7 Multidisciplinary Institute for Environment Studies “Ramon Margalef”, University of Alicante, San Vicente del Raspeig, 03690 Alicante, Spain

**Keywords:** Environmental impact, Macroecology, Conservation biology, Climate and Earth system modelling, Ecosystem services

## Abstract

Land-Use and Land-Cover (LULC) mapping is relevant for many applications, from Earth system and climate modelling to territorial and urban planning. Global LULC products are continuously developing as remote sensing data and methods grow. However, there still exists low consistency among LULC products due to low accuracy in some regions and LULC types. Here, we introduce Sentinel2GlobalLULC, a Sentinel-2 RGB image dataset, built from the spatial-temporal consensus of up to 15 global LULC maps available in Google Earth Engine. Sentinel2GlobalLULC v2.1 contains 194877 single-class RGB image tiles organized into 29 LULC classes. Each image is a 224 × 224 pixels tile at 10 × 10 m resolution built as a cloud-free composite from Sentinel-2 images acquired between June 2015 and October 2020. Metadata includes a unique LULC annotation per image, together with level of consensus, reverse geo-referencing, global human modification index, and number of dates used in the composite. Sentinel2GlobalLULC is designed for training deep learning models aiming to build precise and robust global or regional LULC maps.

## Abstract (summary)

**Table:** 

Measurement(s)	land cover • land use
Technology Type(s)	satellite imaging of a planet

## Background & Summary

Land-Use and Land-Cover (LULC) mapping aims to characterize the continuous biophysical properties of the Earth surface as categorical classes of natural or human origin, such as forests, shrublands, grasslands, marshlands, croplands, urban areas or water bodies, etc.^1^. High resolution LULC mapping plays a key role in many fields, from natural resources monitoring, to biodiversity conservation, urban planning, agricultural management or climate and earth system modelling^2–4^. Multiple LULC products have been derived from satellite information at the global scale (Table 2), contributing to a better monitoring, understanding, and territorial planning of our planet^5,6^. However, despite the acceptable global accuracy of each individual product, a considerable disagreement among products has been reported^4,7–22^. These reports explain that this disagreement is due to several methodological reasons, including: (1)Given that different satellite sensors with different spatial resolutions were used in each product, the difference in precision from coarse to fine resolution imagery partially determines the final quality of each product. (2)Different pre-processing techniques, like atmospheric corrections, cloud removal and image composition were used in each product. (3)Each product has a different updating frequency (from regularly to never updated products). (4)Different classification systems (i.e., LULC legends) were adopted in each product, usually each one focusing on a distinct application. (5)Different classification techniques, field-data collection approaches, and subjective interpretations were used to create each product. (6)Different validation techniques and different ground truth reference data were used in each product, which impedes a reliable accuracy comparison.

Over the last few years, several attempts have been made to overcome these inconsistencies with a harmonised approach capable of providing better control in the validation and comparison over the growing number of existing LULC products^23,24^. Even though, users still have some issues regarding appropriate product selection due to the following factors: (1)In most cases, users are unable to find a product that fits their desired LULC class or geographic region of interest^25,26^. (2)These products are usually collected at a coarse resolution, which makes analysis at a finer scale difficult^12^. (3)These products offer a limited number of LULC classes that usually change from one product to another^27^.

In parallel, deep artificial neural networks, also known as Deep Learning (DL), are increasingly used in LULC mapping with promising potential^28^. This interest is motivated by the good performance of DL models in computer vision and, particularly of Convolutional Neural Networks (CNNs) in remote sensing image classification and many applications^29–33^. However, to reach high performance, DL models need to be trained on large smart datasets^34^. The concept of smart data involves all pre-processing methods that improve its data value and veracity, in addition to the quality of its associated expert annotations^35^.

**Fig. 1:**
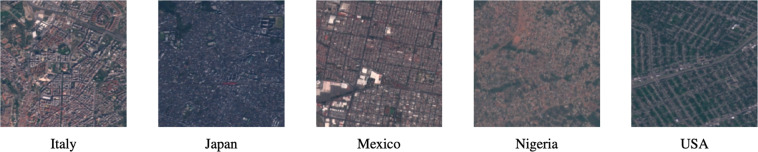
Illustration from different countries of the Sentinel-2 satellite images corresponding to one of the 29 Land-Use and Land-Cover (LULC) classes (e.g. Urban and built-up area) extracted from Sentinel2GlobalLULC dataset. Each image has 224 × 224 pixels of 10 × 10 m resolution. Pixel values were calculated as the 25th-percentile of all images captured between June 2015 and October 2020 that were not tagged as cloudy. Fifteen LULC products available in Google Earth Engine agreed in annotating each image to represent one LULC class.

Currently, there exist several remote sensing datasets derived from satellite and aerial imagery ready for training DL models for LULC mapping (Table 1). However, they still suffer from some limitations, particularly the following factors that complicate their application with DL models: (1)First, none of them represent the global heterogeneity of the broad categories of LULC classes throughout the Earth. Usually, they are biased towards specific regions of the world, limited to national or continental scales, which can propagate such bias to the DL models^36–38^. As illustration, the reader can see how visual features of urban areas may change from one country to another (Fig. 1). (2)Second, they are relatively small and have only hundreds to few thousands of annotated data records^39^. (3)Third, they suffer from high variability in atmospheric conditions, and they have high inter-class similarity and intra-class variability, which makes their class differentiation difficult^39^.

**Table 1:** List of existing Land-Use and Land-Cover (LULC) datasets ready for training Deep Learning (DL) models.

Dataset	Source	Source mapping type	Number of images	Image Size	Spatial Resolution	No. Bands	No. Classes	Extent
ISPRS Vaihingen^56^	—	Airborne	33 im	2000 × 2000	0.09	3	6	Local
ISPRS Postdam^56^	—	Airborne	38 im	6000 × 6000	0.09	3	6	Local
Brazilian coffee scenes^57^	SPOT-5	Spaceborne	50,004 im	64 × 64	10	3	3	Local
SAT-4^58^	NAIP program	Airborne	500,000 im	28 × 28	1	4	4	Local
SAT-6^58^	NAIP program	Airborne	405,000 im	28 × 28	1	4	6	Local
UCMerced^59^	OPLS	Airborne	2100 im	256 × 256	0.3	4	21	Local
Zeebruges (link)	LiDAR	Airborne	100,000 im	10 × 10	0.05	3	8	Local
WHU-RS19^60^	Google Earth	Airborne	1005 im	600 × 600	Up to 0.5	3	19	Local
SIRI-WHU^61^	Google Earth	Airborne	2.240 im	200 × 200	2	3	12	Local
RSSCN7^62^	Google Earth	Airborne	2800 im	400 × 400	—	3	7	Local
RSC11 (link)	Google Earth	Airborne	1232 im	512 × 512	0.2	3	11	Local
NWPU-RESISC45^18^	—	—	31,500 im	256 × 256	$\widetilde{3}0$–0.2	3	45	Local
AID^63^	Google Earth	Airborne	10,000 im	600 × 600	$\widetilde{8}$-0.5	3	30	Local
BigEarthNet^19^	Sentinel-2	Satellite	590,326 img.	—	—	—	—	10 European countries
SpaceNet-7^64^	Dove Satellite Constellation Planet Labs’	Satellite	img.	—	—	—	—	100 cities

To overcome these limitations, we introduce in this paper Sentinel2GlobalLULC^40^, a smart dataset with 29 annotated LULC classes at global scale built with Sentinel-2 RGB imagery. Every image in this dataset is geo-referenced and has a unique LULC annotation. Each image label was carefully built from a consensus approach by combining up to 15 global LULC maps available in Google Earth Engine(GEE)^41^. We released a tif and jpeg version of each image and a CSV file for each LULC class containing the coordinates of each image center, and additional metadata. Sentinel2GlobalLULC aims to foster the creation of accurate global LULC products by exploiting the currently offered advantages by DL. Sentinel2GlobalLULC could be used to train and/or evaluate DL based models for global LULC mapping. We expect this dataset to improve our understanding and modelling of natural and human systems around the world.

## Methods

To build Sentinel2GlobalLULC, we followed two main steps. First, we established a spatio-temporal consensus between 15 global LULC products for 29 LULC classes. Then, we extracted the maximum number of Sentinel-2 RGB images representing each class. Each image is a tile that has 224 × 224 pixels at 10 × 10 m spatial resolution and was built as a cloud-free composite from all the Sentinel-2 images acquired between June 2015 and October 2020. Both tasks were implemented using GEE, an efficient programming, processing and visualisation platform that allowed us to have free manipulation and access to all used LULC products and Sentinel-2 imagery, simultaneously.

### Finding spatio-temporal agreement across 15 global LULC products

To establish the spatio-temporal consensus between different LULC products for each one of the 29 LULC classes, we followed four steps: (1)Identification of the LULC products to be used in the consensus, (2)Standardization and harmonization of the LULC legend that was subsequently used to annotate the image tiles, (3)Spatio-temporal aggregation across LULC products, and (4)Spatial reprojection and tile selection based on optimized spatial purity thresholds.

#### Global LULC products selection

The adopted purity measure for spatio-temporal agreement across the 15 global LULC products we selected from GEE (Table 2) aims to find areas of high consensus to maximize the annotation quality. Spatial and temporal consensus across such rich diversity of LULC products, in terms of spatial resolution, time coverage, satellite source, LULC classes and accuracy, was used as a source of robustness for our subsequent LULC annotation. Products outside GEE were not used due to computing limitations.

**Table 2:** Main characteristics of the 15 global Land-Use and Land-Cover (LULC) products available in Google Earth Engine (GEE) that were combined to find consensus in the global distribution of 29 main LULC classes.

LULC product	Satellite or Spaceborne	Resolution	Used years	Reference
P1: MCD12Q1.006 MODIS LULC Type Yearly Global 500 m LULC Type1: Annual International Geosphere-Biosphere Programme (IGBP) classification (version 6)	Aqua, Terra	500 meters	2017 to 2019	^65^
P2: MCD12Q1.006 MODIS LULC Type Yearly Global 500 m LULC Type 2: Annual University of Maryland (UMD) classification (version 6)	Aqua, Terra	500 meters	2017 to 2019	^65^
P3: MCD12Q1.006 MODIS LULC Type Yearly Global 500 m LULC Type 3: Annual Leaf Area Index (LAI) classification (version 6)	Aqua, Terra	500 meters	2017 to 2019	^65^
P4: MCD12Q1.006 MODIS LULC Type Yearly Global 500 m LULC Type 4: Annual BIOME-Biogeochemical Cycles (BGC) classification (version 6)	Aqua, Terra	500 meters	2017 to 2019	^65^
P5: MCD12Q1.006 MODIS LULC Type Yearly Global 500 m LULC Type 5: Annual Plant Functional Types (PFT) classification (version 6)	Aqua, Terra	500 meters	2017 to 2019	^65^
P6: Copernicus Global LULC Layers: CGLS-LC100 collection 3 (version 3.0.1)	PROBA-V	100 meters	2017 to 2019	^66^
P7: Global Forest Cover Change (GFCC) Tree Cover Multi-Year Global 30 m (version 3.0)	Multi-satellite	30 meters	2015	^67^
P8: GlobCover: Global LULC Map (version 2.0)	ENVISAT	300 meters	2009	ESA 2010 and UCLouvain
P9: GFSAD1000: Cropland Extent 1 km Multi-Study Crop Mask, Global Food-Support Analysis Data (version 0.1)	Multi-satellite	1000 meters	2010	^68^
P10: Global PALSAR-2/PALSAR Forest/Non-Forest Map (version fnf)	ALOS, ALOS 2	25 meters	2017	^69^
P11: Hansen Global Forest Change (version 1.7)	Landsat 8	1 arc seconds	2000 to 2019	^70^
P12: Global Forest Canopy Height (version 2005)	Lidar	30 arc seconds	2005	^71^
P13: JRC Yearly Water Classification History (version 1.2)	Landsat (5,7,8)	30 meters	2017 to 2019	^72^
P14: JRC Global Surface Water Mapping Layers (version 1.2)	Landsat(5,7,8)	30 meters	1984 to 2019	^72^
P15: Tsinghua FROM-GLC year of change to impervious surface(version 10)	Landsat	30 meters	1985 to 2019	^73^

#### Standardization and Harmonization of LULC legends

Land cover (LC) data describes the main type of natural ecosystem that occupies an area; either by vegetation types such as shrublands, grasslands and forests, or by other biophysical classes such as permanent snow, bare land and water bodies. Land use (LU) includes the way in which humans modify or exploit an area, such as urban areas or agricultural fields.

**Fig. 2:**
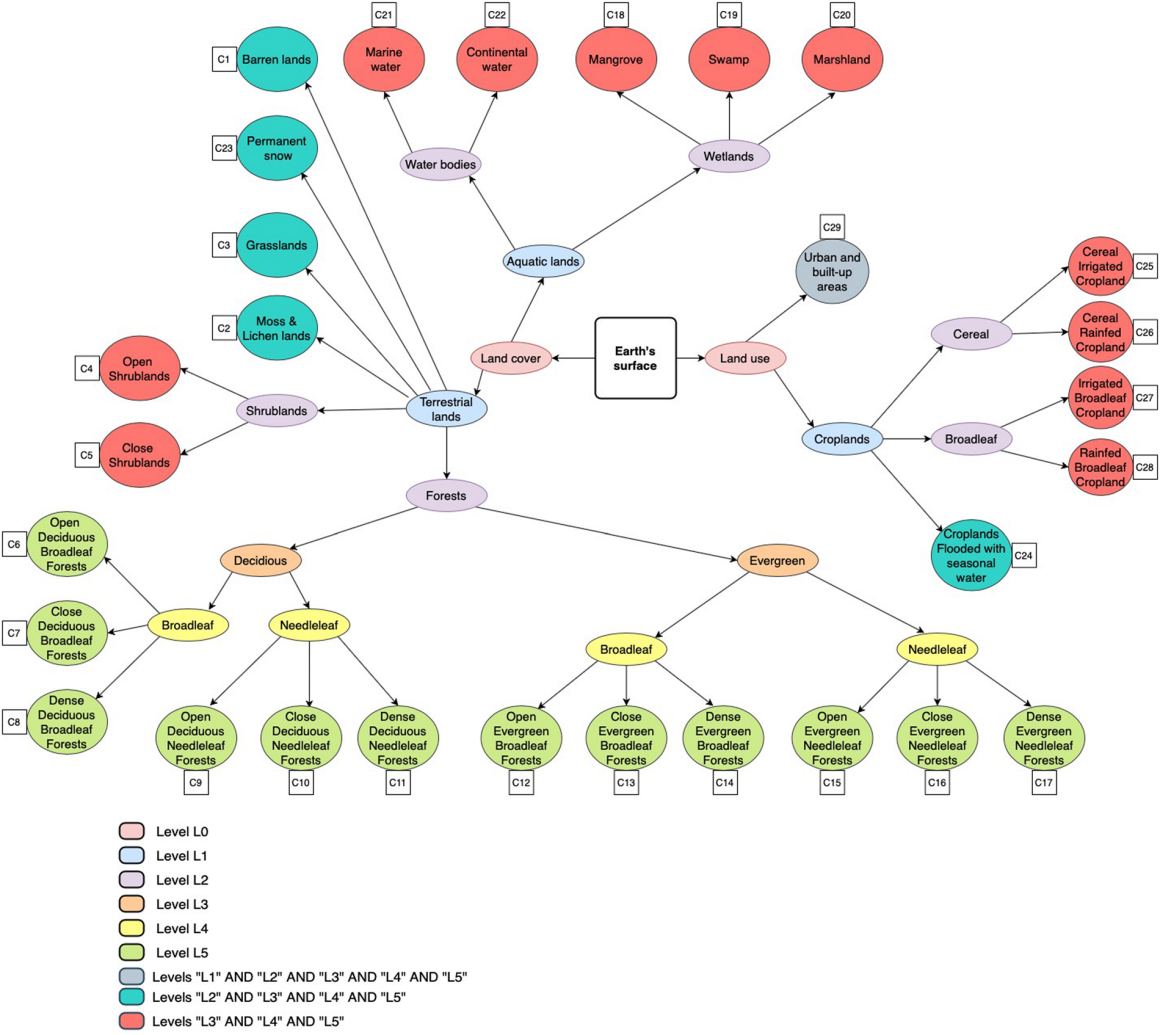
Tree representation of the six-level (L0 to L5) hierarchical structure of the Land-Use and Land-Cover (LULC) classes contained in the Sentinel2GlobalLULC dataset. Outter circular leafs represent the final or most detailed 29 LULC classes (C1 to C29) of level L5. The followed path to define each class is represented through inner ellipses that contain the names of intermediate classes at different levels between the division of the Earth’s surface (square) into LU and LC (level L0) and the final class circle (level L5). All LULC classes belong to three levels at least, except the 12 forest classes that belong to L5 only.

To build our 29 LULC classes nomenclature, we established a standardization and harmonization approach based on expert knowledge. During this process, we took into account both the needs of different practitioners in the global and regional LULC mapping field and the thematic resolution of the global LULC legends available in GEE. Our nomenclature consists of 23 LC and 6 LU distinct classes identified through specific consensus rules across 15 LULC products (see Table 4). A six-level (L0 to L5) hierarchical structure was adopted in the creation of these 29 LULC classes (Fig. 2). To facilitate the inter-operability of our 29 legends at the finest level L5 across all LULC products and with the widely used FAO’s hierarchical Land Cover Classification System (LCCS)^1^, we have established an LULC classification system where the 29 classes can be mapped directly to FAO’s LCCS as explained in the table of Supplementary File 1. The LC part in our dataset contains 20 terrestrial ecosystems and 3 aquatic ecosystems. The terrestrial systems are: Barren lands, Grasslands, Permanent snow, Moss and Lichen lands, Close shrublands, Open shrublands, in addition to 12 Forests classes that differed in their tree cover, phenology, and leaf type. The aquatic classes are: Marine water bodies, Continental water bodies, and Wetlands; furthermore, wetlands were divided into 3 classes: Marshlands, Mangroves and Swamps. The LU part is composed of urban areas and 5 coarse cropland types that differed in their irrigation regime and leaf type. In Table 3, you can find the semantic definition of each one of the 29 classes in Sentinel2GlobalLULC. We provided a table in Supplementary File 2, for a more detailed definition of each LULC class.

**Table 3:** Semantic signification of each one of the 29 Land Use and Land Cover (LULC) classes contained in the Sentinel2GlobalLULC dataset according to the six-level (L0 to L5) hierarchical structure.

L0	L1	L2	L3	L4	L5	Semantic definition
Land Cover	Terrestrial Lands	C1 BarrenLands				Bare land where at least 60% of the surface are non-vegetated barren areas (sand, rock, soil) with <10% of vegetation cover, <10% of tree cover, without gains or losses of tree cover during the study period, tree height <1 m, not cropped or urbanized, and never covered by seasonal or permanent water
		C2 MossAndLichen				Lands vegetated by mosses and lichens where at least 60% of the surface is non-vegetated barren land with <10% of vegetation cover, <10% of tree cover, without gains or losses of tree cover during the study period, tree height <1 m, not cropped or urbanized, and never covered by seasonal or permanent water
		C3 Grasslands				Grasslands dominated by herbaceous annuals (<2 m height), including plants without persistent stem, where tree and shrub cover are <10%, without gains or losses of tree cover during the study period, tree height <1 m, not cropped or urbanized, and never covered by seasonal or permanent water
		Shrublands	C4 ShrublandOpen			Open shrublands dominated by woody perennials with persistent and woody stems (1-2 m height) with a shrub cover between 10% and 60%, tree cover <10%, without gains or losses of tree cover during the study period, tree height <2 m, not cropped or urbanized, and never covered by seasonal or permanent water
			C5 SrublandClose			Close shrublands dominated by woody perennials with persistent and woody stems (1-2 m height) with a shrub cover >60%, tree cover <10%, without gains or losses of tree cover during the study period, tree height <2 m, not cropped or urbanized, and never covered by seasonal or permanent water
		Forests	ForestsDe	ForestsDeBr	C6 ForestsOpDeBr	Forests dominated by deciduous broadleaf trees with tree cover between 15% and 30%, tree height >2 m, without gains or losses of tree cover during the study period, not urbanized, and never covered by seasonal or permanent water
					C7 ForestsClDeBr	Forests dominated by deciduous broadleaf trees with tree cover between 40% and 60%, tree height >2 m, without gains or losses of tree cover during the study period, not urbanized, and never covered by seasonal or permanent water
					C8 ForestsDeDeBr	Forests dominated by deciduous broadleaf trees with tree cover >60%, tree height >2 m, without gains or losses of tree cover during the study period, not urbanized, and never covered by seasonal or permanent water
				ForestsDeNe	C9 ForestsOpDeNe	Forests dominated by deciduous needleleaf larch trees with tree cover between 15% and 30%, tree height >2 m, without gains or losses of tree cover during the study period, not urbanized, and never covered by seasonal or permanent water
					C10 ForestsClDeNe	Forests dominated by deciduous needleleaf larch trees with tree cover between 40% and 60%, tree height >2 m, without gains or losses of tree cover during the study period, not urbanized, and never covered by seasonal or permanent water
					C11 ForestsDeDeNe	Forests dominated by deciduous needleleaf larch trees with tree cover >60%, tree height >2 m, without gains or losses of tree cover during the study period, not urbanized, and never covered by seasonal or permanent water
			ForestsEv	ForestsEvBr	C12 ForestsOpEvBr	Forests dominated by evergreen broadleaf trees with tree cover between 15% and 30%, tree height >2 m, without gains or losses of tree cover during the study period, not urbanized, and never covered by seasonal or permanent water
					C13 ForestsClEvBr	Forests dominated by evergreen broadleaf trees with tree cover between 40% and 60%, tree height >2 m, without gains or losses of tree cover during the study period, not urbanized, and never covered by seasonal or permanent water
					C14 ForestsDeEvBr	Forests dominated by evergreen broadleaf trees with tree cover >60%, tree height >2 m, without gains or losses of tree cover during the study period, not urbanized, and never covered by seasonal or permanent water
				ForestsEvNe	C15 ForestsOpEvNe	Forests dominated by evergreen needleleaf conifer trees with tree cover between 15% and 30%, tree height >2 m, without gains or losses of tree cover during the study period, not urbanized, and never covered by seasonal or permanent water
					C16 ForestsClEvNe	Forests dominated by evergreen needleleaf conifer trees with tree cover between 40% and 60%, tree height >2 m, without gains or losses of tree cover during the study period, not urbanized, and never covered by seasonal or permanent water
					C17 ForestsDeEvNe	Forests dominated by evergreen needleleaf conifer trees with tree cover >60%, tree height >2 m, without gains or losses of tree cover during the study period, not urbanized, and never covered by seasonal or permanent water
		C23 PermanentSnow				Permanent snow and ice where at least 60% of area is covered by snow and ice for at least 10 moths of the year, not urbanized, and never covered by seasonal or permanent water
	Aquatic Lands	Wetlands	C18 WetlandMangro			Permanently inundated lands with seasonal or permanent water with a water cover between 30 and 60% and tree cover >10%, containing closed (>40%) broadleaved semi-deciduous and/or evergreen forest regularly flooded with saline water, tree height >2 m, without gains or losses of tree cover during the study period, and not urbanized
			C19 WetlandSwamps			Permanently inundated lands with seasonal or permanent water with a water cover between 30 and 60% and tree cover >10%, containing closed (>40%) broadleaved forest regularly flooded with freshwater, or closed to open (>15%) vegetation like grassland, shrubland, woody vegetation on regularly flooded, or waterlogged soil with fresh, brackish or saline water but strictly different from closed (>40%) broadleaved semi-deciduous and/or evergreen forest regularly flooded with saline water. Tree height >2 m, without gains or losses of tree cover during the study period, and not urbanized
			C20 WetlandMarshl			Permanently inundated lands with seasonal or permanent water with a water cover between 30 and 60%, tree cover 10% containing closed to open (>15%) vegetation like grassland, shrubland, woody vegetation on regularly flooded or waterlogged soil with fresh, brackish or saline water, tree height >2 m, without gains or losses of tree cover during the study period, and not urbanized
		WaterBody	C21 WaterBodyMari			Water bodies (oceans and seas) where at least 60% of area is covered by permanent water bodies, and not urbanized
			C22 WaterBodyCont			Water bodies (lakes, reservoirs and rivers, can be either fresh or salt-water bodies) where at least 60% of area is covered by permanent water bodies, and not urbanized
Land Use	Croplands	C24 CropSeasWater				Croplands flooded with seasonal, ephemeral seasonal or permanent to seasonal water where at least 60% of area is cultivated cropland dominated by herbaceous annuals (<2 m), and not urbanized
		CropCerea	C25 CropCereaIrri			Irrigated cereal croplands where at least 60% of area is cereal cropland dominated by herbaceous annuals (<2 m), with single or multiple season cropping systems under major or minor irrigation, not urbanized, and never covered by seasonal or permanent water
			C26 CropCereaRain			Rainfed cereal croplands where at least 60% of area is cereal cropland dominated by herbaceous annuals (<2 m), with single or multiple season cropping systems under rainfed or with minor or very minor fragments of rainfed agriculture, not urbanized, and never covered by seasonal or permanent water
		CropBroad	C27 CropBroadIrri			Irrigated broadleaf croplands where at least 60% of area is broadleaf cropland dominated by herbaceous annuals (<2 m), with single or multiple season cropping systems under major or minor irrigation, not urbanized, and never covered by seasonal or permanent water
			C28 CropBroadRain			Rainfed broadleaf croplands with at least 60% of area is broadleaf cropland dominated by herbaceous annuals (<2 m), with single or multiple season cropping systems under rainfed or with minor or very minor fragments of rainfed agriculture, not urbanized, and never covered by seasonal or permanent water
	C29 UrbanBlUpArea					Urban and built-up areas with artificial surfaces and associated areas (urban areas) >50%, with at least 30% of impervious surface area including building materials and asphalt and vehicles, and never covered by seasonal or permanent water

#### Combining products across time and space

For each one of the 29 LULC classes, we combined in space and time the global LULC information among the 15 GEE LULC products. This way, each image was annotated with a LULC class only if all combined products agreed in its corresponding tile (i.e., 100% of agreement in space and time). For each product and LULC type, we first set one or more criteria to create a global mask at the native resolution of the product in which each pixel was classified as 1 or 0 depending on whether it met the criteria for belonging to that LULC type or not, respectively (see first stage in Table 4). For certain LULC classes, some products did not provide any relevant information, so they were not used. For example (Table 4), in Grasslands (C3), Open Shrublands (C4) and Close Shrublands (C5), we combined 14 products, while in UrbanBlUpArea (C29) and Permanent Snow (C23) we only combined 10 and 7 products, respectively.

**Table 4:** First stage of the rule set criteria used to find consensus across the 15 Land-Use and Land-Cover (LULC) products available in Google Earth Engine (GEE) for each of the 29 LULC classes contained in the Sentinel2GlobalLULC dataset.

LULC class	P1	P2	P3	P4	P5	P6	P7	P8	P9	P10	P11	P12	P13	P14	P15	Number of Products
C1 BarrenLands	16	15	NA	7	11	60	TCC < 10	200	0	2	(TC < 10) ∩ (G = 0) ∩ (L = 0) ∩ (D≠2)	TH < 1	1 ∪ 0	0	Not(≥1)	14
C2 MossAndLichen	16	15	NA	7	11	NA	TCC < 10	200 ∪ 150	0	2	(TC < 10) ∩ (G = 0) ∩ (L = 0) ∩ (D≠2)	TH < 1	1 ∪ 0	0	Not(≥1)	13
C3 Grasslands	10	10	1	6	6	30	TCC < 10	140	NA	2	(TC < 10) ∩ (G = 0) ∩ (L = 0) ∩ (D≠2)	TH < 2	1 ∪ 0	0	Not(≥1)	14
C4 ShrublandOpen	7	7	2	NA	5	20 ∩ (10 < SCF < 50)	TCC < 10	150	0	2	(TC < 10) ∩ (G = 0) ∩ (L = 0) ∩ (D≠2)	TH < 2	1 ∪ 0	0	Not(≥1)	14
C5 SrublandClose	6	6	2	NA	5	20 ∩ (SCF > 50)	TCC < 10	130	0	2	(TC < 10) ∩ (G = 0) ∩ (L = 0) ∩ (D≠2)	TH < 2	1 ∪ 0	0	Not(≥1)	14
C6 ForestsOpDeBr	NA	NA	NA	4	4	4 + (15 < TCF < 30)	15 < TCC < 30	60	NA	1	(15 < TC < 30) ∩ (G = 0) ∩ (L = 0) ∩ (D≠2)	TH > 2	1 ∪ 0	0	Not(≥1)	11
C7 ForestsClDeBr	NA	NA	NA	4	4	4 + (40 < TCF < 60)	40 < TCC < 60	50	NA	1	(40 < TC < 60) ∩ (G = 0) ∩ (L = 0) ∩ (D≠2)	TH > 2	1 ∪ 0	0	Not(≥1)	11
C8 ForestsDeDeBr	4	4	6	4	4	4 + (TCF > 60)	TCC > 60	50	NA	1	(TC > 60) ∩ (G = 0) ∩ (L = 0) ∩ (D≠2)	TH > 2	1 ∪ 0	0	Not(≥1)	14
C9 ForestsOpDeNe	NA	NA	NA	3	3	3 + (15 < TCF < 30)	15 < TCC < 30	NA	NA	1	(15 < TC < 30) ∩ (G = 0) ∩ (L = 0) ∩ (D≠2)	TH > 2	1 ∪ 0	0	Not(≥1)	10
C10 ForestsClDeNe	NA	NA	NA	3	3	3 + (40 < TCF < 60)	40 < TCC < 60	NA	NA	1	(40 < TC < 60) ∩ (G = 0) ∩ (L = 0) ∩ (D≠2)	TH > 2	1 ∪ 0	0	Not(≥1)	10
C11 ForestsDeDeNe	3	3	8	3	3	3 + (TCF > 60)	TCC > 60	NA	NA	1	(TC > 60) ∩ (G = 0) ∩ (L = 0) ∩ (D≠2)	TH > 2	1 ∪ 0	0	Not(≥1)	13
C12 ForestsOpEvBr	NA	NA	NA	2	2	2 + (15 < TCF < 30)	15 < TCC < 30	40	NA	1	(15 < TC < 30) ∩ (G = 0) ∩ (L = 0) ∩ (D≠2)	TH > 2	1 ∪ 0	0	Not(≥1)	11
C13 ForestsClEvBr	NA	NA	NA	2	2	2 + (40 < TCF < 60)	40 < TCC < 60	40	NA	1	(40 < TC < 60) ∩ (G = 0) ∩ (L = 0) ∩ (D≠2)	TH > 2	1 ∪ 0	0	Not(≥1)	11
C14 ForestsDeEvBr	2	2	5	2	2	2 + (TCF > 60)	TCC > 60	40	NA	1	(TC > 60) ∩ (G = 0) ∩ (L = 0) ∩ (D≠2)	TH > 2	1 ∪ 0	0	Not(≥1)	14
C15 ForestsOpEvNe	9	9	NA	1	1	1 + (15 < TCF < 30)	15 < TCC < 30	90	NA	1	(15 < TC < 30) ∩ (G = 0) ∩ (L = 0) ∩ (D≠2)	TH > 2	1 ∪ 0	0	Not(≥1)	13
C16 ForestsClEvNe	8	8	4	1	1	1 + (40 < TCF < 60)	40 < TCC < 60	70	NA	1	(40 < TC < 60) ∩ (G = 0) ∩ (L = 0) ∩ (D≠2)	TH > 2	1 ∪ 0	0	Not(≥1)	14
C17 ForestsDeEvNe	1	1	7	1	1	1 + (TCF > 60)	TCC > 60	70	NA	1	(TC > 60) ∩ (G = 0) ∩ (L = 0) ∩ (D≠2)	TH > 2	1 ∪ 0	0	Not(≥1)	14
C18 WetlandMangro	11	11	NA	NA	NA	90	TCC > 10	170	NA	NA	(TC > 10) ∩ (G = 0) ∩ (L = 0) ∪ (D = 2)	TH > 2	2 ∪ 3	1	Not(≥1)	10
C19 WetlandSwamps	11	11	NA	NA	NA	90	TCC > 10	CritP8a:160 ∪ 180CritP8b:Not(170)	NA	NA	(TC > 10) ∩ (G = 0) ∩ (L = 0) ∪ (D = 2)	TH > 2	2 ∪ 3	1	Not(≥1)	10
C20 WetlandMarshl	11	11	NA	NA	NA	90	TCC < 10	160 ∪ 170 ∪180	NA	NA	(TC<10) ∩ (G=0) ∩ (L=0) ∪ (D=2)	TH > 2	2 ∪ 3		Not(≥1)	10
C21 WaterBodyMari	17	0	0	0	0	200	NA	210	NA	3	NA	NA	3	1	Not(≥1)	11
C22 WaterBodyCont	17	0	0	0	0	80	NA	210	NA	3	NA	NA	3	1	Not(≥1)	11
C23 PermanentSnow	15	NA	NA	NA	10	70	NA	220	NA	NA	NA	NA	1 ∪ 0	0	Not(≥1)	7
C24 CropSeasWater	12	12	3 ∪ 1	5 ∪ 6	7 ∪ 8	40	NA	11 ∪ 14	1 ∪ 2 ∪ 3 ∪ 4 ∪ 5	NA	NA	NA	2 ∪ 3	0 ∪ 4 ∪ ∪ 8 ∪ 10	Not( ≥ 1)	11
C25 CropCereaIrri	12	12	1	6	7	40	NA	11	1 ∪ 2	NA	NA	NA	1 ∪ 0	0	Not( ≥ 1)	11
C26 CropCereaRain	12	12	1	6	7	40	NA	14	3 ∪ 4 ∪ 5	NA	NA	NA	1 ∪ 0	0	Not( ≥ 1)	11
C27 CropBroadIrri	12	12	3	5	8	40	NA	11	1 ∪ 2	NA	NA	NA	1 ∪ 0	0	Not( ≥ 1)	11
C28 CropBroadRain	12	12	3	5	8	40	NA	14	3 ∪ 4 ∪ 5	NA	NA	NA	1 ∪ 0	0	Not( ≥ 1)	11
C29 UrbanBlUpArea	13	13	10	8	9	50	NA	190	NA	NA	NA	NA	1 ∪ 0	0	NU	10

P1 to P15: product 1 to 15. C1 to C29: class 1 to class 29. For each LULC class, we used a different number of products, and in the last column we present the total number of used products per class. For each product, one or multiple criteria were established to create a global probability map (pixel values 0 or 1) for a given LULC class. The numbers in each column (i.e., from 0 to 220) correspond to the pixel values from each product band. NU: Not Used, NA: Not Available, TC: Tree Cover, G: Tree Gain, L: Tree Loss, D: Datamask, TH: Tree Hight, TCC: Tree Canopy Cover, TCF: Tree-Cover Fraction, and SCF: Shrub-Cover Fraction. ∩ :“AND”, ∪ :“OR”, + :“ADD”. In Supplementary File 2, we explain the signification of each on of these criteria in details. (In class C19, the product P8 was used at two different steps of the consensus with a distinct rule in each one: *CritP8a* and *CritP8b*. We explain their utilisation at each step of C19 consensus in its corresponding row within Table 5).

**Fig. 3:**
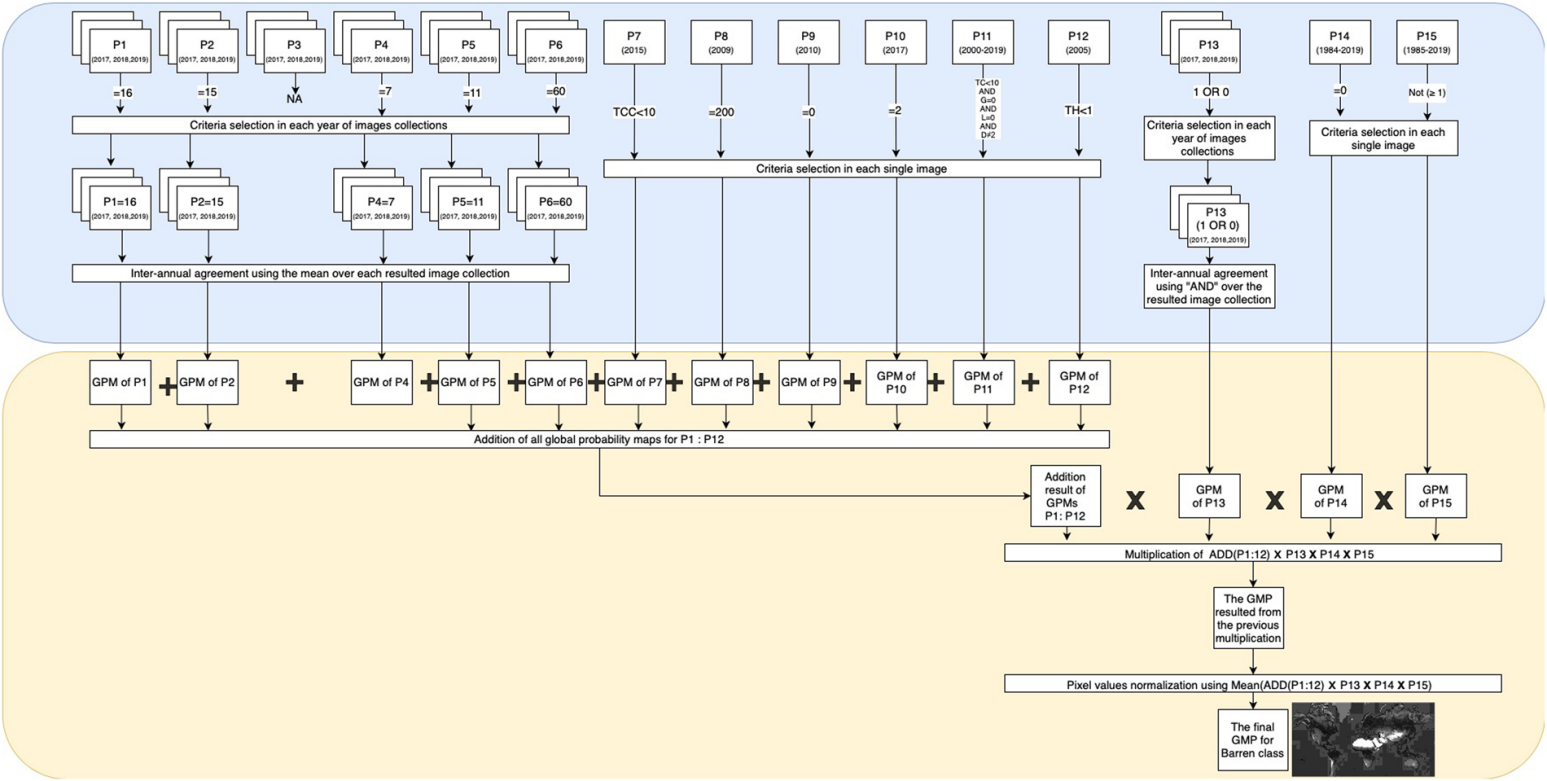
Example of the process of building the final global probability map for one of the 29 Land-Use and Land-Cover (LULC) classes (e.g. C1: "Barren") by means of spatio-temporal agreement of the 15 LULC products available in Google Earth Engine (GEE). The final map is normalized to values between 0 (white, i.e., areas with no presence of C1 in any product) and 1 (black spots, i.e., areas containing or compatible with the presence of C1 in all 15 products), whereas the shades of grey corresponds to the values in between (i.e., areas that did not contain or were not compatible with the presence of C1 in some of the products). This process is divided into two stages: the first stage (the blue part, see details in Table 4) and the second stage (the yellow part, see details in Table 5). LULC products available for several years are represented with superposed rectangles, while single year products are represented with single rectangles. GMP: global probability map, NA: Not Available.

Then (see second stage in Table 5), for each LULC type, we calculated the average of all the masks obtained from each product to create a final global probability map from all products with values ranging between 0 and 1. Value 1 meant that all products agreed to assign that pixel to a particular LULC class, while 0 meant that none of the products assigned it to that particular class (Fig. 3). These 0-to-1 values are interpreted as the spatio-temporal purity level of each pixel to belong to a particular LULC class and are provided as metadata with each image.

**Table 5:** Second stage of the rule set criteria used to find consensus across the 15 Land-Use and Land-Cover (LULC) products available in Google Earth Engine (GEE) for each of the 29 LULC classes contained in the Sentinel2GlobalLULC dataset.

Class ID	LULC class	Spatial Combination
C1	Barren lands	Norm(P15*P14*P13*(Add(P1,P2,P4:P12)))
C2	Moss and Lichen lands	Norm(P15*P14*P13*(Add(P1,P2,P4,P5,P7:P12)))
C3	Grasslands	Norm(P15*P14*P13*(Add(P1:P8,P10:P12)))
C4	Open Shrublands	Norm(P15*P14*P13*(Add(P1:P3,P5:P12)))
C5	Close Shrublands	Norm(P15*P14*P13*(Add(P1:P3,P5:P12)))
C6	Open Deciduous Broadleaf Forests	Norm(P15*P14*P13*(Add(P4:P8,P10:P12)))
C7	Close Deciduous Broadleaf Forests	Norm(P15*P14*P13*(Add(P4:P8,P10:P12)))
C8	Dense Deciduous Broadleaf Forests	Norm(P15*P14*P13*(Add(P1:P8,P10:P12)))
C9	Open Deciduous Needleleaf Forests	Norm(P15*P14*P13*(Add(P4:P7,P10:P12)))
C10	Close Deciduous Needleleaf Forests	Norm(P15*P14*P13*(Add(P4:P7,P10:P12)))
C11	Dense Deciduous Needleleaf Forests	Norm(P15*P14*P13*(Add(P1:P7,P10:P12)))
C12	Open Evergreen Broadleaf Forests	Norm(P15*P14*P13*(Add(P4:P8,P10:P12)))
C13	Close Evergreen Broadleaf Forests	Norm(P15*P14*P13*(Add(P4:P8,P10:P12)))
C14	Dense Evergreen Broadleaf Forests	Norm(P15*P14*P13*(Add(P1:P8,P10:P12)))
C15	Open Evergreen Needleleaf Forests	Norm(P15*P14*P13*(Add(P1,P2,P4:P8,P10:P12)))
C16	Close Evergreen Needleleaf Forests	Norm(P15*P14*P13*(Add(P1:P8,P10:P12)))
C17	Dense Evergreen Needleleaf Forests	Norm(P15*P14*P13*(Add(P1:P8,P10:P12)))
C18	Mangrove Wetlands	Norm(P15*(Add(P1,P2,P6:P8,P11:P14)))
C19	Swamp Wetlands	Norm(P15*P8b*(Add(P1,P2,P6,P7,P8a,P11:P14)))
C20	Marshland Wetlands	Norm(P15*(P11 OR P12 OR P7)*(Add(P1,P2,P6,P8,P13,P14)))
C21	Marine Water Bodies	Norm(P15*P14*P13*(Add(P1:P6,P8,P10)))
C22	Continental Water Bodies	Norm(P15*P14*P13*(Add(P1:P6,P8,P10)))
C23	Permanent Snow	Norm(P15*P14*P13*(Add(P1,P5,P6,P8)))
C24	Croplands Flooded with seasonal water	Norm(P15*(P13 OR P14)*(Add(P1:P6,P8,P9)))
C25	Cereal Irrigated Cropland	Norm(P15*P14*P13*(Add(P1:P6,P8,P9)))
C26	Cereal Rainfed Cropland	Norm(P15*P14*P13*(Add(P1:P6,P8,P9)))
C27	Irrigated Broadleaf Cropland	Norm(P15*P14*P13(Add(P1:P6,P8,P9)))
C28	Rainfed Broadleaf Cropland	Norm(P15*P14*P13(Add(P1:P6,P8,P9)))
C29	Urban and built-up areas	Norm(P14*P13(Add(P1:P6,P8)))

P1 to P15: product 1 to 15. C1 to C29: class 1 to class 29. For each LULC class, the 15 global probability maps (with pixel values 0 or 1) obtained in the first stage from products P1 to P15 were spatially combined to build 29 final global probability maps (with pixel values 0 to 1), one for each LULC class (C1 to C29). “Add”:ADD, “*”:MULTIPLY, “Norm”: the normalization using division by number of used products.(The aggregation order in the column “Spatial Combination” is the real adopted order of products aggregation in GEE operations. In class C19, two different global probability maps from product P8 were used: P8a and P8b. Each one of these two maps was generated with a distinct selection rule: *CritP8a* and *CritP8b* respectively. Both rules are explicitly represented in row C19 of Table 4).

As an example of the first stage (see details in Table 4), to specify if a given pixel belongs to Dense Evergreen Needleleaf Forest, we evaluated its tree cover level using “ ≤ “ and “ ≥ “, while for bands containing the leaf type information, we used the equal operator “ = “. For the spatio-temporal combination of multiple criteria we have used the following operators: “AND”,“OR” and “ADD”. For example, we combined the tree cover percentage criteria with the leaf type criteria using “AND” to select forest pixels that met both conditions. To combine many years instances of the same product, we used “ADD”, except for product P13, where we used “AND” to identify permanent water areas only. Whenever we used the “ADD” operator, we normalized pixel values afterwards to bring it back to a probability interval between 0 and 1 using the division by the total number of combined years or criteria.

In the second stage (see details in Table 5), we combined for each LULC class the 15 global probability maps previously derived from each product to create a final global probability map (Fig. 3). This combination was carried out using various operators such as “ADD”, “MULTIPLY” and “OR”, depending on the LULC type. When “ADD” was used, the final pixel values were normalized by dividing the final addition value of each pixel by the total number of added products. The “MULTIPLY” operator was mostly used at the end, to remove urban areas from non-urban LULC classes, or to remove water from non-water LULCs. The multiplication operator was also adopted to make sure that a certain criteria was respected in the final probability map. For instance, for the swamp class, we multiplied all pixels in the final stage by a water mask where saline water areas have a value of 0 in order to eliminate mangrove from swamp pixels and vice versa. Finally, we used “OR” operator between different water related products to take advantage of the fact that they complement each other in terms of spatial-temporal coverage and accuracy.

In GEE, when two products are aggregated using “ADD”, “MULTIPLY” or any other operator, the output is aggregated at the spatial resolution of the product at the left of the operator. Hence, to maintain the finest spatial resolution in the final probability map, we multiplied everything by product P15 and placed it at the left of the final “MULTIPLY” operation (See Table 5). Hence, all the 29 final probability maps were generated at the P15 spatial resolution of 30 m/pixel (except the urban class C29 which maintained the 30 m/pixel resolution of product P14).

#### Re-projection and Selection of purity threshold

**Fig. 4:**
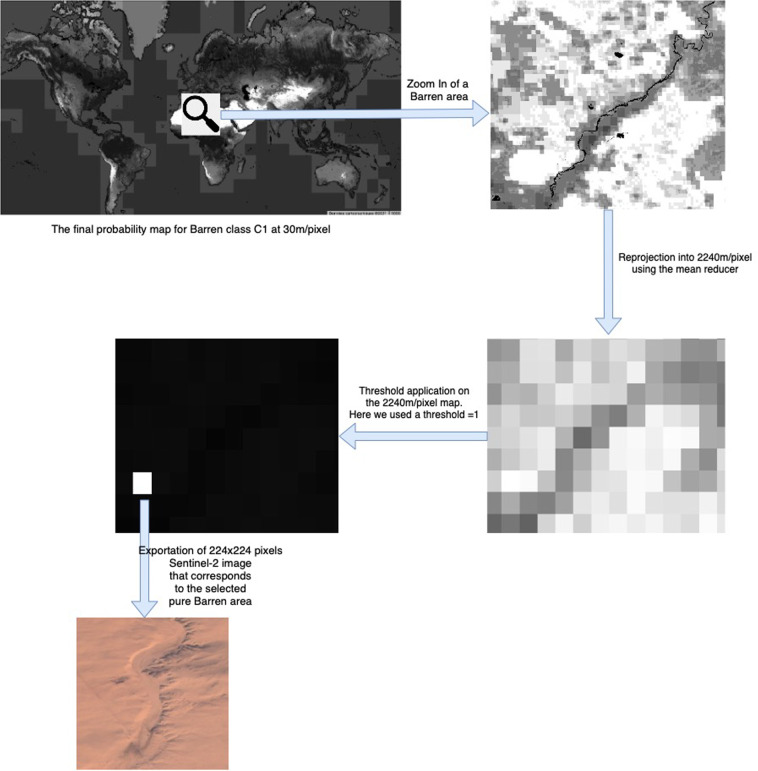
Example of the workflow to obtain a Sentinel-2 image tile of 2240 × 2240 m for one of the 29 Land-Use and Land-Cover (LULC) classes (e.g. C1: “Barren”). The process starts with the reprojected final global probability map obtained from stage two (Table 5) and ends with its exportation to the repository of a Sentinel-2 image tile of 224 × 224 pixels. The white rectangle is the only one having a probability value of 1 (Recall that the purity threshold used for Barren was 1, i.e., 100%). The black pixels has a null probability value, while the probability values between 0 and 1 are represented in gray scale levels.

Since our objective was finding pure Sentinel-2 image tiles of 224 × 224 10-m pixels representing each LULC class, we reprojected the 30 m/pixel probability maps to 2240 m/pixel using the spatial mean reducer in GEE. That is, each pixel value at 2240 m resolution was computed using the mean over all the 30m-pixel values contained within it. Hence, the resulting pixel values at 2240 m resolution represent the purity level that each Sentinel-2 image tile of 224 × 224 10-m pixels has. We illustrated the reprojection and selection processes in Fig. 4.

For each one of the reprojected maps, we defined a pixel value threshold to decide whether a given 2240 × 2240 m tile was representative of each LULC class or not. Since training DL image classification models needs a large number of high quality (both in terms of image quality and annotation quality) image tiles to reach a good accuracy, when the spatial purity of 100% (full agreement across products in all the pixels of the 224 × 224 tile) resulted in a small number of agreement tiles for a particular class, the purity threshold was decreased for that class until the number of tiles was larger than 1000 or further decreased in less abundant classes to a minimum of 75% of purity. The found purity value is always provided as metadata for each image in the dataset, so the user can always restrict its analysis to those image tiles and classes at any desired purity level. Decreasing the purity threshold down to 75% for the less abundant classes (e.g swamp, mangrove, etc.) was a trade-off between maintaining a good data annotation quality and providing a sufficient number of tiles in each class. In Table 6, we present the number of agreement tiles found at different purity thresholds ranging from 75% to 100% for each LULC class. This spatial purity was not further decreased since machine learning image classification models are known to be robust when the target class is spatially dominant in each training image (it occupies more than 60% of the pixels in the scene)^42^. On the other hand, when the number of pure tiles for a LULC class was too large to be downloaded (i.e., greater than 14000), we applied a selection algorithm as described in the Supplementary File 3, to download a maximum of 14000 spatially representative images. For this, the world was divided into a one-degree squared cell grid. If a cell contained less than 50 image tiles, we selected them all. If it contained more than 50, we applied that automatic maximum geographic distance algorithm that selected images as far from each other as possible in a number proportional to the number of existing images in that cell. The map in Fig. 6 shows the global distribution of the selected 194877 image tiles contained in Sentinel2GlobalLULC and distributed in 29 LULC classes.

**Table 6:** Summary of the varying number of found and eventually selected Sentinel-2 image tiles of 224 × 224 pixels depending on the different consensus level reached across the 15 Land-Use and Land-Cover (LULC) products available in Google Earth Engine (GEE) for each of the 29 LULC classes contained in the Sentinel2GlobalLULC dataset.

LCLU Class	Consensus probability values (%)						Number of selected images	Post-selection
	0.75 (75%)	0.80 (80%)	0.85 (85%)	0.90 (90%)	0.95 (95%)	1.00 (100%)		
C29 Urban	63953	50940	34102	21814	12590	192	12590	no
C1 Barren	4330418	4195584	4055836	3876467	3545756	2668009	**14000 (2668009)**	yes
C2 Moss and Lichen	59120	36455	18438	4656	1158	0	4656	no
C5 Close Shrublands	41407	11937	1872	226	16	0	11937	no
C4 Open Shrublands	2461415	1884514	1209375	644272	101288	805	**14000 (101288)**	yes
C20 Marshland	4205	2349	675	143	15	0	4205	no
C19 Swamp	487	164	4	0	0	0	487	no
C18 Mangrove	416	255	63	3	0	0	416	no
C3 Grassland	4022949	3041842	1894337	943177	128263	8869	8869	no
C28 Rainfed Broadleaf Cropland	427314	316696	209143	99337	32123	413	413	no
C27 Irrigated Broadleaf Cropland	224867	144115	92488	53064	30691	353	353	no
C26 Cereal Rainfed Cropland	1185497	911167	604459	284914	91147	1020	1020	no
C25 Cereal Irrigated Cropland	517789	310790	167994	52959	23555	842	842	no
C24 Cropland Seasonal Water	6048	4522	3192	2004	995	15	2004	no
C17 Dense Evergreen Needleleaf Forest	474138	322443	178293	66151	13991	0	13991	no
C16 Close Evergreen Needleleaf Forest	43040	3872	69	0	0	0	3872	no
C15 Open Evergreen Needleleaf Forest	17462	3914	331	0	0	0	3914	no
C14 Dense Evergreen Broadleaf Forest	2131269	1995950	1829897	1594657	1232914	144026	**14000 (144026)**	yes
C13 Close Evergreen Broadleaf Forest	12512	1258	77	1	0	0	1258	no
C12 Open Evergreen Broadleaf Forest	567	42	0	0	0	0	567	no
C11 Dense Deciduous Needleleaf Forest	60866	31414	12954	2880	148	0	2880	no
C10 Close Deciduous Needleleaf Forest	42166	6380	35	0	0	0	6380	no
C9 Open Deciduous Needleleaf Forest	10438	23	0	0	0	0	10438	no
C8 Dense Deciduous Broadleaf Forest	399264	273134	176176	97182	31284	1	**14000 (31284)**	yes
C7 Close Deciduous Broadleaf Forest	71127	12654	1348	23	1	0	1348	no
C6 Open Deciduous Broadleaf Forest	25342	4437	466	2	0	0	4437	no
C23 Permanent Snow	1065127	1049822	1033466	1013490	984014	877232	**14000 (877232)**	yes
C22 Continental Water Bodies	3543953	3327019	3199652	343779	318483	265214	**14000 (265214)**	yes
C21 Marine Water Bodies	3606955	3438966	3357810	2903459	2822544	2577444	**14000 (2577444)**	yes

LULC classes that due to the very large number of image tiles had to undergo a post-selection by maximizing geographical distance between them, are highlighted in bold.

### Data extraction

Sentinel2GlobalLULC provides the user with two types of data: Sentinel-2 RGB images (jpeg and geotif versions) and CSV files with associated metadata. In the following subsections, we describe the process for associating metadata, including the Global Human Modification (GHM) index.

#### Global human modification index extraction

As an additional metadata related to the level of human influence in each image, we calculated for each tile in GEE, the spatial mean of the global human modification index for terrestrial lands^43^, where 0 means no human modification and 1 means complete transformation. Since the original GHM product was mapped at 1 × 1 km resolution, we reprojected it to 2240 × 2240 m using the same reprojection procedure explained in (Re-projection and Selection of purity threshold).

#### CSV files generation

Once the tiles were selected, for each LULC class we listed the image tiles in descendent order of purity. Metadata included: geographical coordinates of each tile centroid, tile purity value, name and ID of the LULC class, and average GHM index for that tile. Then, we used the geographical coordinates of each tile to identify its exact administrative address geolocation. To implement this reverse geo-referencing operation, we used a free request-unlimited python module called reverse_geocoder. This way, we assigned a country code, two levels of administrative departments, and the locality to each tile.

For LULC classes that had more than 14000 pure tiles, we have released the coordinates before and after the distance-based selection in case the user wants to download more tiles or use our consensus coordinates for other purposes.

#### Sentinel-2 RGB images exportation

After extracting all these pieces of information and grouping them into CSV files, we went back to the geographic center coordinates of each tile and used them to extract the corresponding 224 × 224 Sentinel-2 RGB tiles using GEE. Each exported image was identical to the 2240 × 2240 m area covered by its Sentinel-2 tile.

We chose “Sentinel-2 MSI (Multi-Spectral Instrument) product” since it is free and publicly available in GEE at the fine resolution of 10 × 10 m. We chose “Level-1C” (i.e., top-of-atmosphere reflectance) since it provides the longest data availability of Sentinel-2 images without any modification of the data. To build RGB images, we extracted the three bands B4, B3 and B2 that correspond to Red, Green and Blue channels, respectively. More bands available in Sentinel-2 or even in Sentinel-1 images can be incorporated in the future to our dataset. However, computational limitations (i.e., the size of the dataset would be impractical) did not allowed us to handle it as a first goal. In addition, the spatial resolution of the images would be heterogeneous across bands.

To minimize the inherent noise due to atmospheric conditions (e.g. clouds, aerosols, smoke, etc.) that could affect the satellite RGB images, every image was built as a temporal aggregation of all images gathered by Sentinel-2 satellites between June 2015 and October 2020. During this aggregation, only the highest quality images in the corresponding image collection were considered, as we firstly discarded all image instances where the cloud probability exceeded 20% according to the metadata provided in their corresponding Sentinel-2 collection. Then, we calculated the 25th-percentile value between all remaining images for each reflectance band (R, G, and B), and built the final image with the obtained 25-percentile values in each pixel for its RGB bands. The 25th-percentile choice was adopted giving its suitability in atmospheric noise reduction^44–48^.

Usually, Sentinel-2 MSI product includes true colour images in JPEG2000 format, except for the “Level-1C” collection used here. The three original bands (B4, B3, and B2) required a saturation mapping of their reflectance values into 0–255 RGB digital values. Thus, we mapped the saturation reflectance of 3558 into 255 to obtain true RGB channels with digital values between 0 and 255. The choice of these mapping numbers was taken from the Sentinel-2 true colour image recommendations section of Sentinel user guidelines. Finally, after exporting the selected tiles for each LULC class as “.tif” images, we converted them into “.jpeg” format using a lossless conversion algorithm.

### Technical implementation

To implement all our methodology steps, we first created a javascript in GEE for each LULC class. Each script is a multi-task javascript where we implemented a switch command to control which task we want to execute (between the spatio-temporal aggregation task, the spatial reprojection and tiles selection task, or the data exportation task). In each one of these scripts, we selected from GEE LULC datasets repository the 15 LULC products used to build the consensus of that LULC class. Each script was responsible of elaborating the spatio-temporal combination of the selected products and generating the final consensus map for that LULC class as described in the subsection “Combining products across time and space”. Then, it exports the final global probability map as an asset into GEE server storage to make its reprojection faster. In the same script, once the consensus map exportation was done, we imported it from the GEE assets storage and reprojected it to 2240 × 2240 m resolution; then, we exported the new reprojected map into GEE assets storage again to make its analysis and processing faster. Afterwards, we imported the reprojected map into the same script and applied different processing tasks. During this processing phase, many purity threshold values were evaluated. Then, we elaborated in this same script the pure tiles identification and their center coordinates exportation into a CSV file. A distinct GEE script was developed to import, reproject and export the global GHM map. The resulted GHM map was saved as an asset too, then imported and used in each one of the 29 LULC multi-task scripts.

A python script was developed separately to read the exported CSV files for each LULC class and apply the reverse geo-referencing on their pure tiles coordinates then add the found geolocalization data (country code, locality…etc) to the original CSV files as new columns. Then, another python script was implemented to read the new resulted CSV files with all their added columns (reverse geo-referencing data, GHM data) and use the center coordinates of each pure tile in that class to export first its corresponding Sentinel-2 satellite geotiff image within GEE through the python API. Finally, after downloading all the selected geotiff images from our Google drive, we created another python script to convert these geotiff images into JPEG format.

## Data Records

Sentinel2GlobalLULC v2.1^40^ dataset is stored in the following Zenodo repository (10.5281/zenodo.6941662). This dataset consists of three zip compressed folders:

- Sentinel-2 GeoTiff images folder: This folder contains the exported Sentinel-2 RGB images for each LULC class grouped into sub-folders named according to each LULC class. Each image has a filename with the following structure: “LULC class ID_LULC class short name_Pixel probability value_Image ID_GHM value_(Latitude,Longitude)_Country code_Administrative department level1_Administrative department level2_Locality”. Pixel probability value can be interpreted as the spatial purity of the image to represent that LULC class and was calculated as the spatial mean of all the pixels of the final probability maps contained in each image tile, reprojected and expressed as a percentage. Short names for all classes were derived from the original ones in a way to have exactly 13 characters each, and IDs for different classes were assigned randomly. This information for each class is explained in Table 7.

**Table 7:** Dictionary to map each Land-Use and Land-Cover (LULC) class to its corresponding short name and ID in the Sentinel2GlobalLULC dataset.

LCLU Class	Short name	Class ID
Urban	UrbanBlUpArea	C29
Barren	BarrenLands__	C1
Moss and Lichen	MossAndLichen	C2
Close Shrublands	SrublandClose	C5
Open Shrublands	ShrublandOpen	C4
Marshland	WetlandMarshl	C20
Swamp	WetlandSwamps	C19
Mangrove	WetlandMangro	C18
Grassland	Grasslands___	C3
Rainfed Broadleaf Cropland	CropBroadRain	C28
Irrigated Broadleaf Cropland	CropBroadIrri	C27
Cereal Rainfed Cropland	CropCereaRain	C26
Cereal Irrigated Cropland	CropCereaIrri	C25
Cropland Seasonal Water	CropSeasWater	C24
Dense Evergreen Needleleaf Forest	ForestsDeEvNe	C17
Close Evergreen Needleleaf Forest	ForestsClEvNe	C16
Open Evergreen Needleleaf Forest	ForestsOpEvNe	C15
Dense Evergreen Broadleaf Forest	ForestsDeEvBr	C14
Close Evergreen Broadleaf Forest	ForestsClEvBr	C13
Open Evergreen Broadleaf Forest	ForestsOpEvBr	C12
Dense Deciduous Needleleaf Forest	ForestsDeDeNe	C11
Close Deciduous Needleleaf Forest	ForestsClDeNe	C10
Open Deciduous Needleleaf Forest	ForestsOpDeNe	C9
Dense Deciduous Broadleaf Forest	ForestsDeDeBr	C8
Close Deciduous Broadleaf Forest	ForestsClDeBr	C7
Open Deciduous Broadleaf Forest	ForestsOpDeBr	C6
Permanent Snow	PermanentSnow	C23
Continental Water Bodies	WaterBodyCont	C22
Marine Water Bodies	WaterBodyMari	C21
- Sentinel-2 JPEG images folder: This folder contains the same images as in the GeoTiff folder, but converted into “.jpeg” format while preserving the same nomenclature and organization. In Fig. 5, we illustrate an image tile for each one of the 29 classes in JPEG format.

**Fig. 5:**
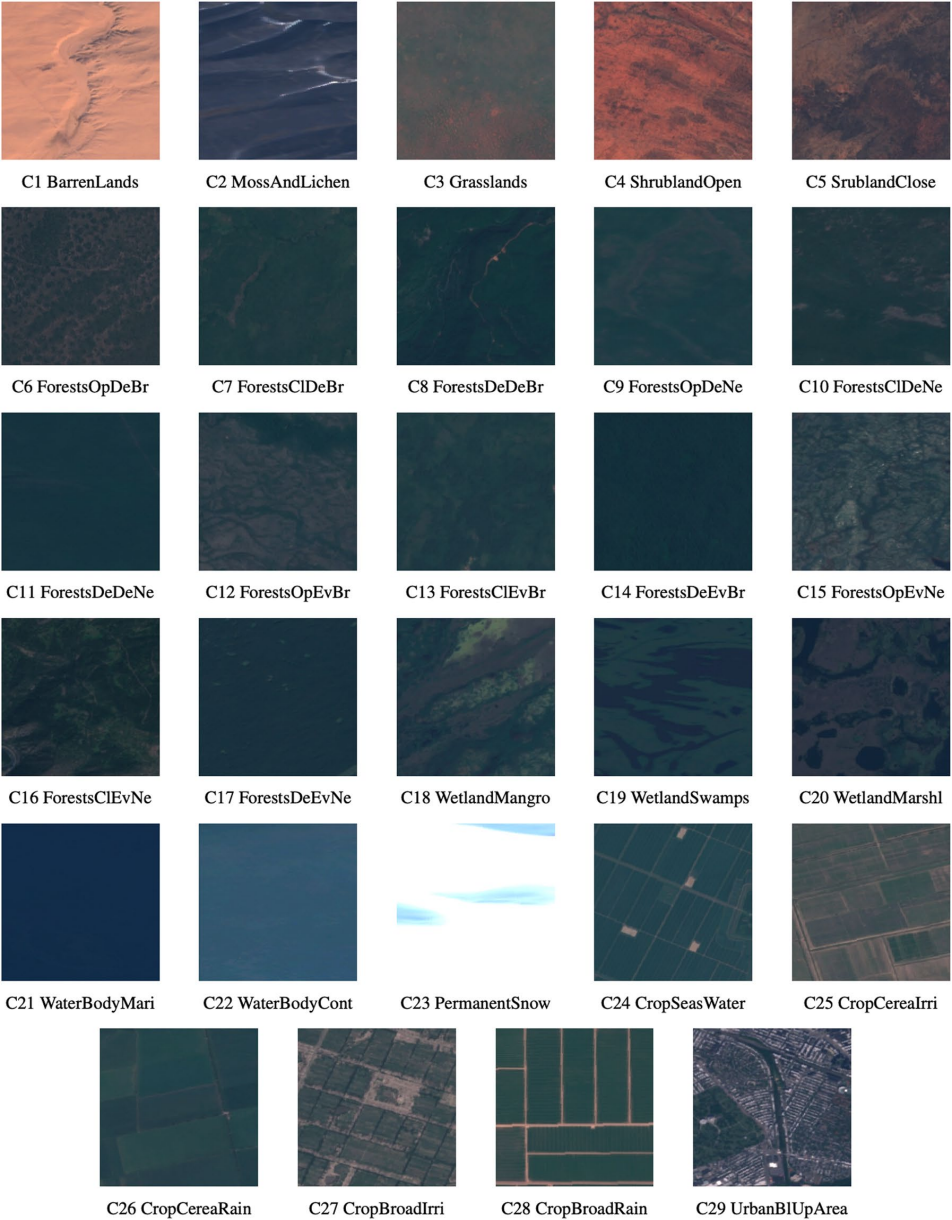
Image tiles examples for each one of the 29 Land-Use and Land-Cover (LULC) classes contained in the Sentinel2GlobalLULC dataset.

**Fig. 6:**
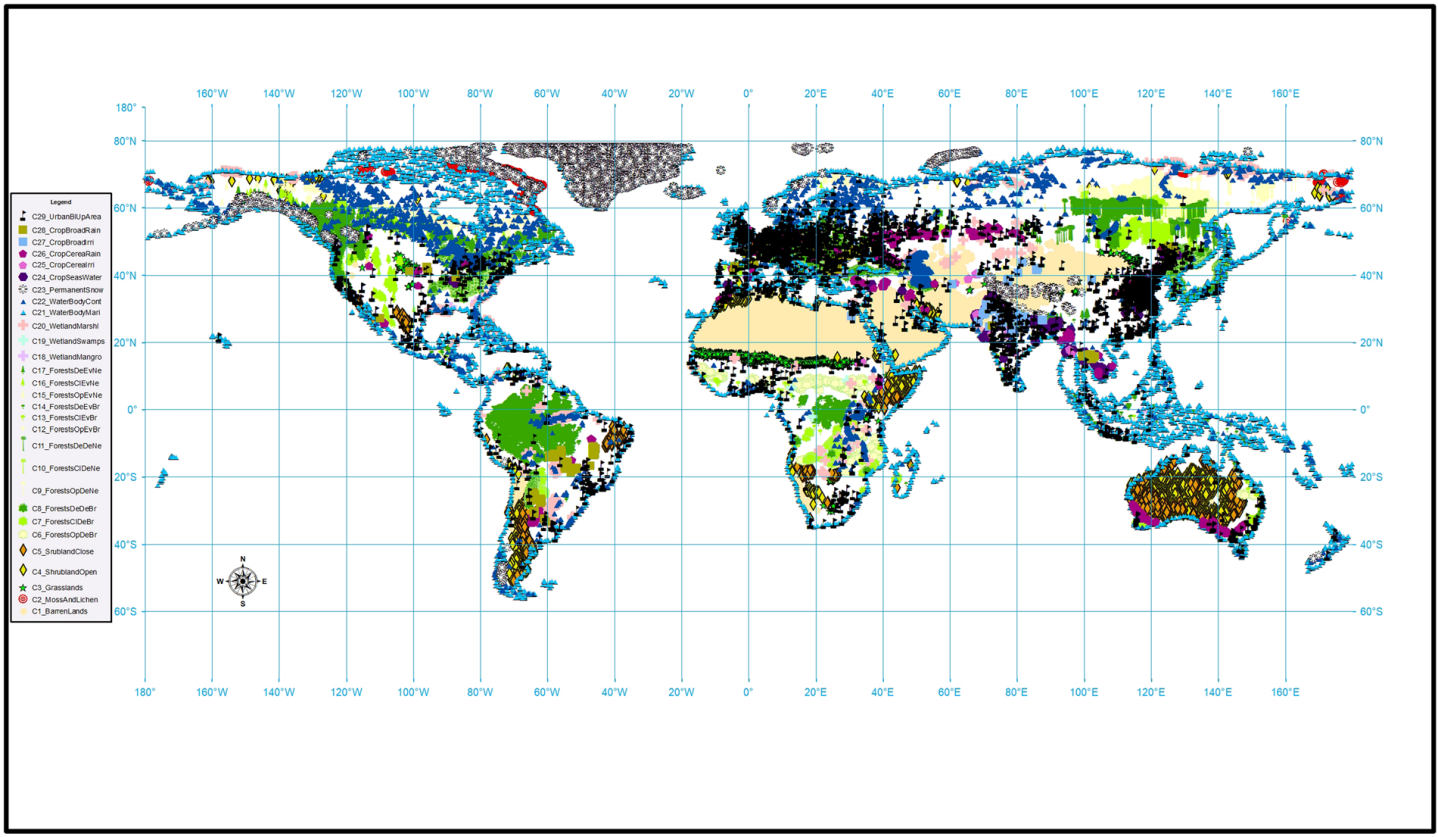
Global map of the distribution of the 2240 × 2240 m tiles representing 29 Land-Use and Land-Cover (LULC) classes that were generated from the spatio-temporal agreement across the 15 global LULC products available in Google Earth Engine. The purity threshold used for each LULC class is specified in Table 6.
- CSV files folder: For user convenience, the metadata of every image tile (i.e., the same information already contained in the image filenames) is also provided in CSV format. Image tiles in the CSV files are organized from the highest to the lowest consensus probability value. These CSV files have 12 columns: ID of LULC Class, Short name of LULC Class, ID Image, Pixel Probability Value as percentage, GHM Value, Center Latitude, Center Longitude, Country Code, Administative Departement Level 1, Administative Departement Level 2, Locality, Number of S2 images which represent the number of found instances in the corresponding Sentinel-2 image collection between June 2015 and October 2020, when aggregated and exported as a final image.

For too large LULC classes (i.e., with more than 14000 potential image tiles) that had to undergo the distance-based selection, we provide the user with 2 CSV files: one containing all pure tiles coordinates without geo-referencing columns, and another file containing just the 14000 exported tiles coordinates with their geo-referencing information and metadata.

## Technical Validation

**Fig. 7:**
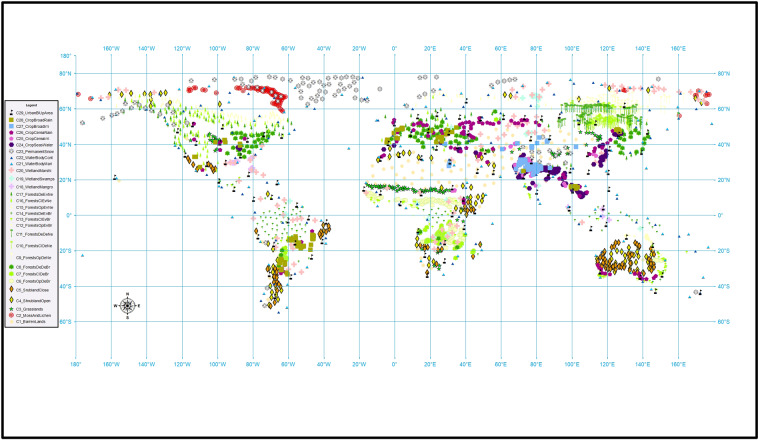
Global distribution of the selected 100 images for each Land-Use and Land-Cover (LULC) class to perform the validation of the 29 LULC classes contained in the Sentinel2GlobalLULC dataset. An add-hoc script in R was used to maximize the geographical distance among the 100 points of each class.

To provide an independent assessment of the quality of the obtained automatic annotation, two of our co-authors who are experts in vegetation mapping have visually inspected a geographically representative sample of 2900 images from the dataset (100 images per class) selected by an algorithm that maximizes the geographical distance between the selected image tiles. This visual inspection was elaborated using very high resolution imagery from both Google Earth and Bing Maps as ground truth. The validation process was established in three stages: First, for each LULC class, we selected 100 image tiles to visually verify their LULC annotation. To maximize the global representativeness of the validated image tiles, their selection was carried out by maximizing the geographical distance among them using an add-hoc script in R. In Fig. 7, we present the distribution map of the 100 image tiles selected for each LULC class. Second, each one of the selected image tiles was visually inspected in Google Earth and Bing Maps by two of the co-authors (E.G. and D.A-S.) to independently assign it to one of the 29 LULC classes. These two experts assigned each image tile to a LULC class when it occupied more than 70% of the image tile. Third, a confusion matrix for this validation was calculated at six different levels of our LULC classification hierarchy (from L0 to L5 as presented in Fig. 2). In Table 8, we summarized the obtained F1 scores at each level.

**Table 8:** Results of the validation procedure of the representativeness of the images contained in the Sentinel2GlobalLULC dataset for each Land-Use and Land-Cover (LULC) class at different levels of the hierarchical legend (from L0 to L5).

L0	F1	L1	F1	L2	F1	L3	F1	L4	F1	L5	F1
Land Cover	0.99	Terrestrial Lands	1.00	BarrenLands	0.97	BarrenLands	0.97	BarrenLands	0.97	C1 BarrenLands	0.97
				MossAndLichen	NA	MossAndLichen	NA	MossAndLichen	NA	C2 MossAndLichen	NA
				Grasslands	0.75	Grasslands	0.75	Grasslands	0.75	C3 Grasslands	0.75
				Shrubland	0.89	ShrublandOpen	0.76	ShrublandOpen	0.76	C4 ShrublandOpen	0.76
						SrublandClose	0.97	SrublandClose	0.97	C5 SrublandClose	0.97
				Forests	1.00	ForestsDe	1.00	ForestsDeBr	1.00	C6 ForestsOpDeBr	0.82
										C7 ForestsClDeBr	0.89
										C8 ForestsDeDeBr	0.96
								ForestsDeNe	1.00	C9 ForestsOpDeNe	0.92
										C10 ForestsClDeNe	0.88
										C11 ForestsDeDeNe	0.95
						ForestsEv	0.99	ForestsEvBr	0.99	C12 ForestsOpEvBr	0.70
										C13 ForestsClEvBr	0.72
										C14 ForestsDeEvBr	0.91
								ForestsEvNe	1.00	C15 ForestsOpEvNe	0.82
										C16 ForestsClEvNe	0.88
										C17 ForestsDeEvNe	0.99
				PermanentSnow	1.00	PermanentSnow	1.00	PermanentSnow	1.00	C23 PermanentSnow	1.00
		Aquatic Lands	0.98	Wetland	0.96	WetlandMangro	0.96	WetlandMangro	0.96	C18 WetlandMangro	0.96
						WetlandSwamps	0.99	WetlandSwamps	0.99	C19 WetlandSwamps	0.99
						WetlandMarshl	0.94	WetlandMarshl	0.94	C20 WetlandMarshl	0.94
				WaterBody	0.99	WaterBodyMari	0.95	WaterBodyMari	0.95	C21 WaterBodyMari	0.95
						WaterBodyCont	0.93	WaterBodyCont	0.93	C22 WaterBodyCont	0.93
Land Use	0.98	Croplands	0.98	CropSeasWater	0.93	CropSeasWater	0.93	CropSeasWater	0.93	C24 CropSeasWater	0.93
				CropCerea	0.99	CropCereaIrri	1.00	CropCereaIrri	1.00	C25 CropCereaIrri	1.00
						CropCereaRain	0.98	CropCereaRain	0.98	C26 CropCereaRain	0.98
				CropBroad	0.99	CropBroadIrri	1.00	CropBroadIrri	1.00	C27 CropBroadIrri	1.00
						CropBroadRain	0.99	CropBroadRain	0.99	C28 CropBroadRain	0.99
		UrbanBlUpArea	0.99	UrbanBlUpArea	0.99	UrbanBlUpArea	0.99	UrbanBlUpArea	0.99	C29 UrbanBlUpArea	0.99
Mean	0.99		0.98		0.95		0.95		0.95		0.91

Accuracy is expressed as the mean F1 score (i.e., a balance between precision and recall) for each LULC class at each level, rounded to two decimal values.

The obtained mean F1 scores ranged from 0.99 at level L0 to 0.91 at level L5 (Table 8). Such decrease in accuracy as the number of classes increased from level L0 to level L5 was mainly due to the hard distinction for the human eye between forest types at L5 and to the visual features complexity in Grasslands and Shrublands classes from level L2.

## Usage Notes

To make the Sentinel2GlobalLULC^40^ dataset easier to use, reproduce, and exploit and to promote its usage for DL models training, we have provided users with a python code to load all RGB images and train several Convolutional Neural Networks (CNNs) models on them using different learning hyper-parameters. These CNNs can only be trained on Sentinel2GlobalLULC to classify scene images into one of 29 LULC types. Knowing that most CNN frameworks admit only jpeg or png image formats, we provide a python script to convert “.tif” into “.jpeg” format with a full control on the conversion quality. Moreover, since for some LULC classes we limited the number of exported images to 14000, we provide a python script that can help the user to export more Sentinel-2 images and bands of each class if needed, using the coordinates stored in the CSV files.

In addition, to provide a global insight about the consistency and accuracy of the global distribution of these 29 LULC classes, we also publicly share their final reprojected global consensus maps as GEE assets. To assist the user in visualizing the global distribution of each LULC class, we have provide a GEE script with the LULC assets links to import, manipulate, and visualize. Further image exportation is also possible through GEE python API and we gave the user a complete control on the number of tiles to export, the time interval to select for image collections, the cloud removal parameters, the true RGB colors calibration values, and the Google drive account where to store the exported images. The user should be aware that GEE currently imposes a limited request number with a maximum of 3000 exportation tasks to run simultaneously on the same Google account.

## Limitations

In this section, we highlight the limitations of Sentinel2GlobalLULC^40^ dataset, its suitable DL setting and new perspectives of its usage.

Sentinel2GlobalLULC is specifically designed for scene image classification, so each image was annotated with one LULC class at scene level, not at pixel level. That is, it does not contain mixed classes, such as mixed forests (e.g. where both evergreen and deciduous trees coexist) or mosaics of croplands and natural vegetation, and it does not allow to identify polygons of different classes within an image scene.

Another point that the user should take into consideration is that some LULC classes have an inherently restricted geographical distribution since they only occur in particular environmental conditions of the world (e.g. Mangroves, Swamps, Seasonally flooded croplands, etc.). For these naturally restricted classes, one can not expect to find a broad geographical distribution of the training image tiles in our dataset. Other LULC classes (e.g. different types of forests, shrublands or grasslands, barren lands, etc…) are more widely distributed around the world. However, there exist conceptual and methodological differences across current LULC products on the definition of each class and used methods to map them. As a result of these inconsistencies, for widely distributed classes, one can not expect either to find a continuous geographical distribution of the training image tiles in our dataset. On the one hand, annotation quality of the training dataset is critical to get accurate models and it constitutes the one of the main challenges for the users^49^. Our approach to maximize the annotation quality was done via consensus across multiple LULC products over the world. On the other hand, a wide representativeness in the training dataset under different environmental conditions per class around the globe is preferred to provide transferability of the model to the widest set of existing geographical locations of each class around the world. Hence, to find a trade-off in our dataset between a wide representativeness across the world for each class while maintaining a high annotation quality, we decreased the threshold for spatial purity up to 75% in some classes. As a result, we provided a larger number of image tiles per class which are geographically distributed around the world in the best way possible. Deep learning models are known to be robust and generalizable in scene classification problems when the training images contain a dominant part of the target class (i.e. the annotated class occupies more than 60% of all pixels in the scene)^42^. Geographical transferability of DL classification models is known to be high, i.e., models trained with images from one geographical location maintain high classification accuracy when applied to very distant geographical locations^50^. In addition, it is known that models trained only with a limited part of a data distribution actually reach similar test error than models trained on the complete data distribution^51^. However, the inherent under-representation of some LULC classes and regions remains a limitation of our dataset, especially in disagreement areas. In addition, the inter-regions variation in terms of spatial patterns within the same LULC class (e.g. croplands in central Europe versus croplands in Subsaharan Africa) could constitute a serious limitation to geographical transferability. Thus, additional data and further analysis of DL performance could be required to help these models reach and maintain the same classification performance in every LULC class and region of the world. To give Sentinel2GlobalLULC users a clear information about the geographic representativeness in the 29 LULC classes, we included in the same repository with the dataset, a compressed file called “Geographic_Representativeness” that contains a csv file for each LULC class with the complete list of countries represented in that class. Each csv file has two columns, the first one gives the country code and the second one gives the number of images provided in that country for that LULC class. In addition to these 29 csv files, we provided another csv file that maps each ISO Alpha-2 country code to its original full country name.

The spatial resolution of the images in our dataset is that of Sentinel-2 RGB bands, i.e. 10 m/pixel, and the annotation is organized in image tiles of 2240 × 2240 m. Hence, this dataset is conceived to build models that use image tiles around 2240 × 2240 m at a spatial resolution of 10 m/pixel. As a result, the output LULC map produced by these models will have a native spatial resolution of 2240 × 2240 m. To overcome this spatial resolution limitation, Image super-resolution (SR) techniques could be of great utility. SR techniques improve various remote sensing applications by allowing users to create finer spatial details than those captured by the original acquisition sensors, and have shown to be very effective in this application^52^. Thus, a very promising solution for this limitation would be to artificially fine-tune Sentinel2GlobalLULC images resolution using SR as a preprocessing strategy before the training step and to offer more flexibility regarding the spatial resolution at the global mapping step.

Deep learning CNNs are usually trained only with the RGB channels available in each image. Thus, our dataset contains only RBG images. Nevertheless, multi-input CNNs nowadays are effectively combining information provided by different remote sensing sources at different scales and with various data types^53^. To give Sentinel2GlobalLULC users a possibility to take advantage from these multi-input models, we provided in the shared Github code^54^ (10.5281/zenodo.5638409) of our dataset, a data exportation script with a full control on the satellite source to choose (e.g. Sentinel-1..etc) and the spectral bands (e.g NIR, NDVI..etc) they want to export from these satellites.

**Fig. 8:**
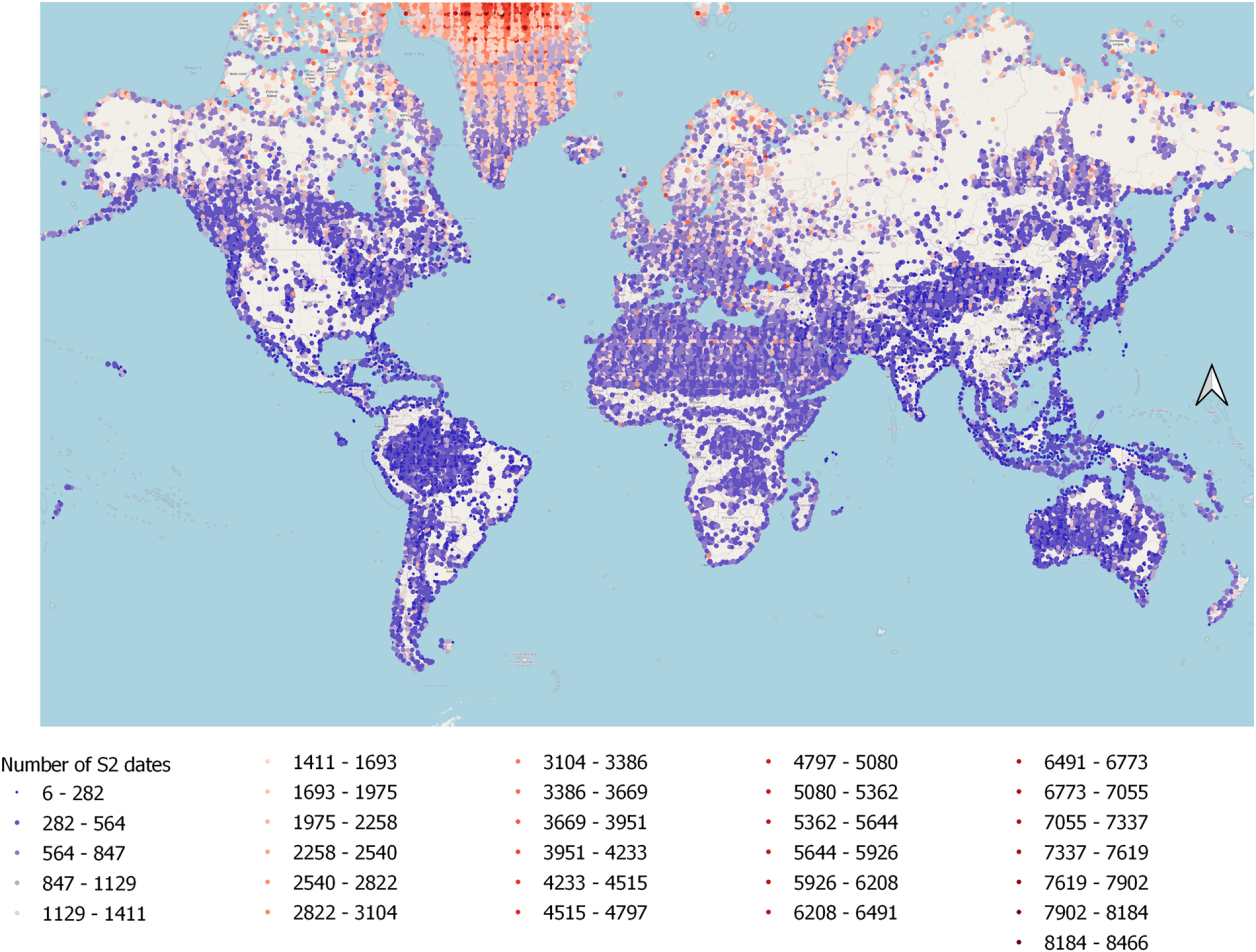
The number of Sentinel-2 dates used to build each image composite in Sentinel2GlobalLULC dataset. This number is represented under different intervals (An individual map for each one of the 29 LULC classes is presented in Supplementary File 4).

Another important point that the user should take into consideration is that to build each image in our dataset, we combined Sentinel-2 images that were acquired at all available dates in the corresponding image collection between June 2015 and October 2020. Thus, each image is built from a different number of images since image collections in most locations of the northern hemisphere contains more than those situated in the southern hemisphere. To highlight this difference between both parts of the planet, we present in Fig. 8, the number of Sentinel-2 images (dates) used to build each image tile in the world. In addition, we give in Supplementary File 4, 29 figures similar to Fig. 8, but this time each one represent this number of collected Sentinel-2 dates for a different LULC class (from C1 to C29). Furthermore, we added to the 29 CSV files of Sentinel2GlobalLULC dataset, a new column representing the number of Sentinel-2 aggregated images to composite each exported image (this column is called “Number of S2 images”).

The user should be aware that our 25th-percentile composite method was realized on each one of the three reflectance bands (R, G and B) independently, which means that their 25th percentile could have been selected from different dates from June 2015 to October 2022. Despite applying the median independently on each band is a frequent method for compositing time-series of Landsat and Sentinel-2 imagery (e.g.^46,48^), we used the 25th percentile independently on each band since it is more conservative to remove clouds and other atmospheric noise in very cloudy regions^44,45,47^. In addition, compositing each band independently was motivated by computational resources limitations in GEE, since extracting the overall 25-percentile of all these 10 m resolution bands combined was more prone to lead to out of memory time-out errors.

Despite these limitations, Sentinel2GlobalLULC remains to our knowledge the first global LULC mapping dataset that includes up to 29 LULC classes, a number much higher than the valuable Dynamic World dataset^55^, which only provides 9 LULC classes yet.

## Supplementary information

Supplementary File 1

Supplementary File 2

Supplementary File 3

Supplementary File 4

## Data Availability

All used scripts to implement or use our dataset and links to the GEE stored assets are available in the following Github repository^54^ (10.5281/zenodo.5638409) repository with guidelines stored in a README file explaining all instructions about their execution.
