# Supplementary material for: Sentinel2GlobalLULC: A Sentinel-2 RGB image tile dataset for global land use/cover mapping with deep learning
Source: Sci Data. 2022 Nov 9;9:681. doi: 10.1038/s41597-022-01775-8 (PMC9646844; doi:10.1038/s41597-022-01775-8)
Supplement: Supplementary file 1 [file 41597_2022_1775_MOESM1_ESM.pdf]

Supplementary File 1

Table 1. Legends mapping between Sentinel2GlobalLULC classification system and FAO's Land Cover Classification System (LCCS).

FAO's LCCS System			Sentinel2GlobalLULC
Dichotomous phase 1	Dichotomous phase 2	Dichotomous phase 3	
A. Primarily vegetated	A1. Terrestrial	A11.Cultivated and Managed Terrestrial Areas.	C25 Irrigated cereal croplands
			C26 Rainfed cereal croplands
			C27 Irrigated broadleaf croplands
			C28 Rainfed broadleaf croplands
		A12.Natural and Semi-Natural Terrestrial Vegetation.	C2 MossAndLichen
			C3 Grasslands
			C4 ShrublandOpen
			C5 SrublandClose
			C6 ForestsOpDeBr
			C7 ForestsClDeBr
			C8 ForestsDeDeBr
			C9 ForestsOpDeNe
			C10 ForestsClDeNe
			C11 ForestsDeDeNe
			C12 ForestsOpEvBr
			C13 ForestsClEvBr
			C14 ForestsDeEvBr
			C15 ForestsOpEvNe
			C16 ForestsClEvNe
			C17 ForestsDeEvNe
	A2. Aquatic or regularly flooded	A23.Cultivated Aquatic or Regularly Flooded Areas.	C24 CropSeasWater
		A24.Natural and Semi-Natural Aquatic or Regularly Flooded Vegetation.	C18 WetlandMangro
			C19 WetlandSwamps
			C20 WetlandMarshl
B. Primarily non-vegetated	B1. Terrestrial	B15.Artificial Surfaces and Associated Areas.	
		B16.Bare Areas.	C1 BarrenLands
	B2. Aquatic or regularly flooded	B27.Artificial Water Bodies, Snow and Ice.	
		B28.Natural Water Bodies, Snow and Ice.	C21 WaterBodyMari
			C22 WaterBodyCont
			C23 PermanentSnow
