# Supplementary material for: Sentinel2GlobalLULC: A Sentinel-2 RGB image tile dataset for global land use/cover mapping with deep learning
Source: Sci Data. 2022 Nov 9;9:681. doi: 10.1038/s41597-022-01775-8 (PMC9646844; doi:10.1038/s41597-022-01775-8)
Supplement: Supplementary file 2 [file 41597_2022_1775_MOESM2_ESM.pdf]

Supplementary File 2

Table 1. Detailed description of criteria used in each product(P1 to P15) to build each LULC class (C1 to C29) in Sentinel2GlobalLULC dataset. NU: Not Used. NA: Not Available.

Class	Product	Description
**C1 BarrenLands**	P1 MCD12Q1.006 MODIS(IGBP)	16. Barren: at least 60% of area is non-vegetated barren (sand, rock, soil) areas with less than 10% vegetation.
	P2 MCD12Q1.006 MODIS(UMD)	15. Non-Vegetated Lands: at least 60% of area is non-vegetated barren (sand, rock, soil) or permanent snow and ice with less than 10% vegetation.
	P3 MCD12Q1.006 MODIS(LAI)	NA
	P4 MCD12Q1.006 MODIS(BGC))	7. Non-Vegetated Lands: at least 60% of area is non-vegetated barren (sand, rock, soil) or permanent snow/ice with less than 10% vegetation.
	P5 MCD12Q1.006 MODIS(PFT))	11. Non-Vegetated Lands: at least 60% of area is non-vegetated barren (sand, rock, soil) with less than 10% vegetation.
	P6 CGLS-LC100 collection 3	60. Bare / sparse vegetation. Lands with exposed soil, sand, or rocks and never has more than 10 % vegetated cover during any time of the year.
	P7 Global Forest Cover Change (GFCC)	Tree canopy cover<10%
	P8 GlobCover	200. Bare areas
	P9 GFSAD1000	0. Non cropland
	P10 Global PALSAR-2/PALSAR(fnf)	2. Non forest
	P11 Hansen Global Forest Change	(Tree cover <10%) AND (gain=0) AND (loss=0) AND ($datamask \neq$ 2. Permanent water bodies)
	P12 Global Forest Canopy Height	Tree heights<1m
	P13 JRC Yearly Water Classification History	(1. Not water) OR (0. No data)
	P14 JRC Global Surface Water Mapping Layers	0. max_extent
	P15 Tsinghua FROM-GLC year of change to impervious surface	Not($\geq$ 1)
**C2 MossAndLichen**	P1 MCD12Q1.006 MODIS(IGBP)	16. Barren: at least 60% of area is non-vegetated barren (sand, rock, soil) areas with less than 10% vegetation.
	P2 MCD12Q1.006 MODIS(UMD)	15. Non-Vegetated Lands: at least 60% of area is non-vegetated barren (sand, rock, soil) or permanent snow and ice with less than 10% vegetation.
	P3 MCD12Q1.006 MODIS(LAI)	NA
	P4 MCD12Q1.006 MODIS(BGC))	7. Non-Vegetated Lands: at least 60% of area is non-vegetated barren (sand, rock, soil) or permanent snow/ice with less than 10% vegetation.
	P5 MCD12Q1.006 MODIS(PFT))	11. Non-Vegetated Lands: at least 60% of area is non-vegetated barren (sand, rock, soil) with less than 10% vegetation.
	P6 CGLS-LC100 collection 3	NA
	P7 Global Forest Cover Change (GFCC)	Tree canopy cover<10%
	P8 GlobCover	(200. Bare areas) OR (150. Sparse (>15%) vegetation (woody vegetation, shrubs, grassland))
	P9 GFSAD1000	0. Non cropland
	P10 Global PALSAR-2/PALSAR(fnf)	2. Non forest
	P11 Hansen Global Forest Change	(Tree cover <10%) AND (gain=0) AND (loss=0) AND ($datamask \neq$ 2. Permanent water bodies)
	P12 Global Forest Canopy Height	Tree heights<1m
	P13 JRC Yearly Water Classification History	(1. Not water) OR (0. No data)
	P14 JRC Global Surface Water Mapping Layers	0. max_extent
	P15 Tsinghua FROM-GLC year of change to impervious surface	Not($\geq$ 1)
**C3 Grasslands**	P1 MCD12Q1.006 MODIS(IGBP)	10. Grasslands: dominated by herbaceous annuals (<2m).
	P2 MCD12Q1.006 MODIS(UMD)	10. Grasslands: dominated by herbaceous annuals (<2m).
	P3 MCD12Q1.006 MODIS(LAI)	1. Grasslands: dominated by herbaceous annuals (<2m) including cereal croplands.
	P4 MCD12Q1.006 MODIS(BGC))	6. Annual Grass Vegetation: dominated by herbaceous annuals (<2m) including cereal croplands.
	P5 MCD12Q1.006 MODIS(PFT))	6. Grass: dominated by herbaceous annuals (<2m) that are not cultivated.
	P6 CGLS-LC100 collection 3	30.Herbaceous vegetation. Plants without persistent stem or shoots above ground and lacking definite firm structure. Tree and shrub cover is less than 10 %.
	P7 Global Forest Cover Change (GFCC)	Tree canopy cover<10%
	P8 GlobCover	140. Closed to open (>15%) grassland
	P9 GFSAD1000	NA
	P10 Global PALSAR-2/PALSAR(fnf)	2. Non forest
	P11 Hansen Global Forest Change	(Tree cover <10%) AND (gain=0) AND (loss=0) AND ($datamask \neq$ 2. Permanent water bodies)
	P12 Global Forest Canopy Height	Tree heights<2m
	P13 JRC Yearly Water Classification History	(1. Not water) OR (0. No data)
	P14 JRC Global Surface Water Mapping Layers	0. max_extent
	P15 Tsinghua FROM-GLC year of change to impervious surface	Not($\geq$ 1)
**C4 ShrublandOpen**	P1 MCD12Q1.006 MODIS(IGBP)	7. Open Shrublands: dominated by woody perennials (1-2m height) 10-60% cover.
	P2 MCD12Q1.006 MODIS(UMD)	7. Open Shrublands: dominated by woody perennials (1-2m height) 10-60% cover.
	P3 MCD12Q1.006 MODIS(LAI)	2. Shrublands: shrub (1-2m) cover >10%.
	P4 MCD12Q1.006 MODIS(BGC))	NA
	P5 MCD12Q1.006 MODIS(PFT))	5. Shrub: Shrub (1-2m) cover >10%.
	P6 CGLS-LC100 collection 3	(20. Shrubs. Woody perennial plants with persistent and woody stems and without any defined main stem being less than 5 m tall. The shrub foliage can be either evergreen or deciduous.) AND (10 <shrub-coverfraction <50)
	P7 Global Forest Cover Change (GFCC)	Tree canopy cover<10%
	P8 GlobCover	150. Sparse (>15%) vegetation (woody vegetation, shrubs, grassland)
	P9 GFSAD1000	0. Non cropland
	P10 Global PALSAR-2/PALSAR(fnf)	2. Non forest
	P11 Hansen Global Forest Change	(Tree cover <10%) AND (gain=0) AND (loss=0) AND ($datamask \neq$ 2. Permanent water bodies)
	P12 Global Forest Canopy Height	Tree heights<2m
	P13 JRC Yearly Water Classification History	(1. Not water) OR (0. No data)
	P14 JRC Global Surface Water Mapping Layers	0. max_extent
	P15 Tsinghua FROM-GLC year of change to impervious surface	Not($\geq$ 1)

**C5 SrublandClose**	P1 MCD12Q1.006 MODIS(IGBP)	6. Closed Shrublands: dominated by woody perennials (1-2m height) >60% cover.
	P2 MCD12Q1.006 MODIS(UMD)	6. Closed Shrublands: dominated by woody perennials (1-2m height) >60% cover.
	P3 MCD12Q1.006 MODIS(LAI)	2. Shrublands: shrub (1-2m) cover >10%.
	P4 MCD12Q1.006 MODIS(BGC))	NA
	P5 MCD12Q1.006 MODIS(PFT))	5. Shrub: Shrub (1-2m) cover >10%.
	P6 CGLS-LC100 collection 3	(20. Shrubs. Woody perennial plants with persistent and woody stems and without any defined main stem being less than 5 m tall. The shrub foliage can be either evergreen or deciduous.) AND (shrub-coverfraction >50)
	P7 Global Forest Cover Change (GFCC)	Tree canopy cover<10%
	P8 GlobCover	130. Closed to open (>15%) shrubland (<5m)
	P9 GFSAD1000	0. Non cropland
	P10 Global PALSAR-2/PALSAR(fnf)	2. Non forest
	P11 Hansen Global Forest Change	(Tree cover <10%) AND (gain=0) AND (loss=0) AND ($datamask \neq 2$. Permanent water bodies)
	P12 Global Forest Canopy Height	Tree heights<2m
	P13 JRC Yearly Water Classification History	(1. Not water) OR (0. No data)
	P14 JRC Global Surface Water Mapping Layers	0. max_extent
	P15 Tsinghua FROM-GLC year of change to impervious surface	Not($\geq$ 1)
**C6 ForestsOpDeBr**	P1 MCD12Q1.006 MODIS(IGBP)	NA
	P2 MCD12Q1.006 MODIS(UMD)	NA
	P3 MCD12Q1.006 MODIS(LAI)	NA
	P4 MCD12Q1.006 MODIS(BGC))	4. Deciduous Broadleaf Vegetation: dominated by deciduous broadleaf trees and shrubs (>1m). Woody vegetation cover >10%.
	P5 MCD12Q1.006 MODIS(PFT))	4. Deciduous Broadleaf Trees: dominated by deciduous broadleaf trees (>2m). Tree cover >10%.
	P6 CGLS-LC100 collection 3	(4. Deciduous broad leaf) ADD (15% <tree-coverfraction <30%)
	P7 Global Forest Cover Change (GFCC)	15%<Tree canopy cover<30%
	P8 GlobCover	60. Open (15-40%) broadleaved deciduous forest (>5m)
	P9 GFSAD1000	NA
	P10 Global PALSAR-2/PALSAR(fnf)	1. Forest
	P11 Hansen Global Forest Change	(15<Tree cover <30%) AND (gain=0) AND (loss=0) AND ($datamask \neq 2$. Permanent water bodies)
	P12 Global Forest Canopy Height	Tree heights>2m
	P13 JRC Yearly Water Classification History	(1. Not water) OR (0. No data)
	P14 JRC Global Surface Water Mapping Layers	0. max_extent
	P15 Tsinghua FROM-GLC year of change to impervious surface	Not($\geq$ 1)
**C7 ForestsClDeBr**	P1 MCD12Q1.006 MODIS(IGBP)	NA
	P2 MCD12Q1.006 MODIS(UMD)	NA
	P3 MCD12Q1.006 MODIS(LAI)	NA
	P4 MCD12Q1.006 MODIS(BGC))	4. Deciduous Broadleaf Vegetation: dominated by deciduous broadleaf trees and shrubs (>1m). Woody vegetation cover >10%.
	P5 MCD12Q1.006 MODIS(PFT))	4. Deciduous Broadleaf Trees: dominated by deciduous broadleaf trees (>2m). Tree cover >10%.
	P6 CGLS-LC100 collection 3	(4. Deciduous broad leaf) ADD (40% <tree-coverfraction <60%)
	P7 Global Forest Cover Change (GFCC)	40% <Tree canopy cover <60%
	P8 GlobCover	50. Closed (>40%) broadleaved deciduous forest (>5m)
	P9 GFSAD1000	NA
	P10 Global PALSAR-2/PALSAR(fnf)	1. Forest
	P11 Hansen Global Forest Change	(40 <Tree cover <60%) AND (gain=0) AND (loss=0) AND ($datamask \neq 2$. Permanent water bodies)
	P12 Global Forest Canopy Height	Tree heights >2m
	P13 JRC Yearly Water Classification History	(1. Not water) OR (0. No data)
	P14 JRC Global Surface Water Mapping Layers	0. max_extent
	P15 Tsinghua FROM-GLC year of change to impervious surface	Not($\geq$ 1)
**C8 ForestsDeDeBr**	P1 MCD12Q1.006 MODIS(IGBP)	4. Deciduous Broadleaf Forests: dominated by deciduous broadleaf trees (canopy >2m). Tree cover >60%.
	P2 MCD12Q1.006 MODIS(UMD)	4. Deciduous Broadleaf Forests: dominated by deciduous broadleaf trees (canopy >2m). Tree cover >60%.
	P3 MCD12Q1.006 MODIS(LAI)	6. Deciduous Broadleaf Forests: dominated by deciduous broadleaf trees (canopy >2m). Tree cover >60%.
	P4 MCD12Q1.006 MODIS(BGC))	4. Deciduous Broadleaf Vegetation: dominated by deciduous broadleaf trees and shrubs (>1m). Woody vegetation cover >10%.
	P5 MCD12Q1.006 MODIS(PFT))	4. Deciduous Broadleaf Trees: dominated by deciduous broadleaf trees (>2m). Tree cover >10%.
	P6 CGLS-LC100 collection 3	(4. Deciduous broad leaf)ADD (tree-coverfraction >60%)
	P7 Global Forest Cover Change (GFCC)	Tree canopy cover>60%
	P8 GlobCover	50. Closed (>40%) broadleaved deciduous forest (>5m).
	P9 GFSAD1000	NA
	P10 Global PALSAR-2/PALSAR(fnf)	1. Forest
	P11 Hansen Global Forest Change	(Tree cover >60%) AND (gain=0) AND (loss=0) AND ($datamask \neq 2$. Permanent water bodies)
	P12 Global Forest Canopy Height	Tree heights>2m
	P13 JRC Yearly Water Classification History	(1. Not water) OR (0. No data)
	P14 JRC Global Surface Water Mapping Layers	0. max_extent
	P15 Tsinghua FROM-GLC year of change to impervious surface	Not($\geq$ 1)
**C9 ForestsOpDeNe**	P1 MCD12Q1.006 MODIS(IGBP)	NA
	P2 MCD12Q1.006 MODIS(UMD)	NA
	P3 MCD12Q1.006 MODIS(LAI)	NA
	P4 MCD12Q1.006 MODIS(BGC))	3. Deciduous Needleleaf Vegetation: dominated by deciduous needleleaf (larch) trees and shrubs. (>1m). Woody vegetation cover >10%.
	P5 MCD12Q1.006 MODIS(PFT))	3.Deciduous Needleleaf Trees: dominated by deciduous needleleaf (larch) trees (>2m). Tree cover >10%.
	P6 CGLS-LC100 collection 3	(3.Deciduous needle leaf) ADD (15% <tree-coverfraction <30%)
	P7 Global Forest Cover Change (GFCC)	15%<Tree canopy cover<30%
	P8 GlobCover	NA
	P9 GFSAD1000	NA
	P10 Global PALSAR-2/PALSAR(fnf)	1.Forest
	P11 Hansen Global Forest Change	(15<Tree cover <30%) AND (gain=0) AND (loss=0) AND ($datamask \neq 2$. Permanent water bodies)
	P12 Global Forest Canopy Height	Tree heights>2m
	P13 JRC Yearly Water Classification History	(1. Not water) OR (0. No data)
	P14 JRC Global Surface Water Mapping Layers	0. max_extent
	P15 Tsinghua FROM-GLC year of change to impervious surface	Not($\geq$ 1)
**C10 ForestsClDeNe**	P1 MCD12Q1.006 MODIS(IGBP)	NA
	P2 MCD12Q1.006 MODIS(UMD)	NA
	P3 MCD12Q1.006 MODIS(LAI)	NA
	P4 MCD12Q1.006 MODIS(BGC))	3. Deciduous Needleleaf Vegetation: dominated by deciduous needleleaf (larch) trees and shrubs. (>1m). Woody vegetation cover >10%.
	P5 MCD12Q1.006 MODIS(PFT))	3.Deciduous Needleleaf Trees: dominated by deciduous needleleaf (larch) trees (>2m). Tree cover >10%.
	P6 CGLS-LC100 collection 3	(3.Deciduous needle leaf) ADD (40% <tree-coverfraction <60%)
	P7 Global Forest Cover Change (GFCC)	40%<Tree canopy cover<60%
	P8 GlobCover	NA
	P9 GFSAD1000	NA
	P10 Global PALSAR-2/PALSAR(fnf)	1. Forest
	P11 Hansen Global Forest Change	(40<Tree cover <60%) AND (gain=0) AND (loss=0) AND ($datamask \neq 2$. Permanent water bodies)
	P12 Global Forest Canopy Height	Tree heights>2m
	P13 JRC Yearly Water Classification History	(1. Not water) OR (0. No data)
	P14 JRC Global Surface Water Mapping Layers	0. max_extent
	P15 Tsinghua FROM-GLC year of change to impervious surface	Not($\geq$ 1)

**C11 ForestsDeDeNe**	P1 MCD12Q1.006 MODIS(IGBP)	3. Deciduous Needleleaf Forests: dominated by deciduous needleleaf (larch) trees (canopy >2m). Tree cover >60%.
	P2 MCD12Q1.006 MODIS(UMD)	3. Deciduous Needleleaf Forests: dominated by deciduous needleleaf (larch) trees (canopy >2m). Tree cover >60%.
	P3 MCD12Q1.006 MODIS(LAI)	8. Deciduous Needleleaf Forests: dominated by deciduous needleleaf (larch) trees (canopy >2m). Tree cover >60%.
	P4 MCD12Q1.006 MODIS(BGC))	3. Deciduous Needleleaf Vegetation: dominated by deciduous needleleaf (larch) trees and shrubs (>1m).<br>Woody vegetation cover >10%.
	P5 MCD12Q1.006 MODIS(PFT))	3. Deciduous Needleleaf Trees: dominated by deciduous needleleaf (larch) trees (>2m). Tree cover >10%.
	P6 CGLS-LC100 collection 3	(3.Deciduous needle leaf) ADD (tree-coverfraction >60%)
	P7 Global Forest Cover Change (GFCC)	Tree canopy cover>60%
	P8 GlobCover	NA
	P9 GFSAD1000	NA
	P10 Global PALSAR-2/PALSAR(fnf)	1. Forest
	P11 Hansen Global Forest Change	(Tree cover >60%) AND (gain=0) AND (loss=0) AND ($datamask \neq 2$. Permanent water bodies)
	P12 Global Forest Canopy Height	Tree heights>2m
	P13 JRC Yearly Water Classification History	(1. Not water) OR (0. No data)
	P14 JRC Global Surface Water Mapping Layers	0. max_extent
	P15 Tsinghua FROM-GLC year of change to impervious surface	Not(≥ 1)
**C12 ForestsOpEvBr**	P1 MCD12Q1.006 MODIS(IGBP)	NA
	P2 MCD12Q1.006 MODIS(UMD)	NA
	P3 MCD12Q1.006 MODIS(LAI)	NA
	P4 MCD12Q1.006 MODIS(BGC))	2. Evergreen Broadleaf Vegetation: dominated by evergreen broadleaf and palmate trees and shrubs (>1m).<br>Woody vegetation cover >10%.
	P5 MCD12Q1.006 MODIS(PFT))	2. Evergreen Broadleaf Trees: dominated by evergreen broadleaf and palmate trees (>2m). Tree cover >10%.
	P6 CGLS-LC100 collection 3	(2.Evergreen broad leaf) ADD (15<tree-coverfraction <30%)
	P7 Global Forest Cover Change (GFCC)	15%<Tree canopy cover<30%
	P8 GlobCover	40.Closed to open (>15%) broadleaved evergreen and/or semi-deciduous forest (>5m)
	P9 GFSAD1000	NA
	P10 Global PALSAR-2/PALSAR(fnf)	1. Forest
	P11 Hansen Global Forest Change	(15<Tree cover <30%) AND (gain=0) AND (loss=0) AND ($datamask \neq 2$. Permanent water bodies)
	P12 Global Forest Canopy Height	Tree heights>2m
	P13 JRC Yearly Water Classification History	(1. Not water) OR (0. No data)
	P14 JRC Global Surface Water Mapping Layers	0. max_extent
	P15 Tsinghua FROM-GLC year of change to impervious surface	Not(≥ 1)
**C13 ForestsClEvBr**	P1 MCD12Q1.006 MODIS(IGBP)	NA
	P2 MCD12Q1.006 MODIS(UMD)	NA
	P3 MCD12Q1.006 MODIS(LAI)	NA
	P4 MCD12Q1.006 MODIS(BGC))	2. Evergreen Broadleaf Vegetation: dominated by evergreen broadleaf and palmate trees and shrubs (>1m).<br>Woody vegetation cover >10%.
	P5 MCD12Q1.006 MODIS(PFT))	2. Evergreen Broadleaf Trees: dominated by evergreen broadleaf and palmate trees (>2m). Tree cover >10%.
	P6 CGLS-LC100 collection 3	(2.Evergreen broad leaf) ADD (40<tree-coverfraction <60%)
	P7 Global Forest Cover Change (GFCC)	40%<Tree canopy cover<60%
	P8 GlobCover	40.Closed to open (>15%) broadleaved evergreen and/or semi-deciduous forest (>5m)
	P9 GFSAD1000	NA
	P10 Global PALSAR-2/PALSAR(fnf)	1. Forest
	P11 Hansen Global Forest Change	(40<Tree cover <60%) AND (gain=0) AND (loss=0) AND ($datamask \neq 2$. Permanent water bodies)
	P12 Global Forest Canopy Height	Tree heights>2m
	P13 JRC Yearly Water Classification History	(1. Not water) OR (0. No data)
	P14 JRC Global Surface Water Mapping Layers	0. max_extent
	P15 Tsinghua FROM-GLC year of change to impervious surface	Not(≥ 1)
**C14 ForestsDeEvBr**	P1 MCD12Q1.006 MODIS(IGBP)	2. Evergreen Broadleaf Forests: dominated by evergreen broadleaf and palmate trees (canopy >2m). Tree cover >60%.
	P2 MCD12Q1.006 MODIS(UMD)	2. Evergreen Broadleaf Forests: dominated by evergreen broadleaf and palmate trees (canopy >2m). Tree cover >60%.
	P3 MCD12Q1.006 MODIS(LAI)	5. Evergreen Broadleaf Forests: dominated by evergreen broadleaf and palmate trees (canopy >2m). Tree cover >60%.
	P4 MCD12Q1.006 MODIS(BGC))	2. Evergreen Broadleaf Vegetation: dominated by evergreen broadleaf and palmate trees and shrubs (>1m).<br>Woody vegetation cover >10%.
	P5 MCD12Q1.006 MODIS(PFT))	2. Evergreen Broadleaf Trees: dominated by evergreen broadleaf and palmate trees (>2m). Tree cover >10%.
	P6 CGLS-LC100 collection 3	(2.Evergreen broad leaf) ADD (tree-coverfraction >60%)
	P7 Global Forest Cover Change (GFCC)	Tree canopy cover>60%
	P8 GlobCover	40.Closed to open (>15%) broadleaved evergreen and/or semi-deciduous forest (>5m)
	P9 GFSAD1000	NA
	P10 Global PALSAR-2/PALSAR(fnf)	1. Forest
	P11 Hansen Global Forest Change	(Tree cover >60%) AND (gain=0) AND (loss=0) AND ($datamask \neq 2$. Permanent water bodies)
	P12 Global Forest Canopy Height	Tree heights>2m
	P13 JRC Yearly Water Classification History	(1. Not water) OR (0. No data)
	P14 JRC Global Surface Water Mapping Layers	0. max_extent
	P15 Tsinghua FROM-GLC year of change to impervious surface	Not(≥ 1)
**C15 ForestsOpEvNe**	P1 MCD12Q1.006 MODIS(IGBP)	9. Savannas: tree cover 10-30% (canopy >2m).
	P2 MCD12Q1.006 MODIS(UMD)	9. Savannas: tree cover 10-30% (canopy >2m).
	P3 MCD12Q1.006 MODIS(LAI)	NA
	P4 MCD12Q1.006 MODIS(BGC))	1. Evergreen Needleleaf Vegetation: dominated by evergreen conifer trees and shrubs (>1m).<br>Woody vegetation cover >10%.
	P5 MCD12Q1.006 MODIS(PFT))	1. Evergreen Needleleaf Trees: dominated by evergreen conifer trees (>2m). Tree cover >10%.
	P6 CGLS-LC100 collection 3	(1. Evergreen needle leaf) ADD (15%<tree-coverfraction<30%)
	P7 Global Forest Cover Change (GFCC)	15%<Tree canopy cover<30%
	P8 GlobCover	90. Open (15-40%) needleleaved deciduous or evergreen forest (>5m)
	P9 GFSAD1000	NA
	P10 Global PALSAR-2/PALSAR(fnf)	1. Forest
	P11 Hansen Global Forest Change	(15%<Tree cover<30%) AND (gain=0) AND (loss=0) AND ($datamask \neq 2$. Permanent water bodies)
	P12 Global Forest Canopy Height	Tree heights>2m
	P13 JRC Yearly Water Classification History	(1. Not water) OR (0. No data)
	P14 JRC Global Surface Water Mapping Layers	0. max_extent
	P15 Tsinghua FROM-GLC year of change to impervious surface	Not(≥ 1)
**C16 ForestsClEvNe**	P1 MCD12Q1.006 MODIS(IGBP)	8. Woody Savannas: tree cover 30-60% (canopy >2m).
	P2 MCD12Q1.006 MODIS(UMD)	8. Woody Savannas: tree cover 30-60% (canopy >2m).
	P3 MCD12Q1.006 MODIS(LAI)	4. Savannas: between 10-60% tree cover (>2m).
	P4 MCD12Q1.006 MODIS(BGC))	1. Evergreen Needleleaf Vegetation: dominated by evergreen conifer trees and shrubs (>1m).<br>Woody vegetation cover >10%.
	P5 MCD12Q1.006 MODIS(PFT))	1. Evergreen Needleleaf Trees: dominated by evergreen conifer trees (>2m). Tree cover >10%.
	P6 CGLS-LC100 collection 3	(1. Evergreen needle leaf) ADD (40%<tree-coverfraction<60%)
	P7 Global Forest Cover Change (GFCC)	40%<Tree canopy cover<60%
	P8 GlobCover	70. Closed (>40%) needleleaved evergreen forest (>5m)
	P9 GFSAD1000	NA
	P10 Global PALSAR-2/PALSAR(fnf)	1. Forest
	P11 Hansen Global Forest Change	(40%<Tree cover<60%) AND (gain=0) AND (loss=0) AND ($datamask \neq 2$. Permanent water bodies)
	P12 Global Forest Canopy Height	Tree heights>2m
	P13 JRC Yearly Water Classification History	(1. Not water) OR (0. No data)
	P14 JRC Global Surface Water Mapping Layers	0. max_extent
	P15 Tsinghua FROM-GLC year of change to impervious surface	Not(≥ 1)

**C17 ForestsDeEvNe**	P1 MCD12Q1.006 MODIS(IGBP)	1. Evergreen Needleleaf Forests: dominated by evergreen conifer trees (canopy >2m). Tree cover >60%.
	P2 MCD12Q1.006 MODIS(UMD)	1. Evergreen Needleleaf Forests: dominated by evergreen conifer trees (canopy >2m). Tree cover >60%.
	P3 MCD12Q1.006 MODIS(LAI)	7. Evergreen Needleleaf Forests: dominated by evergreen conifer trees (canopy >2m). Tree cover >60%.
	P4 MCD12Q1.006 MODIS(BGC))	1. Evergreen Needleleaf Vegetation: dominated by evergreen conifer trees and shrubs (>1m).<br>Woody vegetation cover >10%.
	P5 MCD12Q1.006 MODIS(PFT))	1. Evergreen Needleleaf Trees: dominated by evergreen conifer trees (>2m). Tree cover >10%.
	P6 CGLS-LC100 collection 3	(1. Evergreen needle leaf) ADD (tree-coverfraction>60%)
	P7 Global Forest Cover Change (GFCC)	Tree canopy cover>60%
	P8 GlobCover	70. Closed (>40%) needleleaved evergreen forest (>5m)
	P9 GFSAD1000	NA
	P10 Global PALSAR-2/PALSAR(fnf)	1. Forest
	P11 Hansen Global Forest Change	(Tree cover>60%) AND (gain=0) AND (loss=0) AND ($datamask \neq$ 2. Permanent water bodies)
	P12 Global Forest Canopy Height	Tree heights>2m
	P13 JRC Yearly Water Classification History	(1. Not water) OR (0. No data)
	P14 JRC Global Surface Water Mapping Layers	0. max_extent
	P15 Tsinghua FROM-GLC year of change to impervious surface	Not($\geq$ 1)
**C18 WetlandMangro**	P1 MCD12Q1.006 MODIS(IGBP)	11. Permanent Wetlands: permanently inundated lands with 30-60% water cover and >10% vegetated cover.
	P2 MCD12Q1.006 MODIS(UMD)	11. Permanent Wetlands: permanently inundated lands with 30-60% water cover and >10% vegetated cover.
	P3 MCD12Q1.006 MODIS(LAI)	NA
	P4 MCD12Q1.006 MODIS(BGC))	NA
	P5 MCD12Q1.006 MODIS(PFT))	NA
	P6 CGLS-LC100 collection 3	90. Herbaceous wetland. Lands with a permanent mixture of water and herbaceous or woody vegetation.<br>The vegetation can be present in either salt, brackish, or fresh water.
	P7 Global Forest Cover Change (GFCC)	Tree canopy cover>10%
	P8 GlobCover	170. Closed (>40%) broadleaved semi-deciduous and/or evergreen forest regularly flooded - saline water
	P9 GFSAD1000	NA
	P10 Global PALSAR-2/PALSAR(fnf)	NA
	P11 Hansen Global Forest Change	(Tree cover>10%) AND (gain=0) AND (loss=0) OR (datamask = 2. Permanent water bodies)
	P12 Global Forest Canopy Height	Tree heights>2m
	P13 JRC Yearly Water Classification History	(2. Seasonal water) OR (3. Permanent water)
	P14 JRC Global Surface Water Mapping Layers	1. max_extent
	P15 Tsinghua FROM-GLC year of change to impervious surface	Not($\geq$ 1)
**C19 WetlandSwamps**	P1 MCD12Q1.006 MODIS(IGBP)	11. Permanent Wetlands: permanently inundated lands with 30-60% water cover and >10% vegetated cover.
	P2 MCD12Q1.006 MODIS(UMD)	11. Permanent Wetlands: permanently inundated lands with 30-60% water cover and >10% vegetated cover.
	P3 MCD12Q1.006 MODIS(LAI)	NA
	P4 MCD12Q1.006 MODIS(BGC))	NA
	P5 MCD12Q1.006 MODIS(PFT))	NA
	P6 CGLS-LC100 collection 3	90. Herbaceous wetland. Lands with a permanent mixture of water and herbaceous or woody vegetation.<br>The vegetation can be present in either salt, brackish, or fresh water.
	P7 Global Forest Cover Change (GFCC)	Tree canopy cover>10%
	CritP8a GlobCover	(160. Closed (>40%) broadleaved forest regularly flooded - Fresh water) OR (180. Closed to open (>15%) vegetation<br>(grassland, shrubland, woody vegetation) on regularly flooded or waterlogged<br>soil - fresh, brackish or saline water)
	CritP8b GlobCover	$\neq$(170. Closed (>40%) broadleaved semi-deciduous and/or evergreen forest regularly flooded - saline water)
	P9 GFSAD1000	NA
	P10 Global PALSAR-2/PALSAR(fnf)	NA
	P11 Hansen Global Forest Change	(Tree cover>10%) AND (gain=0) AND (loss=0) OR (datamask = 2. Permanent water bodies)
	P12 Global Forest Canopy Height	Tree heights>2m
	P13 JRC Yearly Water Classification History	(2. Seasonal water) OR (3. Permanent water)
	P14 JRC Global Surface Water Mapping Layers	1. max_extent
	P15 Tsinghua FROM-GLC year of change to impervious surface	Not($\geq$ 1)
**C20 WetlandMarshl**	P1 MCD12Q1.006 MODIS(IGBP)	11. Permanent Wetlands: permanently inundated lands with 30-60% water cover and >10% vegetated cover.
	P2 MCD12Q1.006 MODIS(UMD)	11. Permanent Wetlands: permanently inundated lands with 30-60% water cover and >10% vegetated cover.
	P3 MCD12Q1.006 MODIS(LAI)	NA
	P4 MCD12Q1.006 MODIS(BGC))	NA
	P5 MCD12Q1.006 MODIS(PFT))	NA
	P6 CGLS-LC100 collection 3	90. Herbaceous wetland. Lands with a permanent mixture of water and herbaceous or woody vegetation.<br>The vegetation can be present in either salt, brackish, or fresh water.
	P7 Global Forest Cover Change (GFCC)	Tree canopy cover<10%
	P8 GlobCover	(160. Closed (>40%) broadleaved forest regularly flooded - Fresh water) OR (170. Closed (>40%) broadleaved semi-deciduous<br>and/or evergreen forest regularly flooded - saline water) OR (180. Closed to open (>15%) vegetation (grassland, shrubland, woody<br>vegetation) on regularly flooded or waterlogged soil - fresh, brackish or saline water)
	P9 GFSAD1000	NA
	P10 Global PALSAR-2/PALSAR(fnf)	NA
	P11 Hansen Global Forest Change	(Tree cover<10%) AND (gain=0) AND (loss=0) OR (datamask = 2. Permanent water bodies)
	P12 Global Forest Canopy Height	Tree heights<2m
	P13 JRC Yearly Water Classification History	(2. Seasonal water) OR (3. Permanent water)
	P14 JRC Global Surface Water Mapping Layers	1. max_extent
	P15 Tsinghua FROM-GLC year of change to impervious surface	Not($\geq$ 1)
**C21 WaterBodyMari**	P1 MCD12Q1.006 MODIS(IGBP)	17. Water Bodies: at least 60% of area is covered by permanent water bodies.
	P2 MCD12Q1.006 MODIS(UMD)	0. Water Bodies: at least 60% of area is covered by permanent water bodies.
	P3 MCD12Q1.006 MODIS(LAI)	0. Water Bodies: at least 60% of area is covered by permanent water bodies.
	P4 MCD12Q1.006 MODIS(BGC))	0. Water Bodies: at least 60% of area is covered by permanent water bodies.
	P5 MCD12Q1.006 MODIS(PFT))	0. Water Bodies: at least 60% of area is covered by permanent water bodies
	P6 CGLS-LC100 collection 3	200. Oceans, seas. Can be either fresh or salt-water bodies.
	P7 Global Forest Cover Change (GFCC)	NA
	P8 GlobCover	210. Water bodies
	P9 GFSAD1000	NA
	P10 Global PALSAR-2/PALSAR(fnf)	3. Water
	P11 Hansen Global Forest Change	NA
	P12 Global Forest Canopy Height	NA
	P13 JRC Yearly Water Classification History	3. Permanent water
	P14 JRC Global Surface Water Mapping Layers	1. max_extent
	P15 Tsinghua FROM-GLC year of change to impervious surface	Not($\geq$ 1)

**C22 WaterBodyCont**	P1 MCD12Q1.006 MODIS(IGBP)	17. Water Bodies: at least 60% of area is covered by permanent water bodies.
	P2 MCD12Q1.006 MODIS(UMD)	0. Water Bodies: at least 60% of area is covered by permanent water bodies.
	P3 MCD12Q1.006 MODIS(LAI)	0. Water Bodies: at least 60% of area is covered by permanent water bodies.
	P4 MCD12Q1.006 MODIS(BGC))	0. Water Bodies: at least 60% of area is covered by permanent water bodies.
	P5 MCD12Q1.006 MODIS(PFT))	0. Water Bodies: at least 60% of area is covered by permanent water bodies
	P6 CGLS-LC100 collection 3	80. Permanent water bodies. Lakes, reservoirs, and rivers. Can be either fresh or salt-water bodies.
	P7 Global Forest Cover Change (GFCC)	NA
	P8 GlobCover	210. Water bodies
	P9 GFSAD1000	NA
	P10 Global PALSAR-2/PALSAR(fnf)	3. Water
	P11 Hansen Global Forest Change	NA
	P12 Global Forest Canopy Height	NA
	P13 JRC Yearly Water Classification History	3. Permanent water
	P14 JRC Global Surface Water Mapping Layers	1. max_extent
	P15 Tsinghua FROM-GLC year of change to impervious surface	Not(≥ 1)
**C23 PermanentSnow**	P1 MCD12Q1.006 MODIS(IGBP)	15.Permanent Snow and Ice: at least 60% of area is covered by snow and ice for at least 10 months of the year.
	P2 MCD12Q1.006 MODIS(UMD)	NA
	P3 MCD12Q1.006 MODIS(LAI)	NA
	P4 MCD12Q1.006 MODIS(BGC))	NA
	P5 MCD12Q1.006 MODIS(PFT))	10. Permanent Snow and Ice: at least 60% of area is covered by snow and ice for at least 10 months of the year.
	P6 CGLS-LC100 collection 3	70. Snow and ice. Lands under snow or ice cover throughout the year.
	P7 Global Forest Cover Change (GFCC)	NA
	P8 GlobCover	220. Permanent snow and ice
	P9 GFSAD1000	NA
	P10 Global PALSAR-2/PALSAR(fnf)	NA
	P11 Hansen Global Forest Change	NA
	P12 Global Forest Canopy Height	NA
	P13 JRC Yearly Water Classification History	(1. Not water) OR (0. No data)
	P14 JRC Global Surface Water Mapping Layers	0. max_extent
	P15 Tsinghua FROM-GLC year of change to impervious surface	Not(≥ 1)
**C24 CropSeasWater**	P1 MCD12Q1.006 MODIS(IGBP)	12. Croplands: at least 60% of area is cultivated cropland.
	P2 MCD12Q1.006 MODIS(UMD)	12. Croplands: at least 60% of area is cultivated cropland.
	P3 MCD12Q1.006 MODIS(LAI)	(3. Broadleaf Croplands: dominated by herbaceous annuals (<2m) that are cultivated with broadleaf crops.) OR<br>(1. Grasslands: dominated by herbaceous annuals (<2m) including cereal croplands.)
	P4 MCD12Q1.006 MODIS(BGC))	(5. Annual Broadleaf Vegetation: dominated by herbaceous annuals (<2m). At least 60% cultivated broadleaf crops.) OR<br>(6. Annual Grass Vegetation: dominated by herbaceous annuals (<2m) including cereal croplands.)
	P5 MCD12Q1.006 MODIS(PFT))	(7. Cereal Croplands: dominated by herbaceous annuals (<2m). At least 60% cultivated cereal crops.) OR<br>(8. Broadleaf Croplands: dominated by herbaceous annuals (<2m). At least 60% cultivated broadleaf crops.)
	P6 CGLS-LC100 collection 3	40. Cultivated and managed vegetation / agriculture. Lands covered with temporary crops followed by harvest<br>and a bare soil period (e.g., single and multiple cropping systems). Note that perennial woody crops will be classified<br>as the appropriate forest or shrub land cover type.
	P7 Global Forest Cover Change (GFCC)	NA
	P8 GlobCover	(11. Post-flooding or irrigated croplands) OR (14. Rainfed croplands)
	P9 GFSAD1000	(1. Croplands: irrigation major) OR (2. Croplands: irrigation minor) OR (3. Croplands: rainfed) OR<br>(4. Croplands: rainfed, minor fragments) OR (5. Croplands: rainfed, very minor fragments)
	P10 Global PALSAR-2/PALSAR(fnf)	NA
	P11 Hansen Global Forest Change	NA
	P12 Global Forest Canopy Height	NA
	P13 JRC Yearly Water Classification History	(2. Seasonal water) OR (3. Permanent water)
	P14 JRC Global Surface Water Mapping Layers	(0. No change) OR (4. Seasonal) OR (8. Permanent to seasonal) OR (10. Ephemeral seasonal)
	P15 Tsinghua FROM-GLC year of change to impervious surface	Not(≥ 1)
**C25 CropCereaIrri**	P1 MCD12Q1.006 MODIS(IGBP)	12. Croplands: at least 60% of area is cultivated cropland.
	P2 MCD12Q1.006 MODIS(UMD)	12. Croplands: at least 60% of area is cultivated cropland.
	P3 MCD12Q1.006 MODIS(LAI)	1. Grasslands: dominated by herbaceous annuals (<2m) including cereal croplands.
	P4 MCD12Q1.006 MODIS(BGC))	6. Annual Grass Vegetation: dominated by herbaceous annuals (<2m) including cereal croplands.
	P5 MCD12Q1.006 MODIS(PFT))	7. Cereal Croplands: dominated by herbaceous annuals (<2m). At least 60% cultivated cereal crops.
	P6 CGLS-LC100 collection 3	40. Cultivated and managed vegetation / agriculture. Lands covered with temporary crops followed by harvest<br>and a bare soil period (e.g., single and multiple cropping systems). Note that perennial woody crops will be classified<br>as the appropriate forest or shrub land cover type.
	P7 Global Forest Cover Change (GFCC)	NA
	P8 GlobCover	11. Post-flooding or irrigated croplands
	P9 GFSAD1000	(1. Croplands: irrigation major) OR (2. Croplands: irrigation minor)
	P10 Global PALSAR-2/PALSAR(fnf)	NA
	P11 Hansen Global Forest Change	NA
	P12 Global Forest Canopy Height	NA
	P13 JRC Yearly Water Classification History	(1. Not water) OR (0. No data)
	P14 JRC Global Surface Water Mapping Layers	0. max_extent
	P15 Tsinghua FROM-GLC year of change to impervious surface	Not(≥ 1)
**C26 CropCereaRain**	P1 MCD12Q1.006 MODIS(IGBP)	12. Croplands: at least 60% of area is cultivated cropland.
	P2 MCD12Q1.006 MODIS(UMD)	12. Croplands: at least 60% of area is cultivated cropland.
	P3 MCD12Q1.006 MODIS(LAI)	1. Grasslands: dominated by herbaceous annuals (<2m) including cereal croplands.
	P4 MCD12Q1.006 MODIS(BGC))	6. Annual Grass Vegetation: dominated by herbaceous annuals (<2m) including cereal croplands.
	P5 MCD12Q1.006 MODIS(PFT))	7. Cereal Croplands: dominated by herbaceous annuals (<2m). At least 60% cultivated cereal crops.
	P6 CGLS-LC100 collection 3	40. Cultivated and managed vegetation / agriculture. Lands covered with temporary crops followed by harvest<br>and a bare soil period (e.g., single and multiple cropping systems). Note that perennial woody crops will be classified<br>as the appropriate forest or shrub land cover type.
	P7 Global Forest Cover Change (GFCC)	NA
	P8 GlobCover	14. Rainfed croplands
	P9 GFSAD1000	(3. Croplands: rainfed) OR (4. Croplands: rainfed, minor fragments) OR (5. Croplands: rainfed, very minor fragments)
	P10 Global PALSAR-2/PALSAR(fnf)	NA
	P11 Hansen Global Forest Change	NA
	P12 Global Forest Canopy Height	NA
	P13 JRC Yearly Water Classification History	(1. Not water) OR (0. No data)
	P14 JRC Global Surface Water Mapping Layers	0. max_extent
	P15 Tsinghua FROM-GLC year of change to impervious surface	Not(≥ 1)
**C27 CropBroadIrri**	P1 MCD12Q1.006 MODIS(IGBP)	12. Croplands: at least 60% of area is cultivated cropland.
	P2 MCD12Q1.006 MODIS(UMD)	12. Croplands: at least 60% of area is cultivated cropland.
	P3 MCD12Q1.006 MODIS(LAI)	3. Broadleaf Croplands: dominated by herbaceous annuals (<2m) that are cultivated with broadleaf crops.
	P4 MCD12Q1.006 MODIS(BGC))	5. Annual Broadleaf Vegetation: dominated by herbaceous annuals (<2m). At least 60% cultivated broadleaf crops.
	P5 MCD12Q1.006 MODIS(PFT))	8. Broadleaf Croplands: dominated by herbaceous annuals (<2m). At least 60% cultivated broadleaf crops.
	P6 CGLS-LC100 collection 3	40. Cultivated and managed vegetation / agriculture. Lands covered with temporary crops followed by harvest<br>and a bare soil period (e.g., single and multiple cropping systems). Note that perennial woody crops will be classified<br>as the appropriate forest or shrub land cover type.
	P7 Global Forest Cover Change (GFCC)	NA
	P8 GlobCover	11. Post-flooding or irrigated croplands
	P9 GFSAD1000	(1. Croplands: irrigation major) OR (2. Croplands: irrigation minor)
	P10 Global PALSAR-2/PALSAR(fnf)	NA
	P11 Hansen Global Forest Change	NA
	P12 Global Forest Canopy Height	NA
	P13 JRC Yearly Water Classification History	(1. Not water) OR (0. No data)
	P14 JRC Global Surface Water Mapping Layers	0. max_extent
	P15 Tsinghua FROM-GLC year of change to impervious surface	Not(≥ 1)

**C28 CropBroadRain**	P1 MCD12Q1.006 MODIS(IGBP)	12. Croplands: at least 60% of area is cultivated cropland.
	P2 MCD12Q1.006 MODIS(UMD)	12. Croplands: at least 60% of area is cultivated cropland.
	P3 MCD12Q1.006 MODIS(LAI)	3. Broadleaf Croplands: dominated by herbaceous annuals (<2m) that are cultivated with broadleaf crops.
	P4 MCD12Q1.006 MODIS(BGC))	5. Annual Broadleaf Vegetation: dominated by herbaceous annuals (<2m). At least 60% cultivated broadleaf crops.
	P5 MCD12Q1.006 MODIS(PFT))	8. Broadleaf Croplands: dominated by herbaceous annuals (<2m). At least 60% cultivated broadleaf crops.
	P6 CGLS-LC100 collection 3	40. Cultivated and managed vegetation / agriculture. Lands covered with temporary crops followed by harvest and a bare soil period (e.g., single and multiple cropping systems). Note that perennial woody crops will be classified as the appropriate forest or shrub land cover type.
	P7 Global Forest Cover Change (GFCC)	NA
	P8 GlobCover	14. Rainfed croplands
	P9 GFSAD1000	(3. Croplands: rainfed) OR (4. Croplands: rainfed, minor fragments) OR (5. Croplands: rainfed, very minor fragments)
	P10 Global PALSAR-2/PALSAR(fnf)	NA
	P11 Hansen Global Forest Change	NA
	P12 Global Forest Canopy Height	NA
	P13 JRC Yearly Water Classification History	(1. Not water) OR (0. No data)
	P14 JRC Global Surface Water Mapping Layers	0. max_extent
	P15 Tsinghua FROM-GLC year of change to impervious surface	Not($\geq$ 1)
**C29 UrbanBlUpArea**	P1 MCD12Q1.006 MODIS(IGBP)	13. Urban and Built-up Lands: at least 30% impervious surface area including building materials, asphalt and vehicles.
	P2 MCD12Q1.006 MODIS(UMD)	13. Urban and Built-up Lands: at least 30% impervious surface area including building materials, asphalt and vehicles.
	P3 MCD12Q1.006 MODIS(LAI)	10. Urban and Built-up Lands: at least 30% impervious surface area including building materials, asphalt and vehicles.
	P4 MCD12Q1.006 MODIS(BGC))	8. Urban and Built-up Lands: at least 30% impervious surface area including building materials, asphalt, and vehicles.
	P5 MCD12Q1.006 MODIS(PFT))	9. Urban and Built-up Lands: at least 30% impervious surface area including building materials, asphalt, and vehicles.
	P6 CGLS-LC100 collection 3	50. Urban / built up. Land covered by buildings and other man-made structures.
	P7 Global Forest Cover Change (GFCC)	NA
	P8 GlobCover	190. Artificial surfaces and associated areas (urban areas >50%)
	P9 GFSAD1000	NA
	P10 Global PALSAR-2/PALSAR(fnf)	NA
	P11 Hansen Global Forest Change	NA
	P12 Global Forest Canopy Height	NA
	P13 JRC Yearly Water Classification History	(1. Not water) OR (0. No data)
	P14 JRC Global Surface Water Mapping Layers	0. max_extent
	P15 Tsinghua FROM-GLC year of change to impervious surface	NU
