# Supplementary material for: Sentinel2GlobalLULC: A Sentinel-2 RGB image tile dataset for global land use/cover mapping with deep learning
Source: Sci Data. 2022 Nov 9;9:681. doi: 10.1038/s41597-022-01775-8 (PMC9646844; doi:10.1038/s41597-022-01775-8)
Supplement: Supplementary file 3 [file 41597_2022_1775_MOESM3_ESM.pdf]

Supplementary File 3

Algorithm 1 Selection of evenly distributed pixels for one LULC class

```
Load the metadata of one LULC class
Put the coordinates of the LULC class in Data_Points
Choose an initial random point from Data_Points and add it to Selected_Points
while |Selected_Points| < 14000 do
    for Point in Data_Points do
        Compute the distance between Point and Selected_Points
        Get the minimum distance and add it to Distance_To_Closest_Points
    end for
    Get the index of the maximum distance:
            argmax(Distance_To_Closest_Points)
    Add the corresponding Point to Selected_Points
end while
Save Selected_Points to download their corresponding RGB images in GEE
```
