# Supplementary material for: Sentinel2GlobalLULC: A Sentinel-2 RGB image tile dataset for global land use/cover mapping with deep learning
Source: Sci Data. 2022 Nov 9;9:681. doi: 10.1038/s41597-022-01775-8 (PMC9646844; doi:10.1038/s41597-022-01775-8)
Supplement: Supplementary file 4 [file 41597_2022_1775_MOESM4_ESM.pdf]

Supplementary File 4

In this Supplementary File, we present 29 figures that highlight the number of aggregated Sentinel2 images to build images in each LULC class (from C1 to C29).

List of Figures

1 The number of Sentinel-2 images used to build each image in C1 Barren. This number is represented under different intervals. . . . 3
2 The number of Sentinel-2 images used to build each image in C2 Moss and Lichen. This number is represented under different intervals. . . . 4
3 The number of Sentinel-2 images used to build each image in C3 Grassland. This number is represented under different intervals. . . . 5
4 The number of Sentinel-2 images used to build each image in C4 Open Shrublands. This number is represented under different intervals. . . . 6
5 The number of Sentinel-2 images used to build each image in C5 Close Shrublands. This number is represented under different intervals. . . . 7
6 The number of Sentinel-2 images used to build each image in C6 Forest ODB. This number is represented under different intervals. . . . 8
7 The number of Sentinel-2 images used to build each image in C7 Forest CDB. This number is represented under different intervals. . . . 9
8 The number of Sentinel-2 images used to build each image in C8 Forest DDB. This number is represented under different intervals. . . . 10
9 The number of Sentinel-2 images used to build each image in C9 Forest ODN. This number is represented under different intervals. . . . 11
10 The number of Sentinel-2 images used to build each image in C10 Forest CDN. This number is represented under different intervals. . . . 12
11 The number of Sentinel-2 images used to build each image in C11 Forest DDN. This number is represented under different intervals. . . . 13
12 The number of Sentinel-2 images used to build each image in C12 Forest OEB. This number is represented under different intervals. . . . 14
13 The number of Sentinel-2 images used to build each image in C13 Forest CEB. This number is represented under different intervals. . . . 15
14 The number of Sentinel-2 images used to build each image in C14 Forest DEB. This number is represented under different intervals. . . . 16
15 The number of Sentinel-2 images used to build each image in C15 Forest OEN. This number is represented under different intervals. . . . 17
16 The number of Sentinel-2 images used to build each image in C16 Forest CEN. This number is represented under different intervals. . . . 18
17 The number of Sentinel-2 images used to build each image in C17 Forest DEN. This number is represented under different intervals. . . . 19
18 The number of Sentinel-2 images used to build each image in C18 WetlandMangro. This number is represented under different intervals. . . . 20
19 The number of Sentinel-2 images used to build each image in C19 WetlandSwamps. This number is represented under different intervals. . . . 21
20 The number of Sentinel-2 images used to build each image in C20 WetlandMarshl. This number is represented under different intervals. . . . 22
21 The number of Sentinel-2 images used to build each image in C21 WaterBodyMari. This number is represented under different intervals. . . . 23
22 The number of Sentinel-2 images used to build each image in C22 WaterBodyCont. This number is represented under different intervals. . . . 24
23 The number of Sentinel-2 images used to build each image in C23 PermanentSnow. This number is represented under different intervals. . . . 25
24 The number of Sentinel-2 images used to build each image in C24 CropSeasWater. This number is represented under different intervals. . . . 26

25 The number of Sentinel-2 images used to build each image in C25 CropCereaIrri. This number is represented under different intervals. 27
26 The number of Sentinel-2 images used to build each image in C26 CropCereaRain. This number is represented under different intervals. 28
27 The number of Sentinel-2 images used to build each image in C27 CropBroadIrri. This number is represented under different intervals. 29
28 The number of Sentinel-2 images used to build each image in C28 CropBroadRain. This number is represented under different intervals. 30
29 The number of Sentinel-2 images used to build each image in C29 UrbanBlUpArea. This number is represented under different intervals. 31

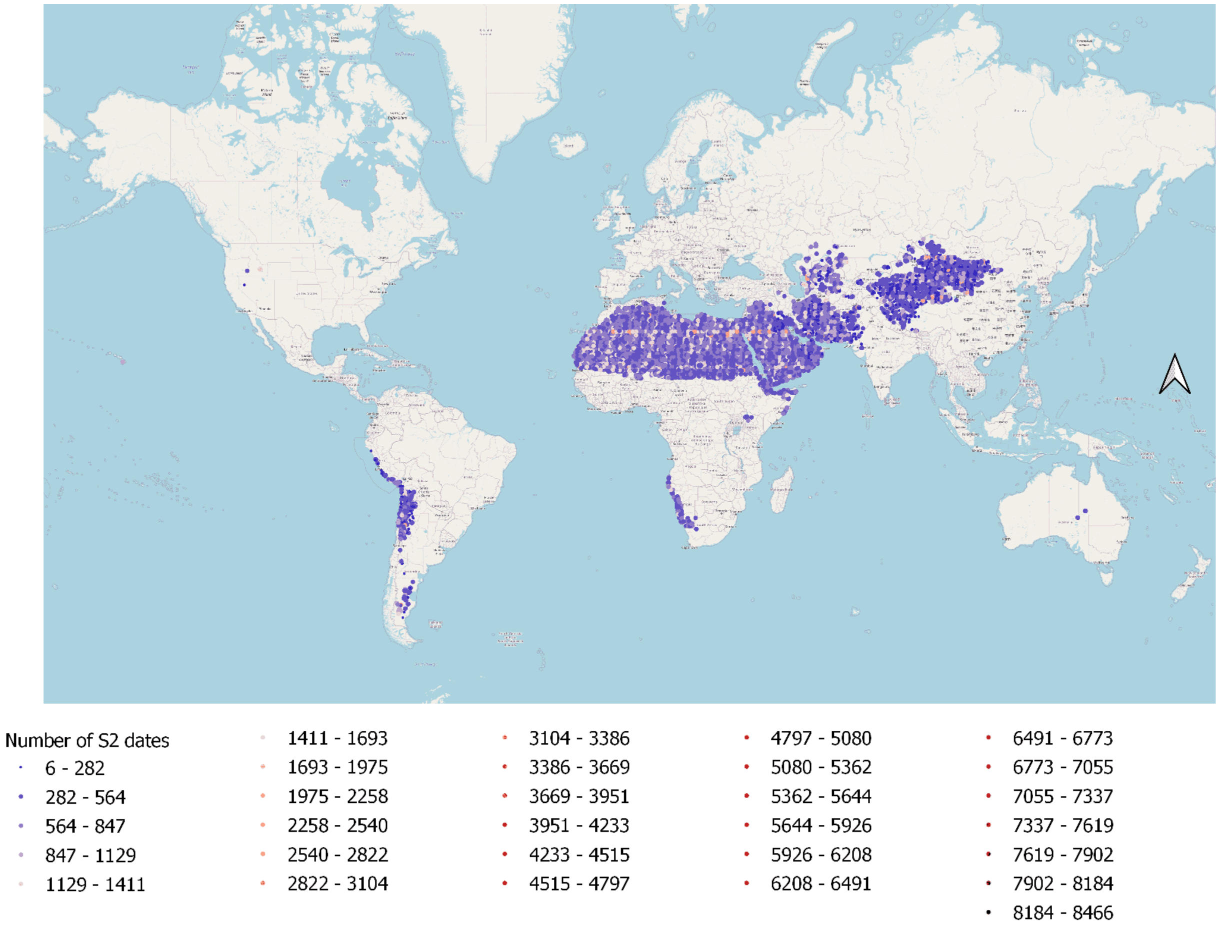

Figure 1. The number of Sentinel-2 images used to build each image in C1 Barren. This number is represented under different intervals.

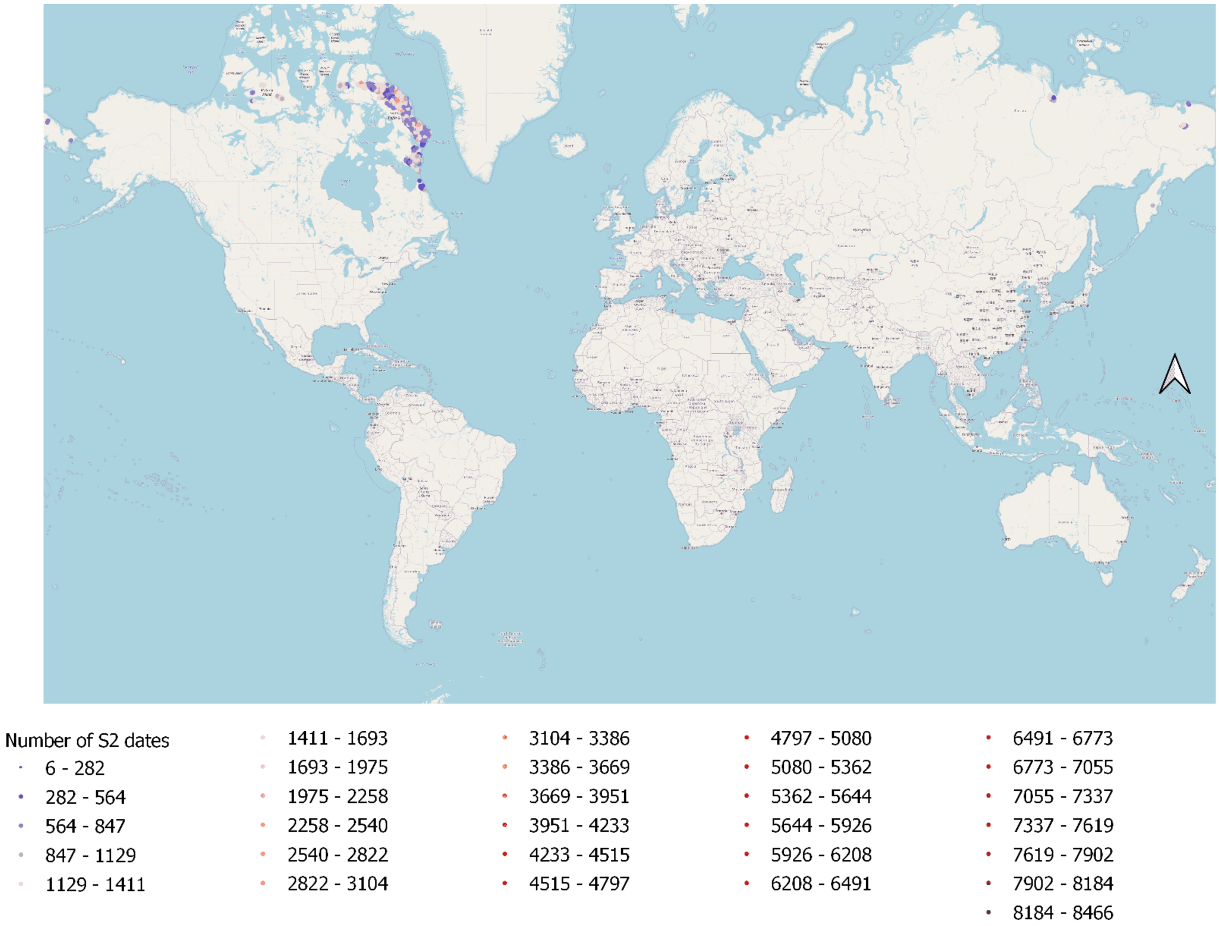

Figure 2. The number of Sentinel-2 images used to build each image in C2 Moss and Lichen. This number is represented under different intervals.

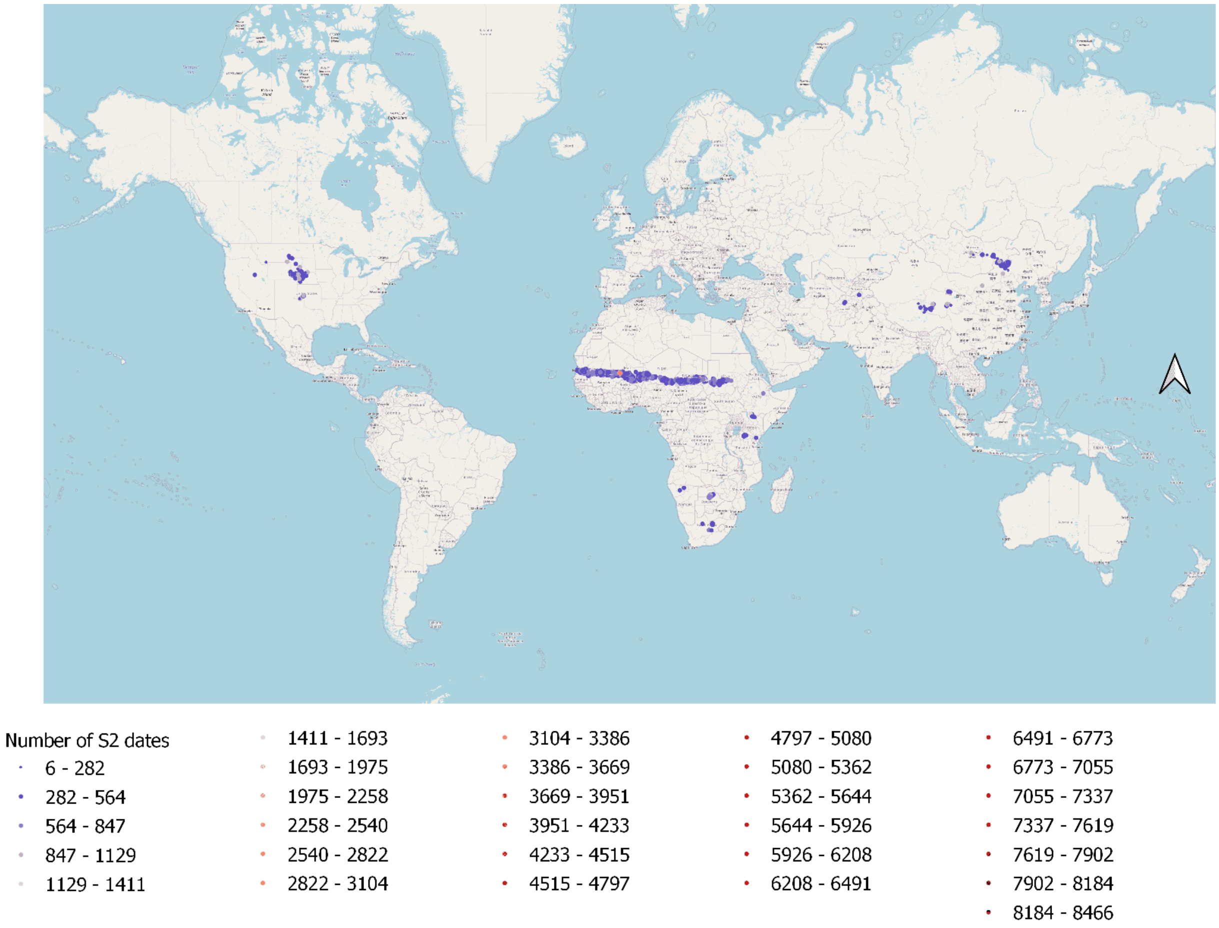

Figure 3. The number of Sentinel-2 images used to build each image in C3 Grassland. This number is represented under different intervals.

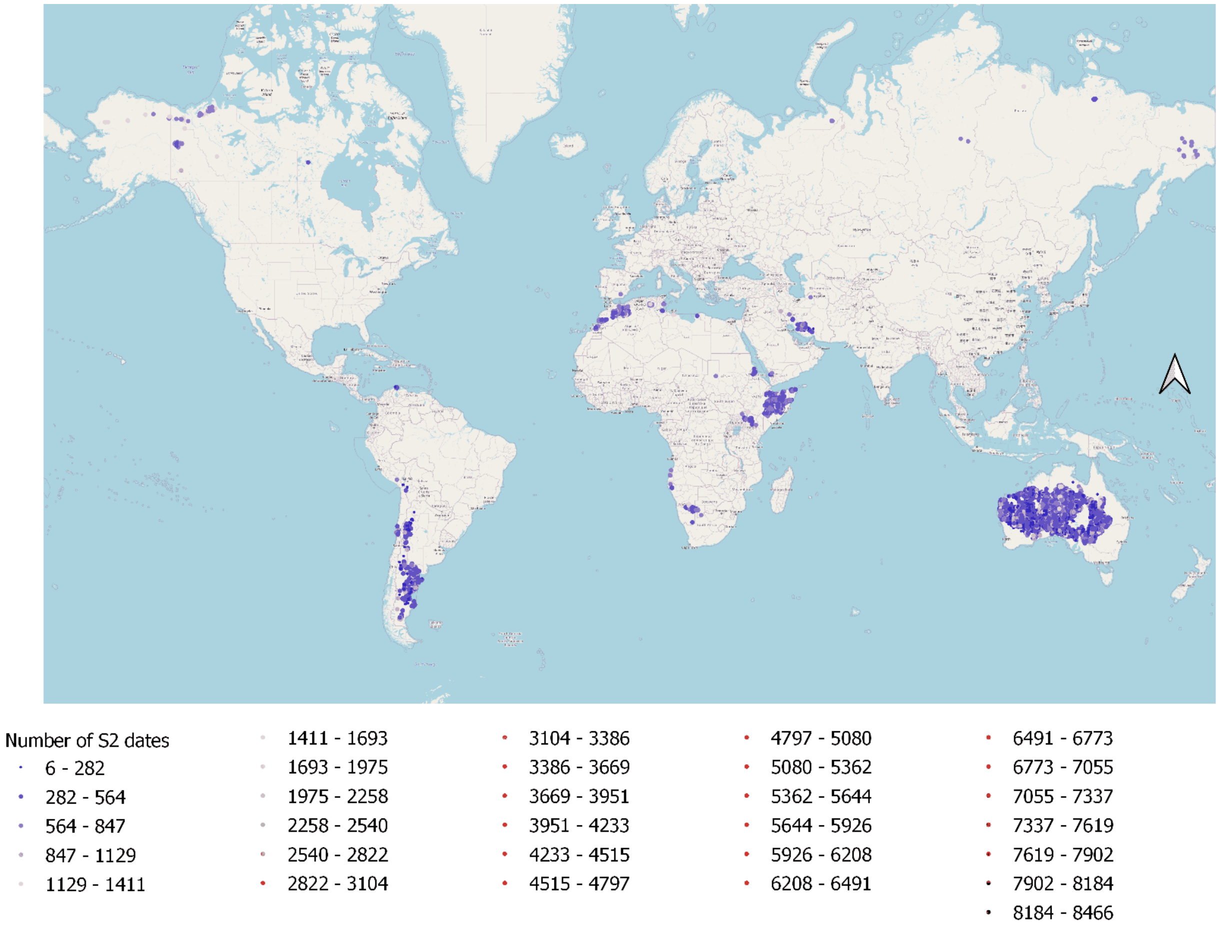

Figure 4. The number of Sentinel-2 images used to build each image in C4 Open Shrublands. This number is represented under different intervals.

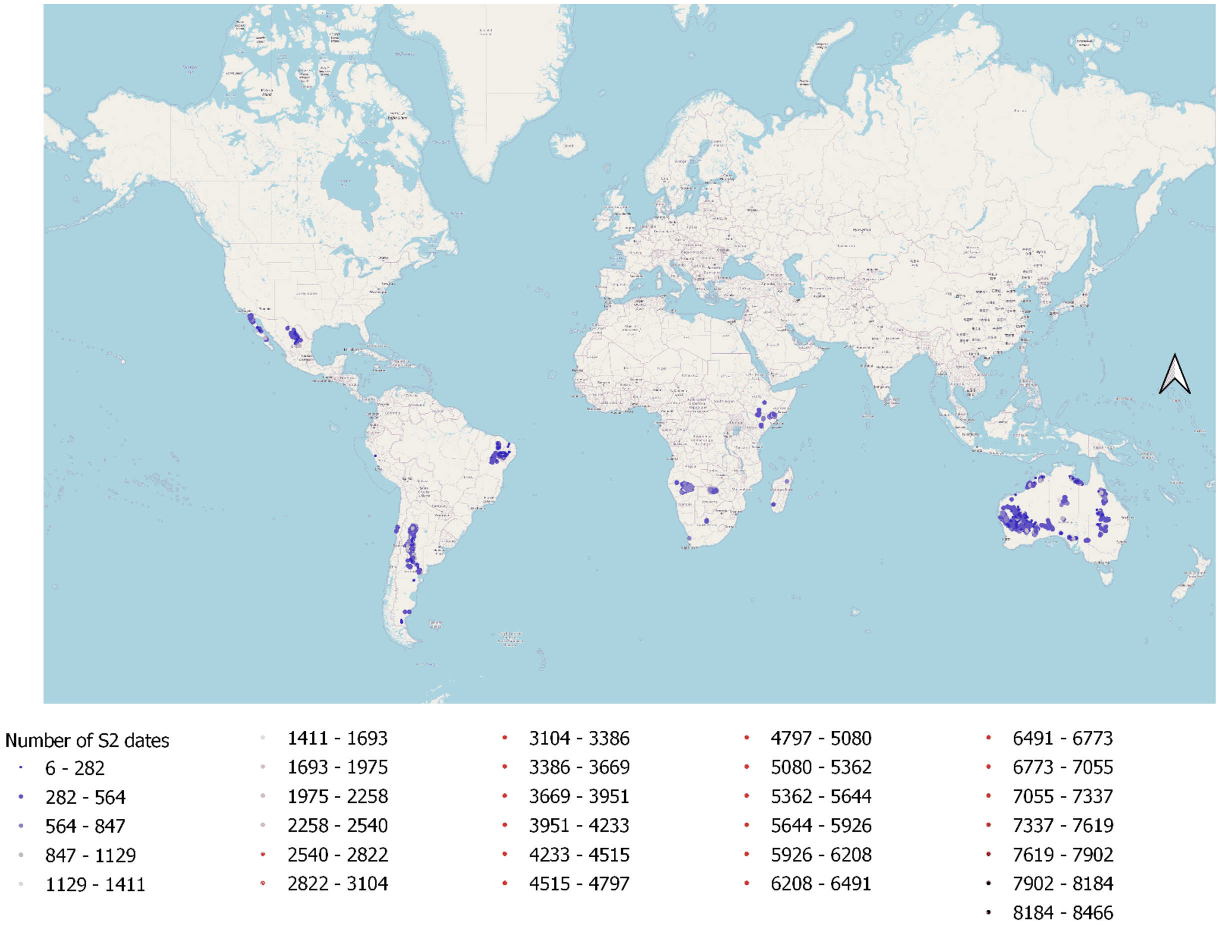

Figure 5. The number of Sentinel-2 images used to build each image in C5 Close Shrublands. This number is represented under different intervals.

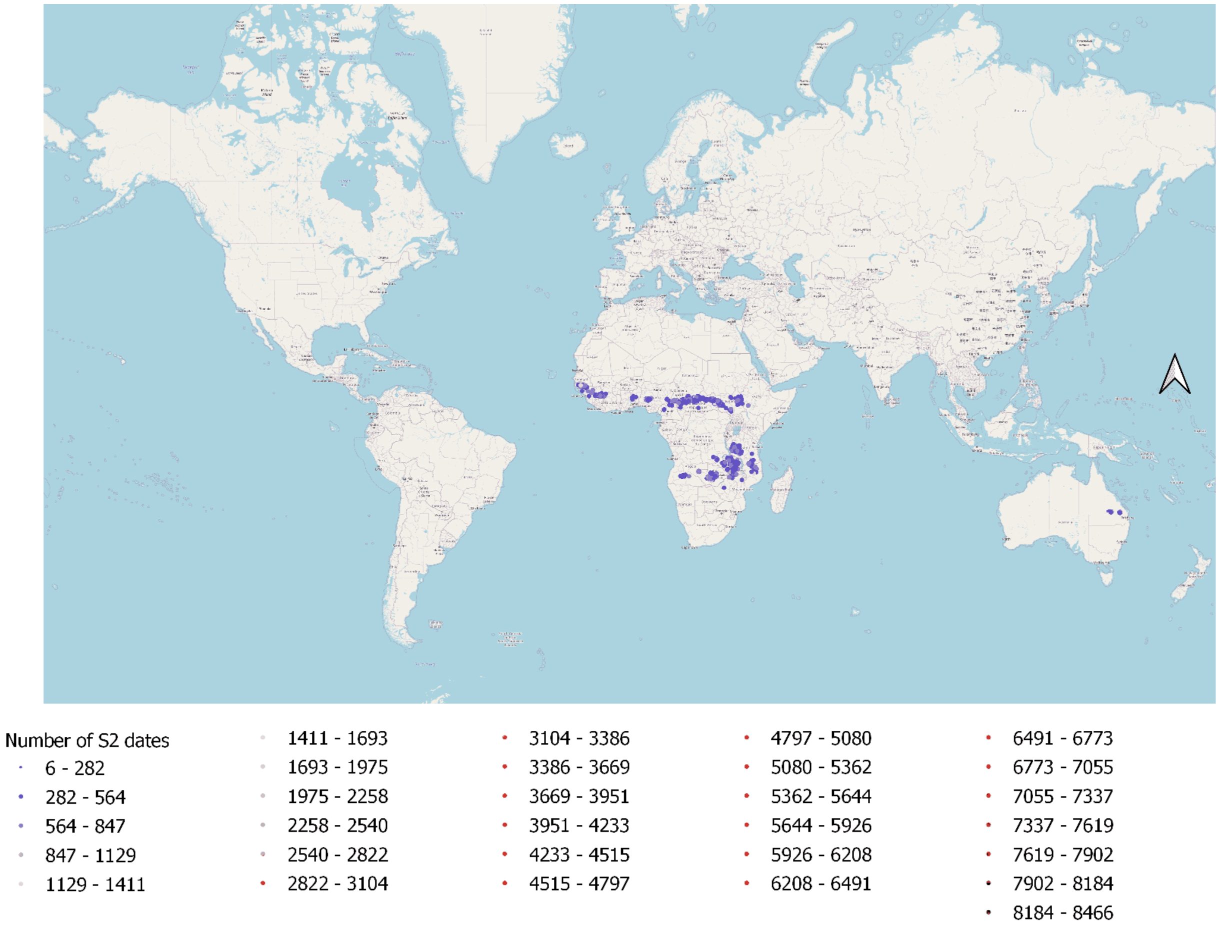

Figure 6. The number of Sentinel-2 images used to build each image in C6 Forest ODB. This number is represented under different intervals.

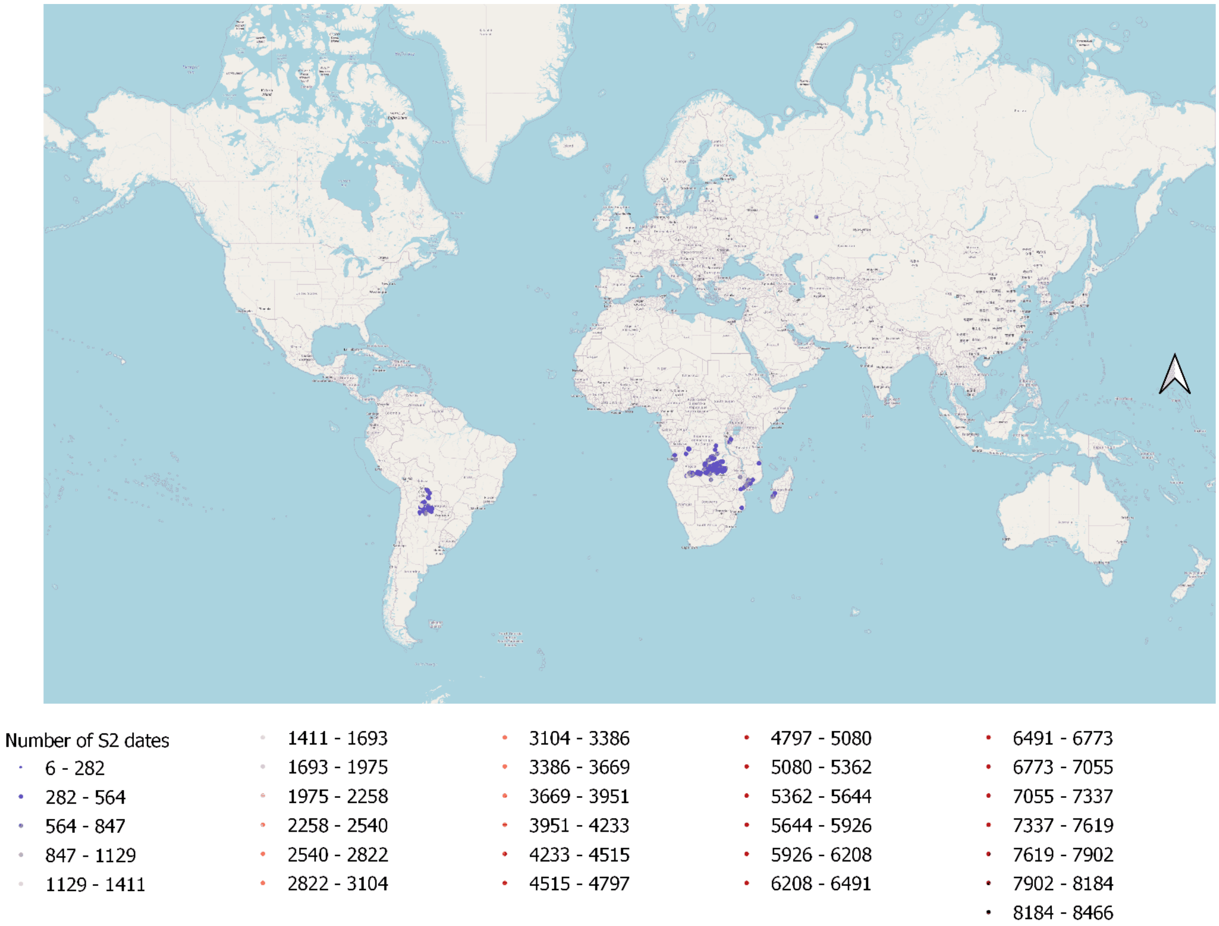

Figure 7. The number of Sentinel-2 images used to build each image in C7 Forest CDB. This number is represented under different intervals.

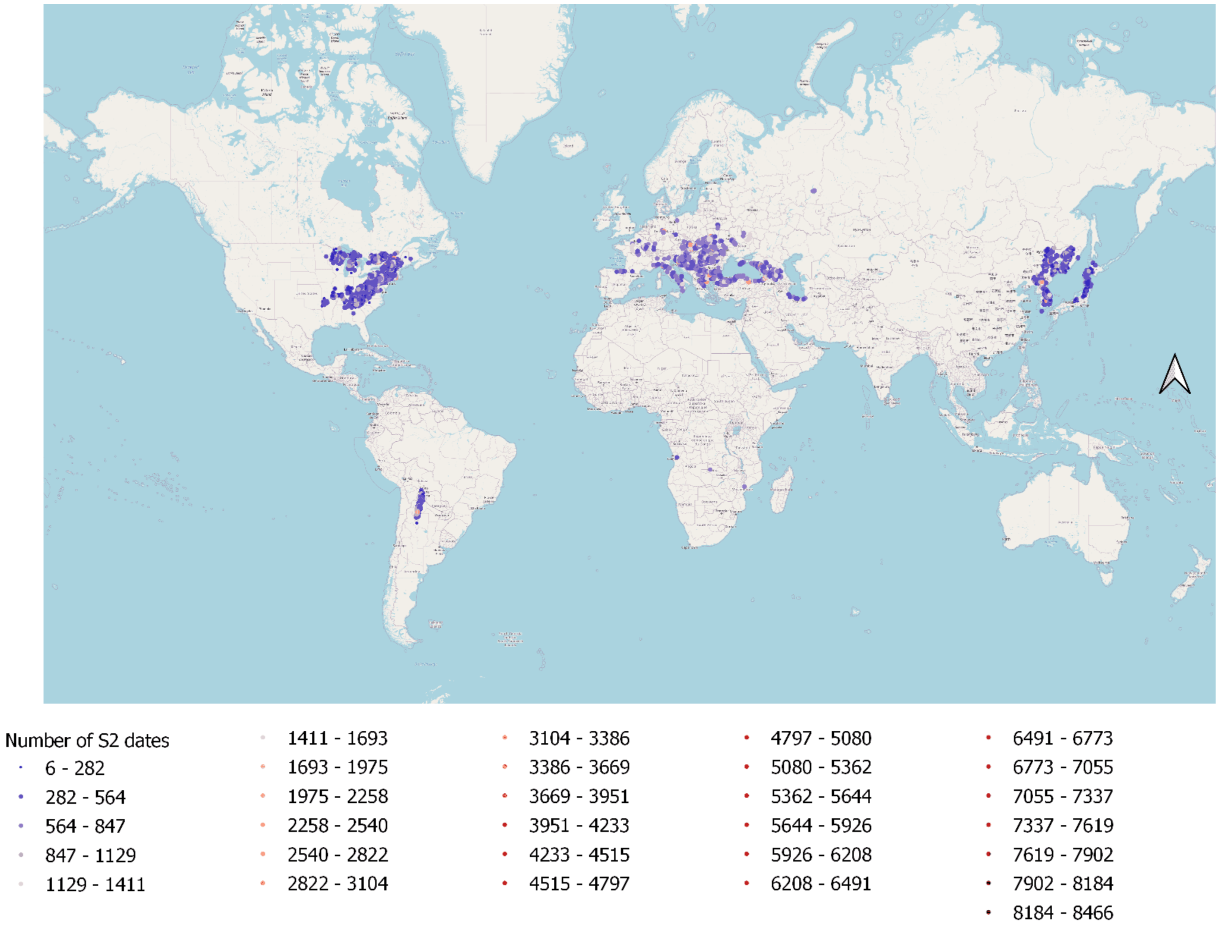

Figure 8. The number of Sentinel-2 images used to build each image in C8 Forest DDB. This number is represented under different intervals.

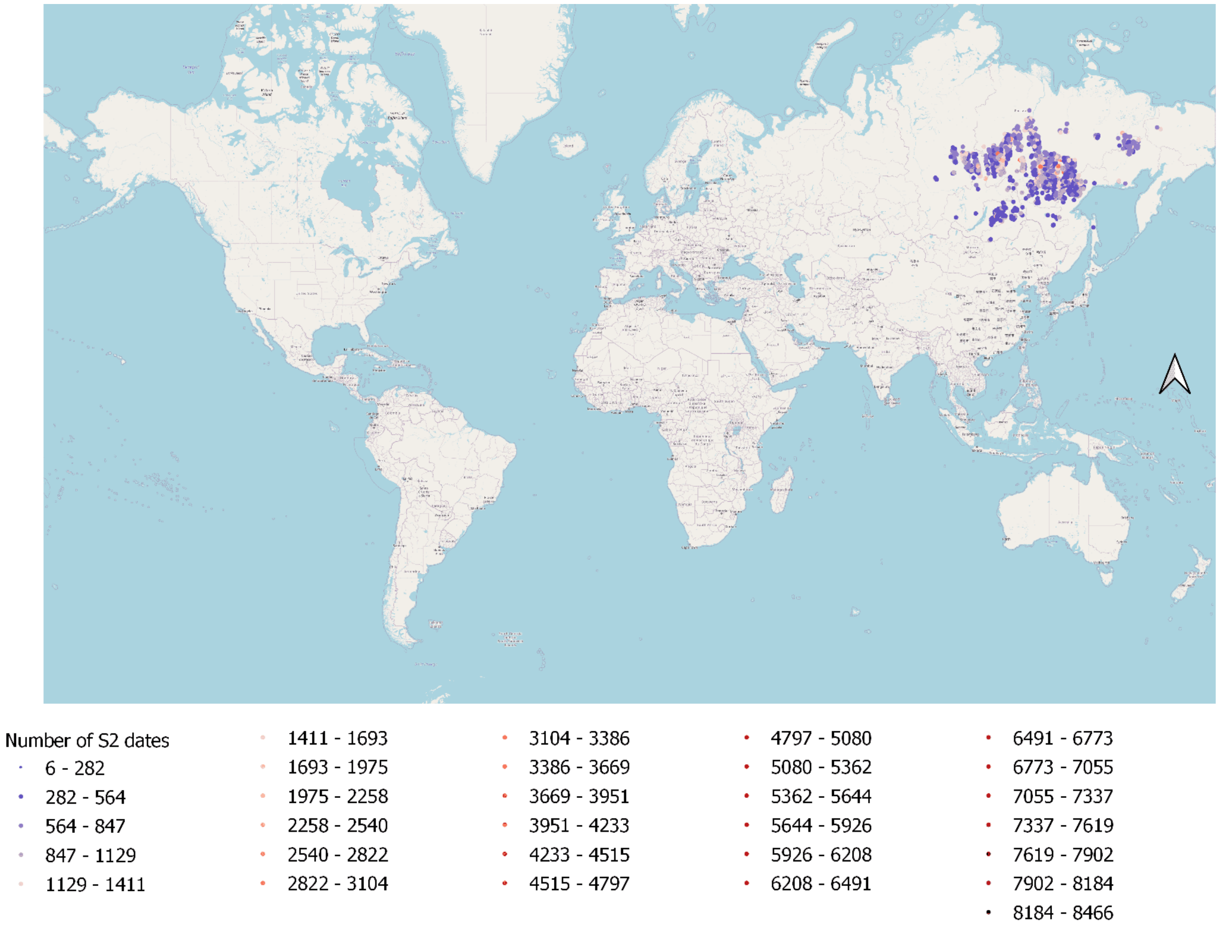

Figure 9. The number of Sentinel-2 images used to build each image in C9 Forest ODN. This number is represented under different intervals.

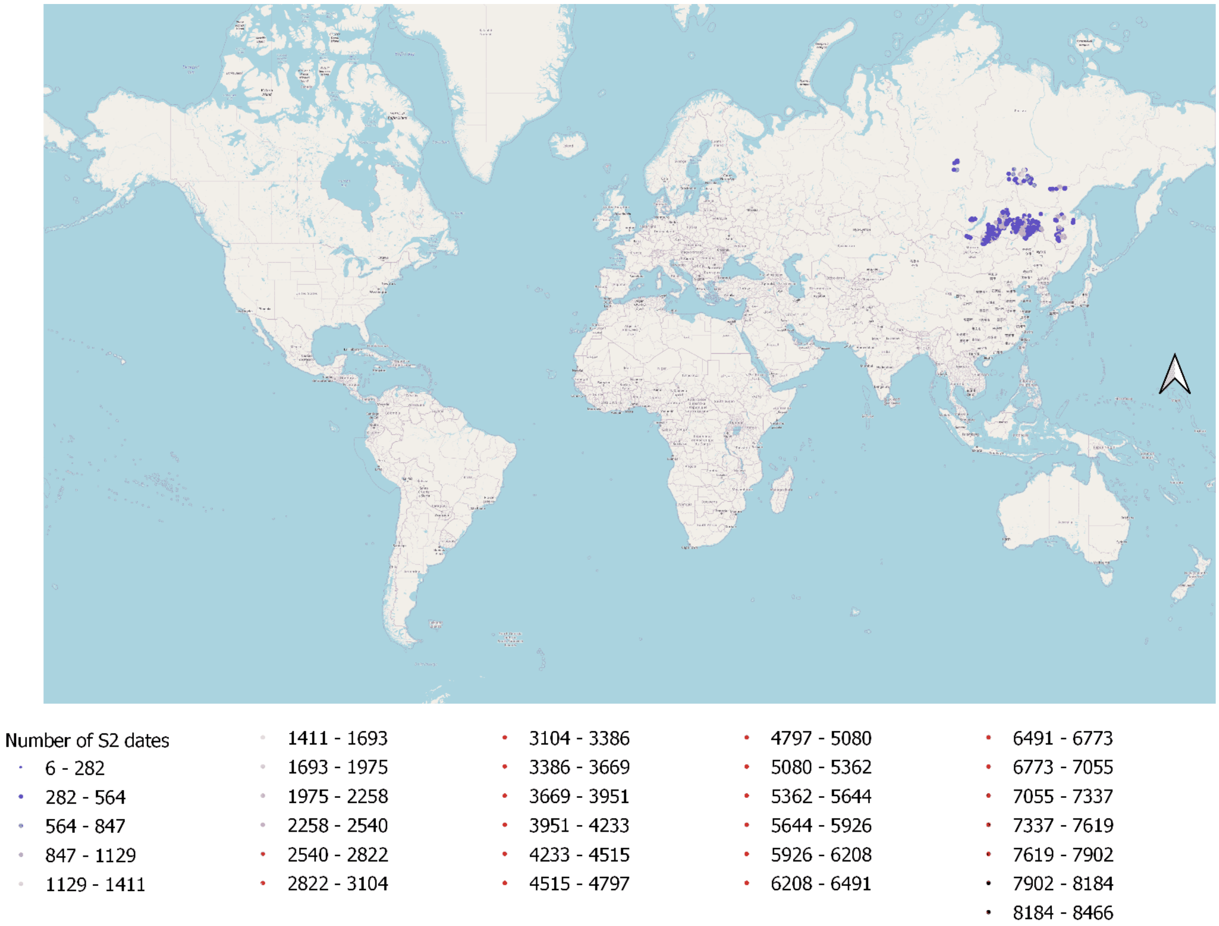

Figure 10. The number of Sentinel-2 images used to build each image in C10 Forest CDN. This number is represented under different intervals.

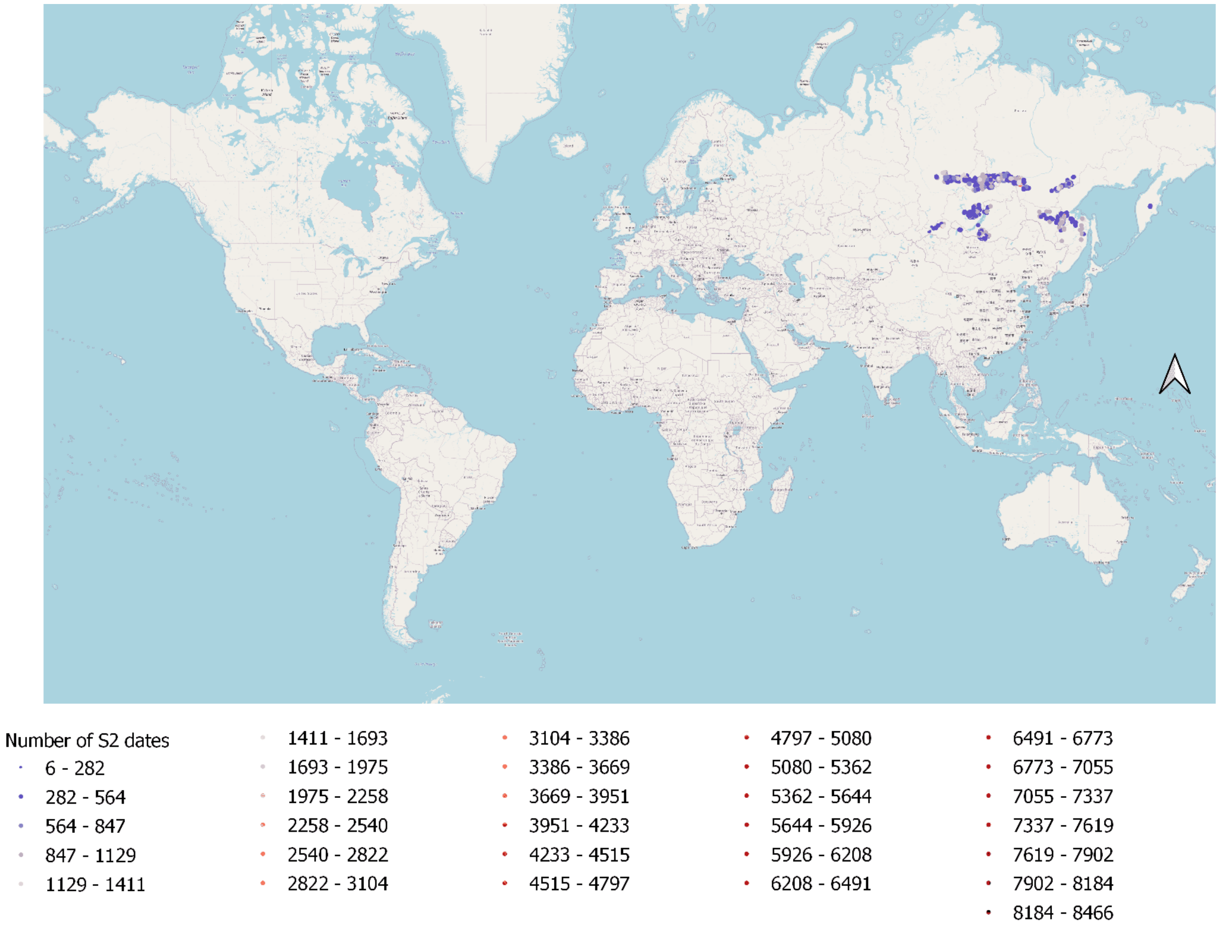

Figure 11. The number of Sentinel-2 images used to build each image in C11 Forest DDN. This number is represented under different intervals.

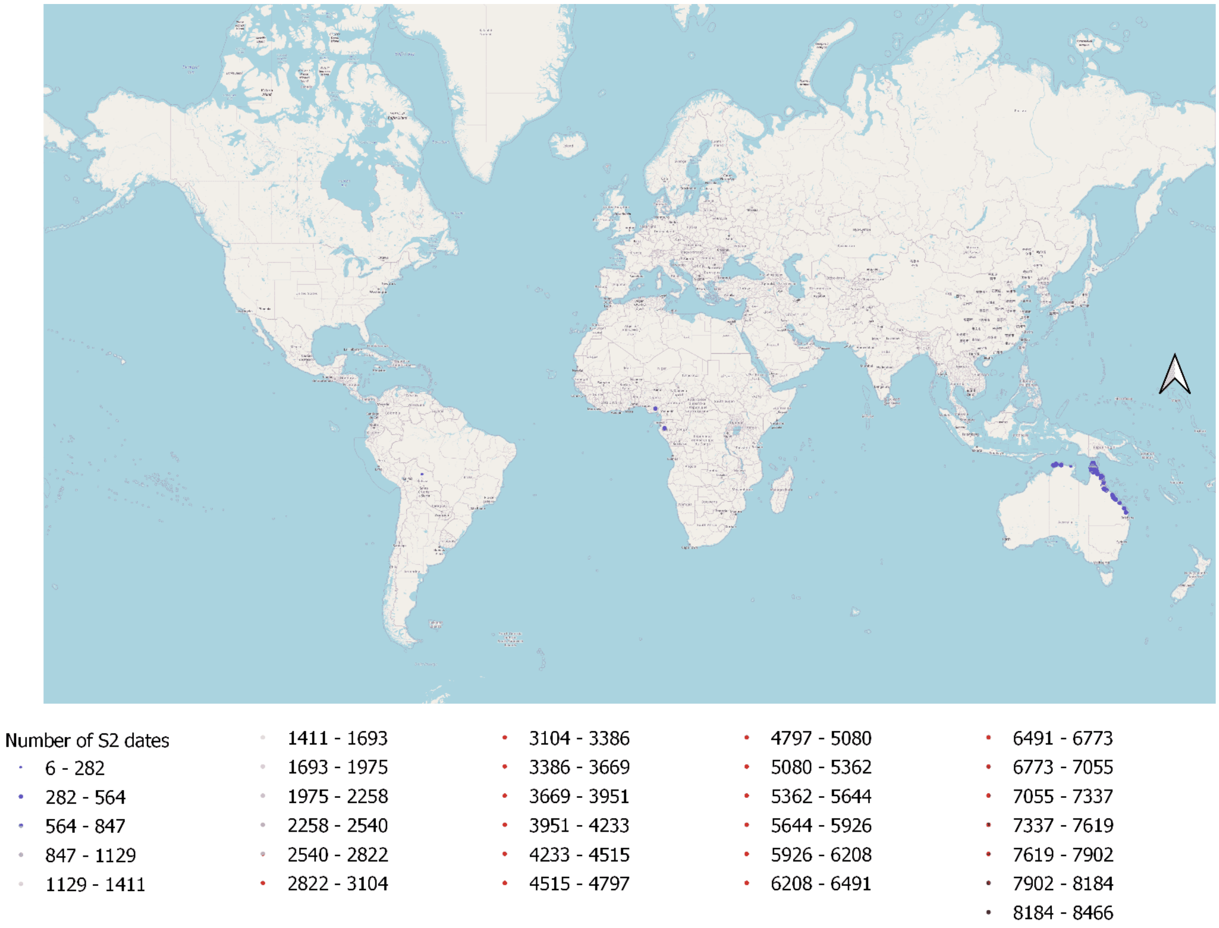

Figure 12. The number of Sentinel-2 images used to build each image in C12 Forest OEB. This number is represented under different intervals.

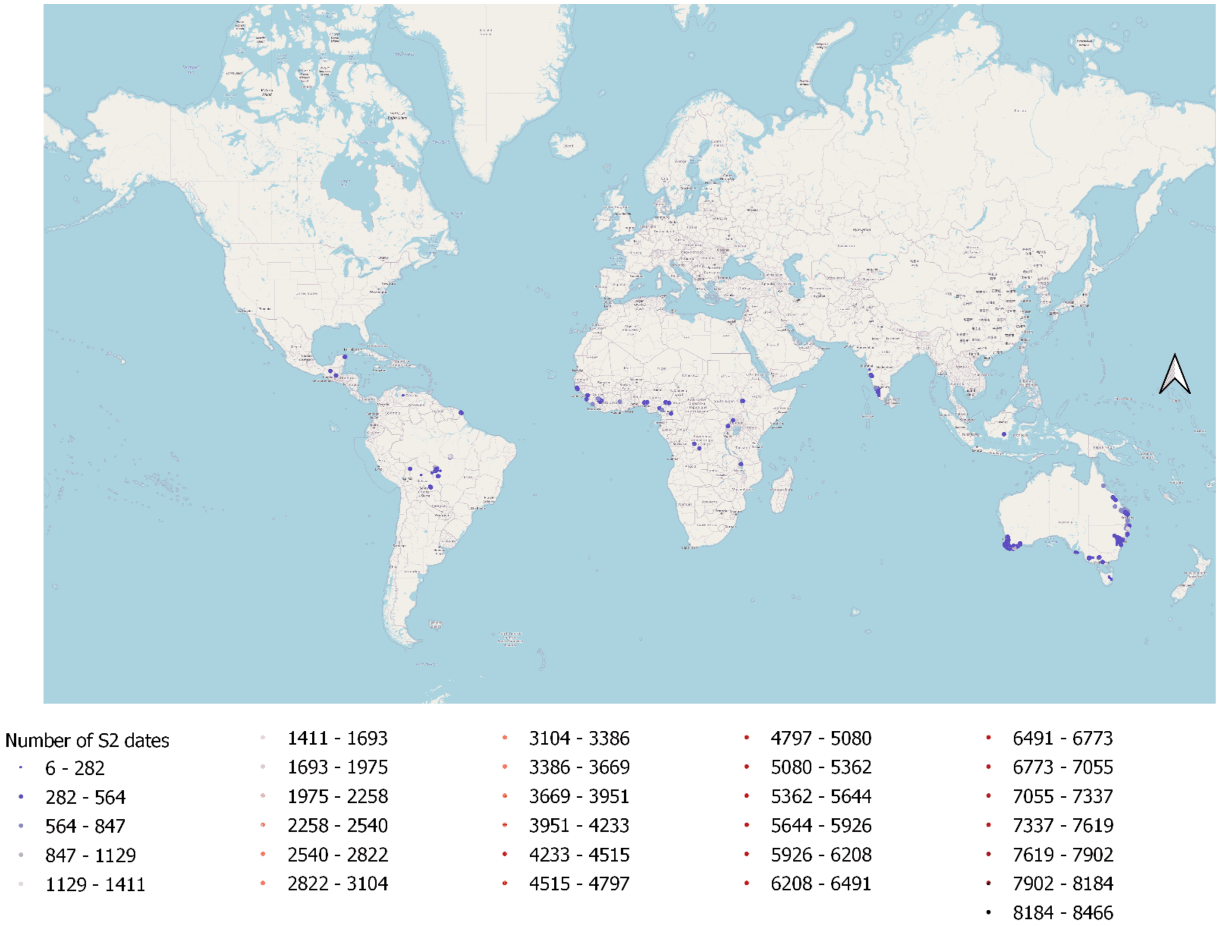

Figure 13. The number of Sentinel-2 images used to build each image in C13 Forest CEB. This number is represented under different intervals.

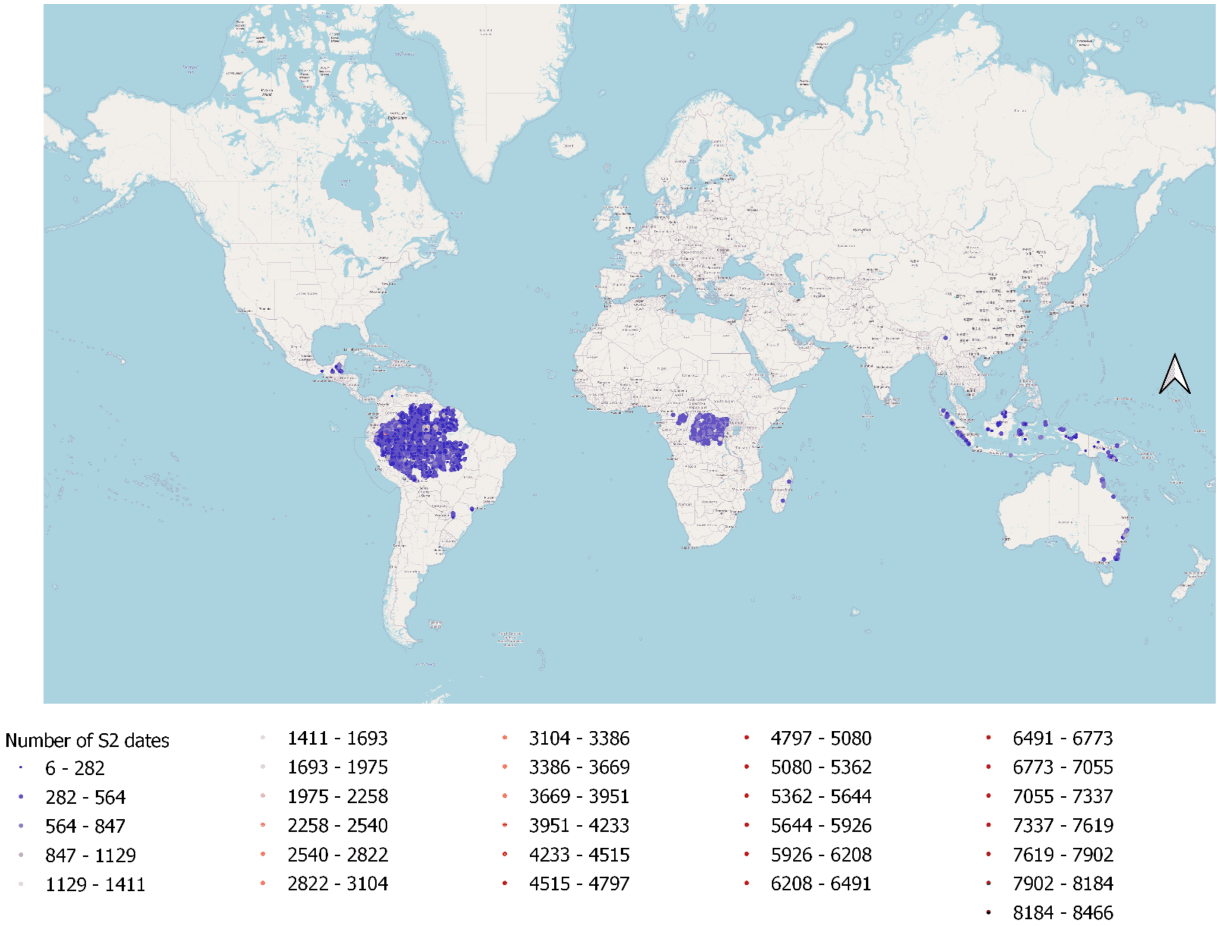

Figure 14. The number of Sentinel-2 images used to build each image in C14 Forest DEB. This number is represented under different intervals.

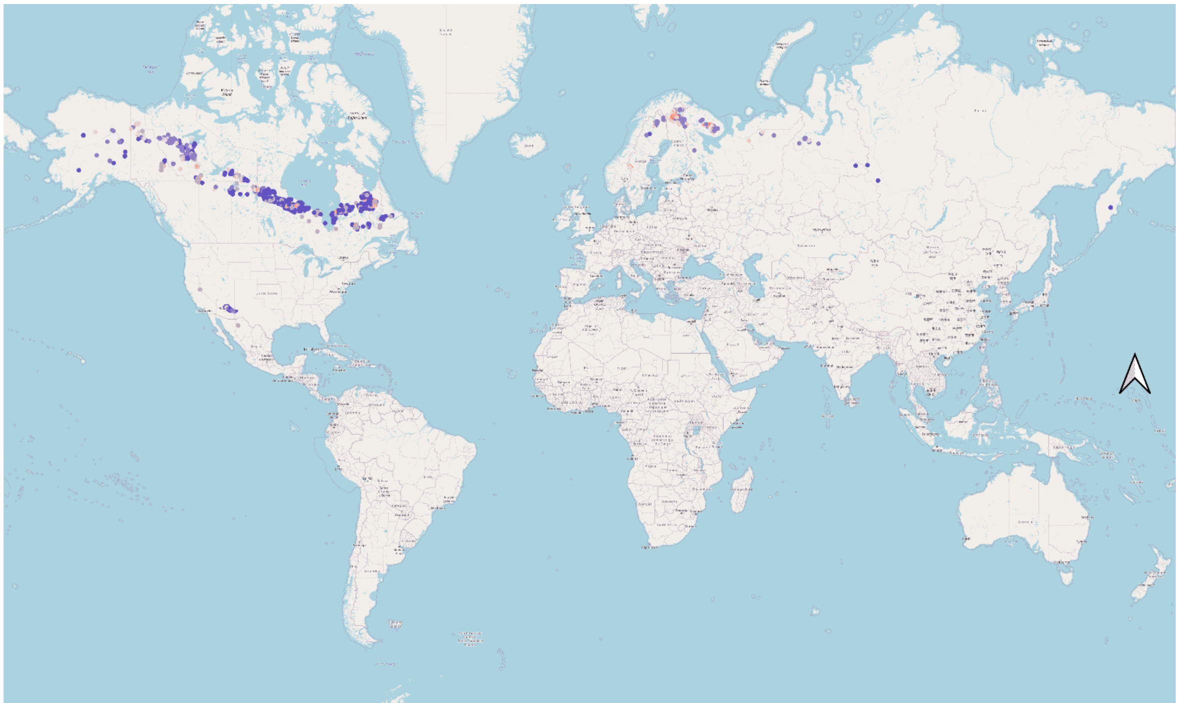

Figure 15. The number of Sentinel-2 images used to build each image in C15 Forest OEN. This number is represented under different intervals.

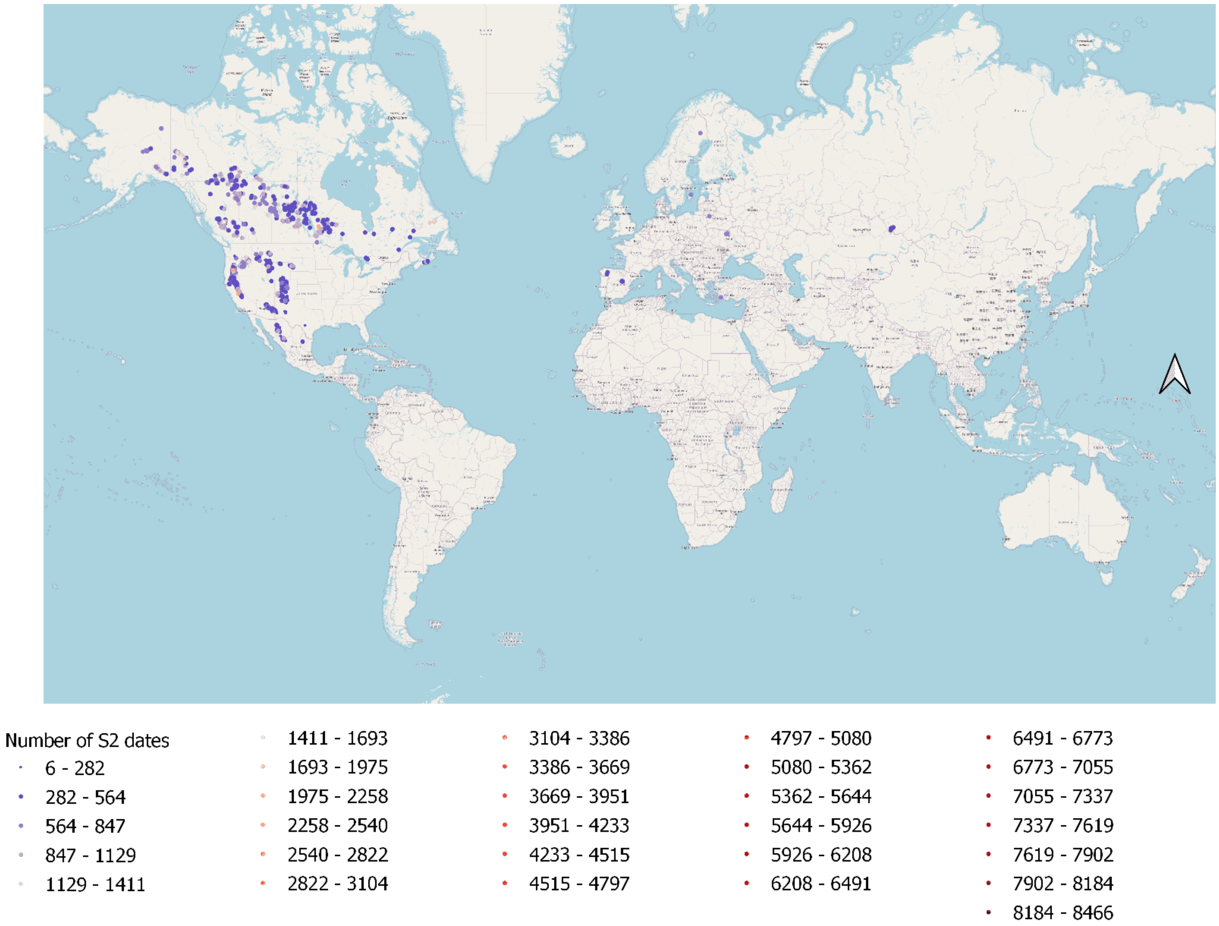

Figure 16. The number of Sentinel-2 images used to build each image in C16 Forest CEN. This number is represented under different intervals.

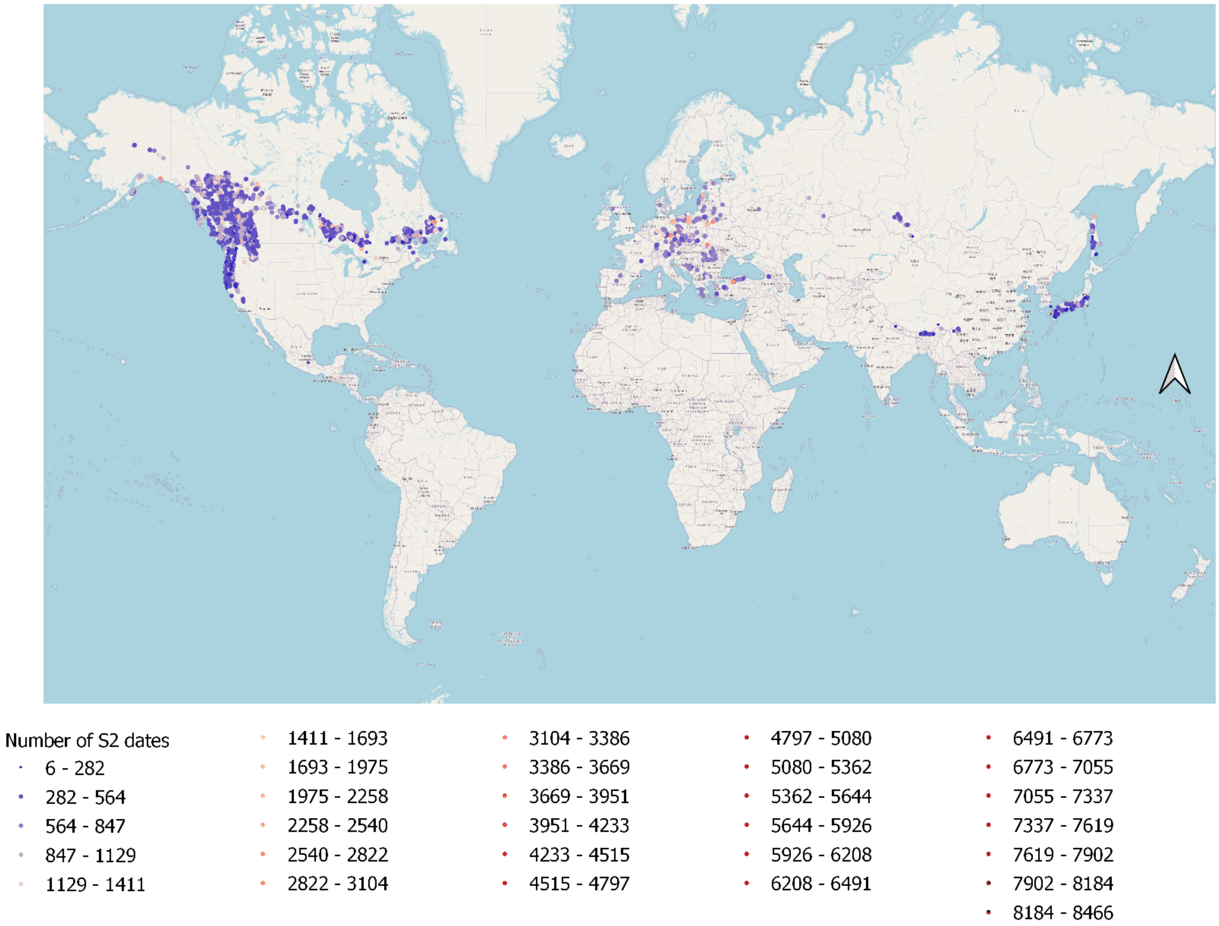

Figure 17. The number of Sentinel-2 images used to build each image in C17 Forest DEN. This number is represented under different intervals.

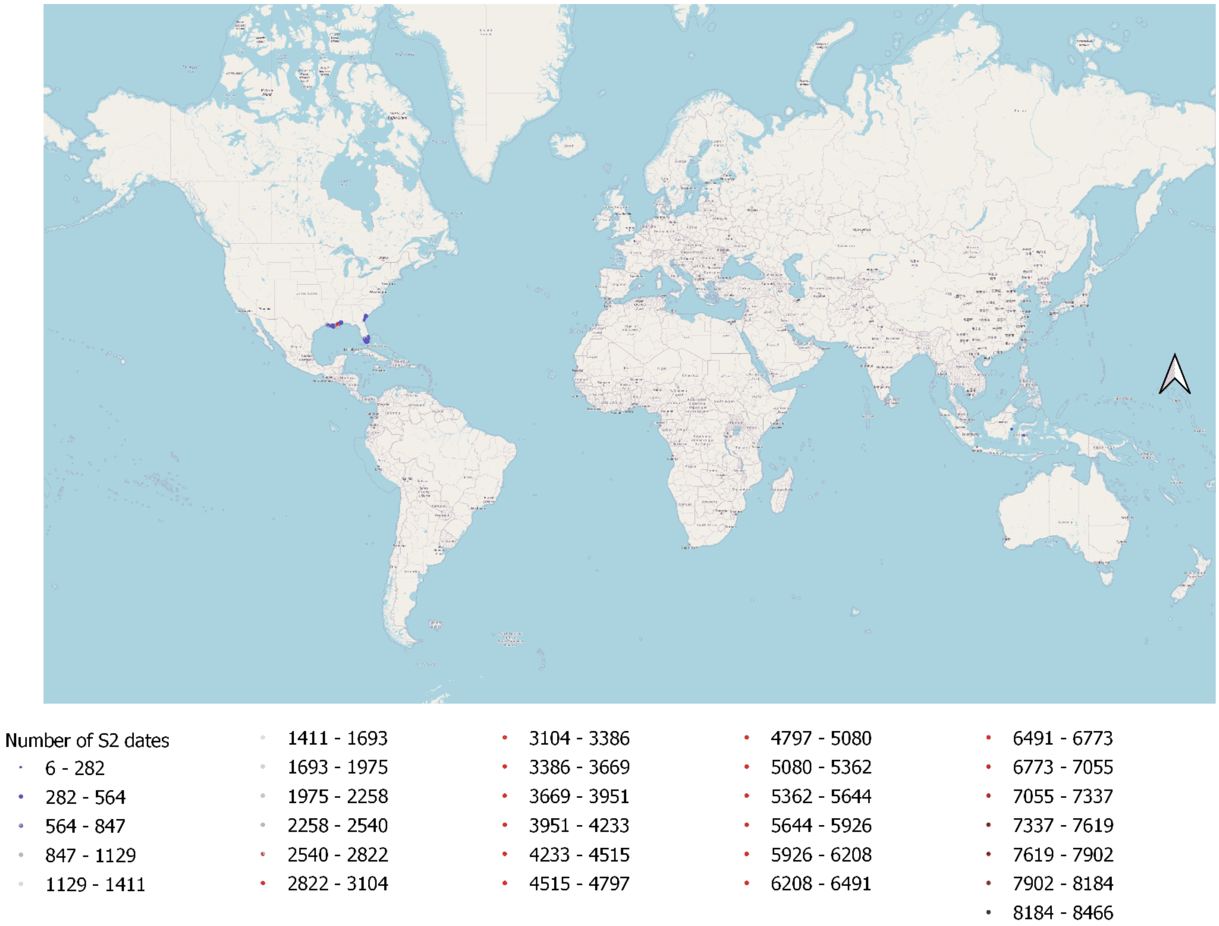

Figure 18. The number of Sentinel-2 images used to build each image in C18 WetlandMangro. This number is represented under different intervals.

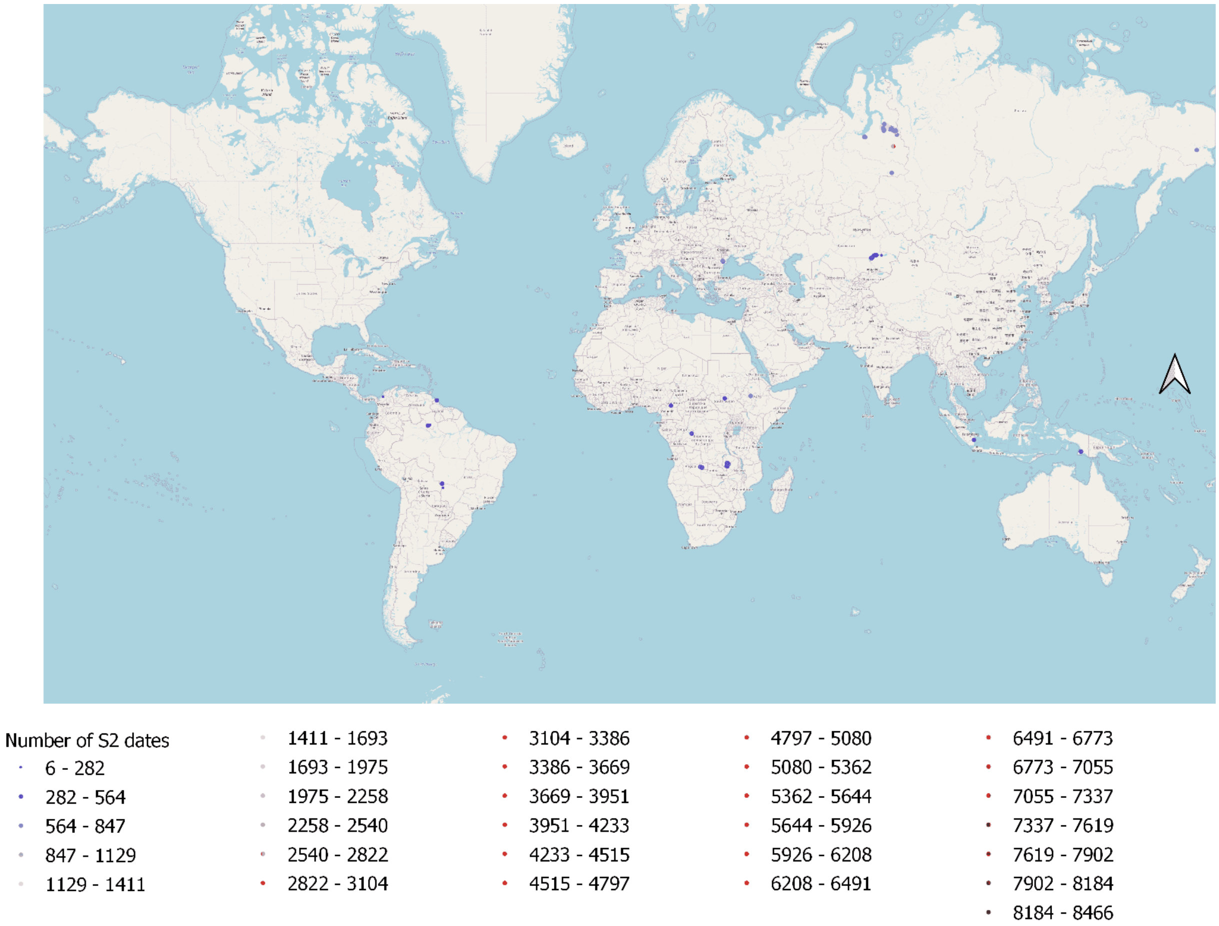

Figure 19. The number of Sentinel-2 images used to build each image in C19 WetlandSwamps. This number is represented under different intervals.

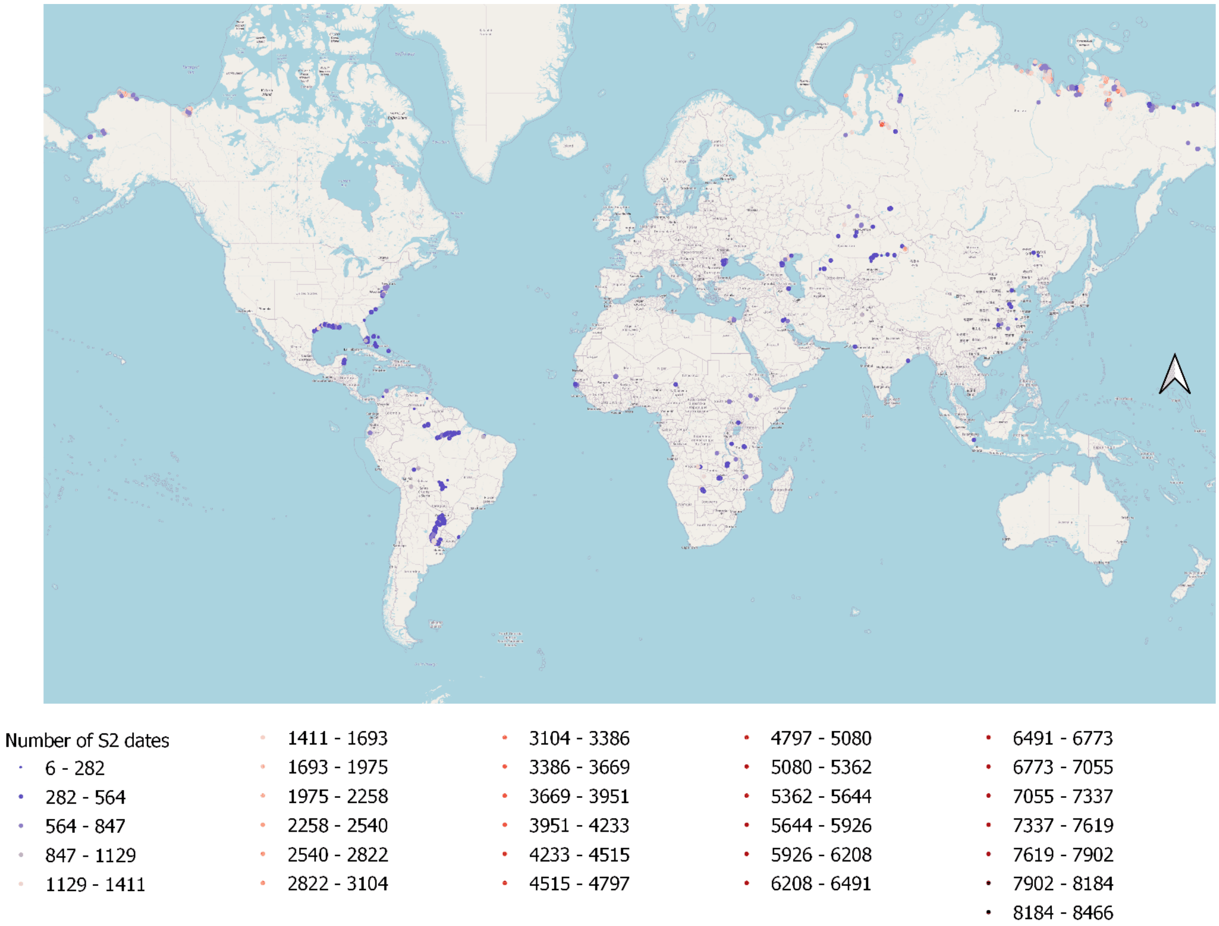

Figure 20. The number of Sentinel-2 images used to build each image in C20 WetlandMarshl. This number is represented under different intervals.

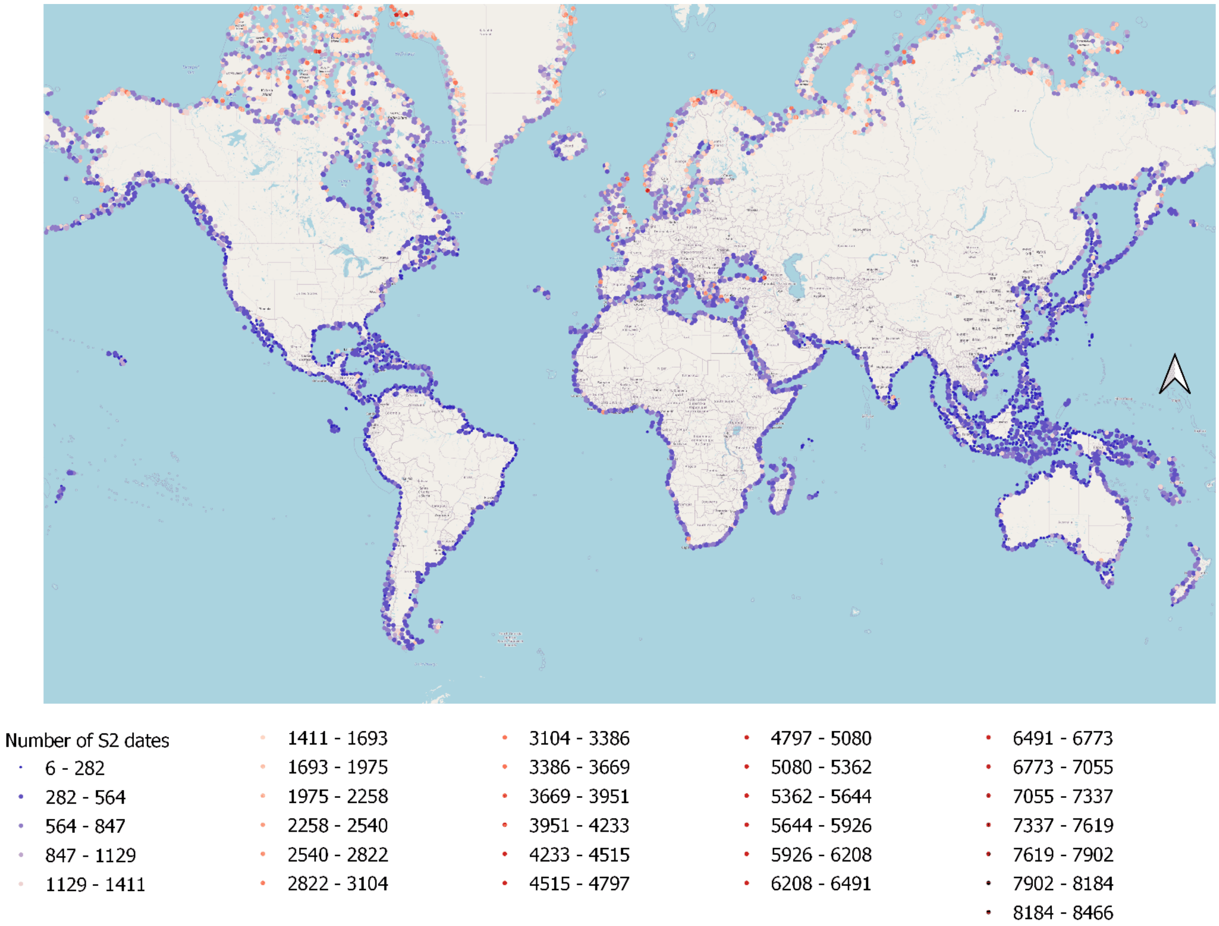

Figure 21. The number of Sentinel-2 images used to build each image in C21 WaterBodyMari. This number is represented under different intervals.

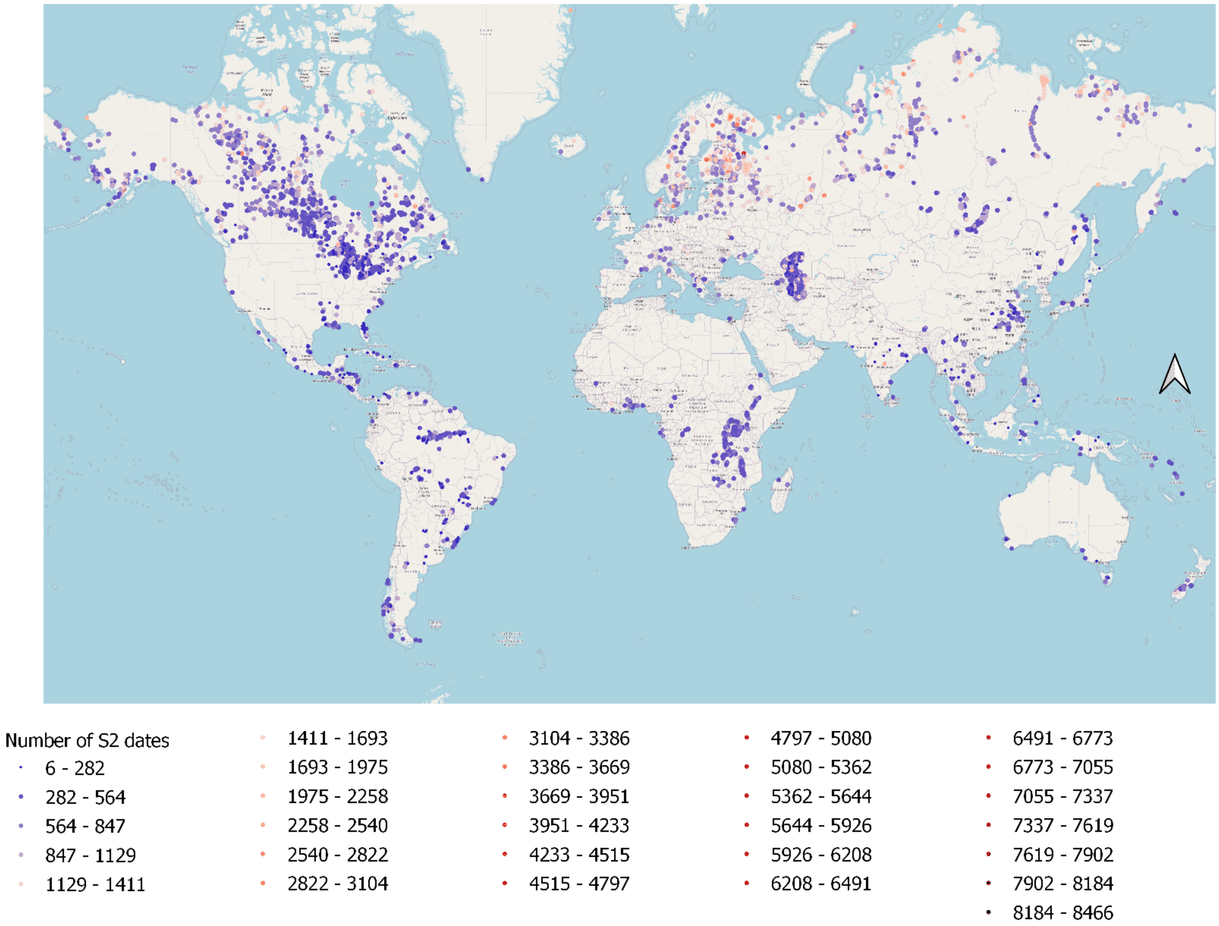

Figure 22. The number of Sentinel-2 images used to build each image in C22 WaterBodyCont. This number is represented under different intervals.

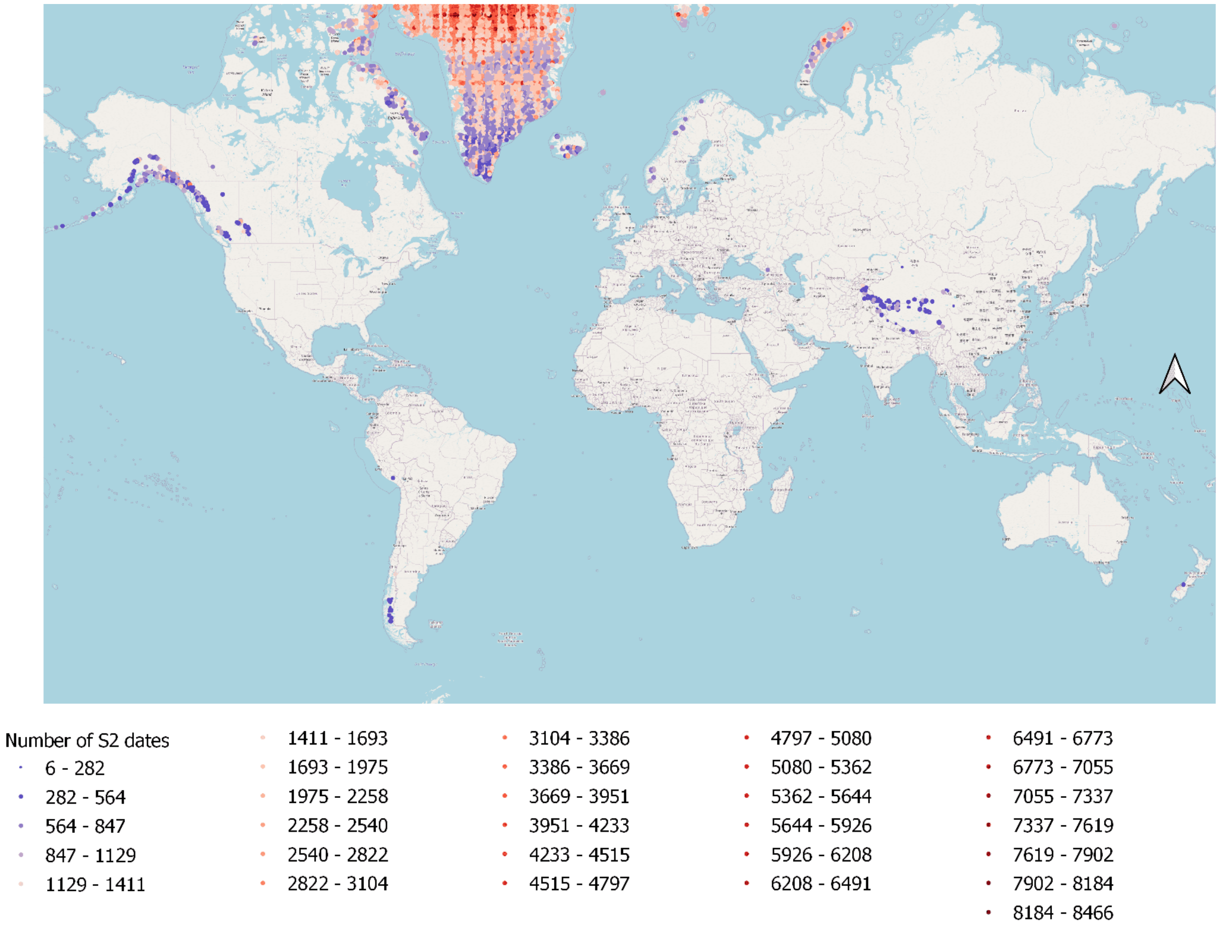

Figure 23. The number of Sentinel-2 images used to build each image in C23 PermanentSnow. This number is represented under different intervals.

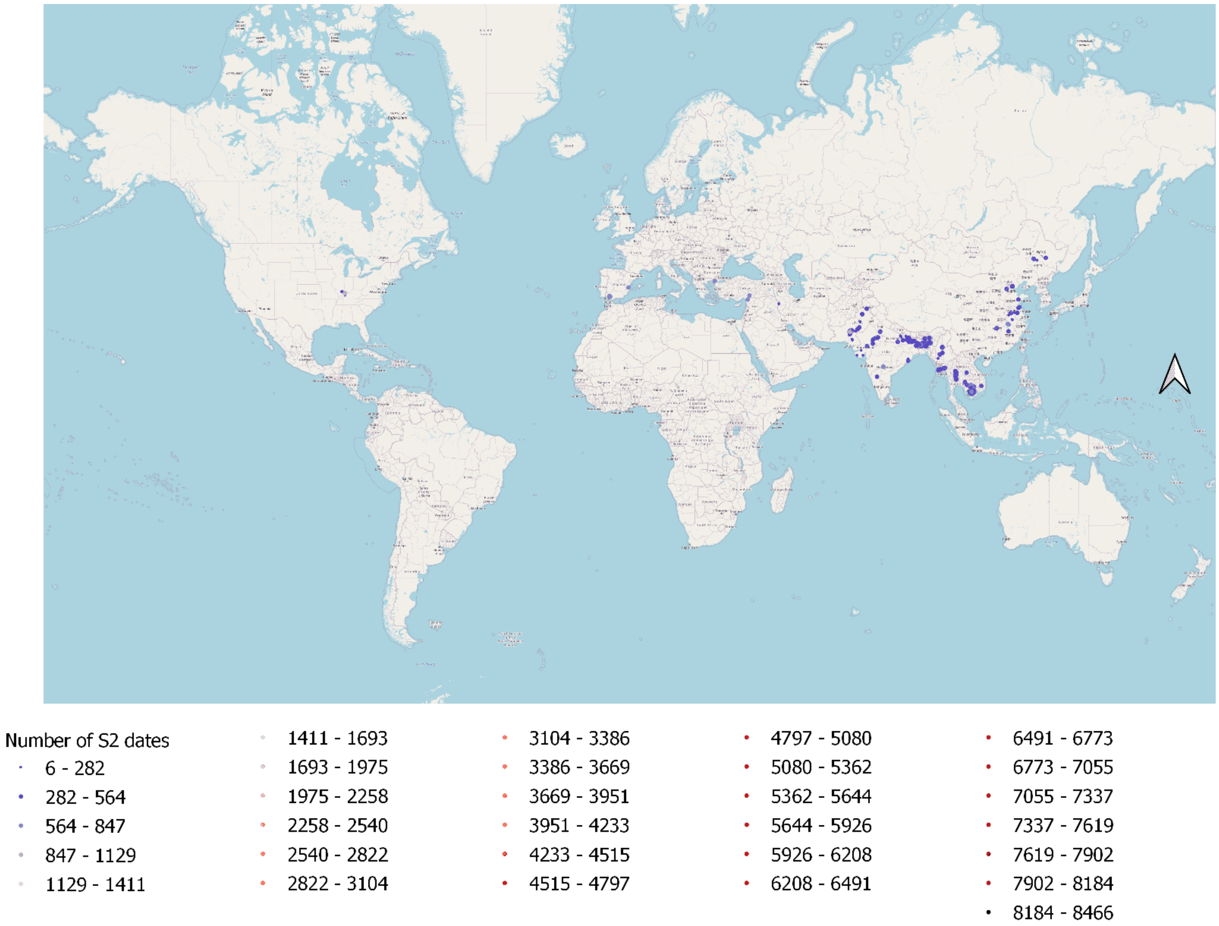

Figure 24. The number of Sentinel-2 images used to build each image in C24 CropSeasWater. This number is represented under different intervals.

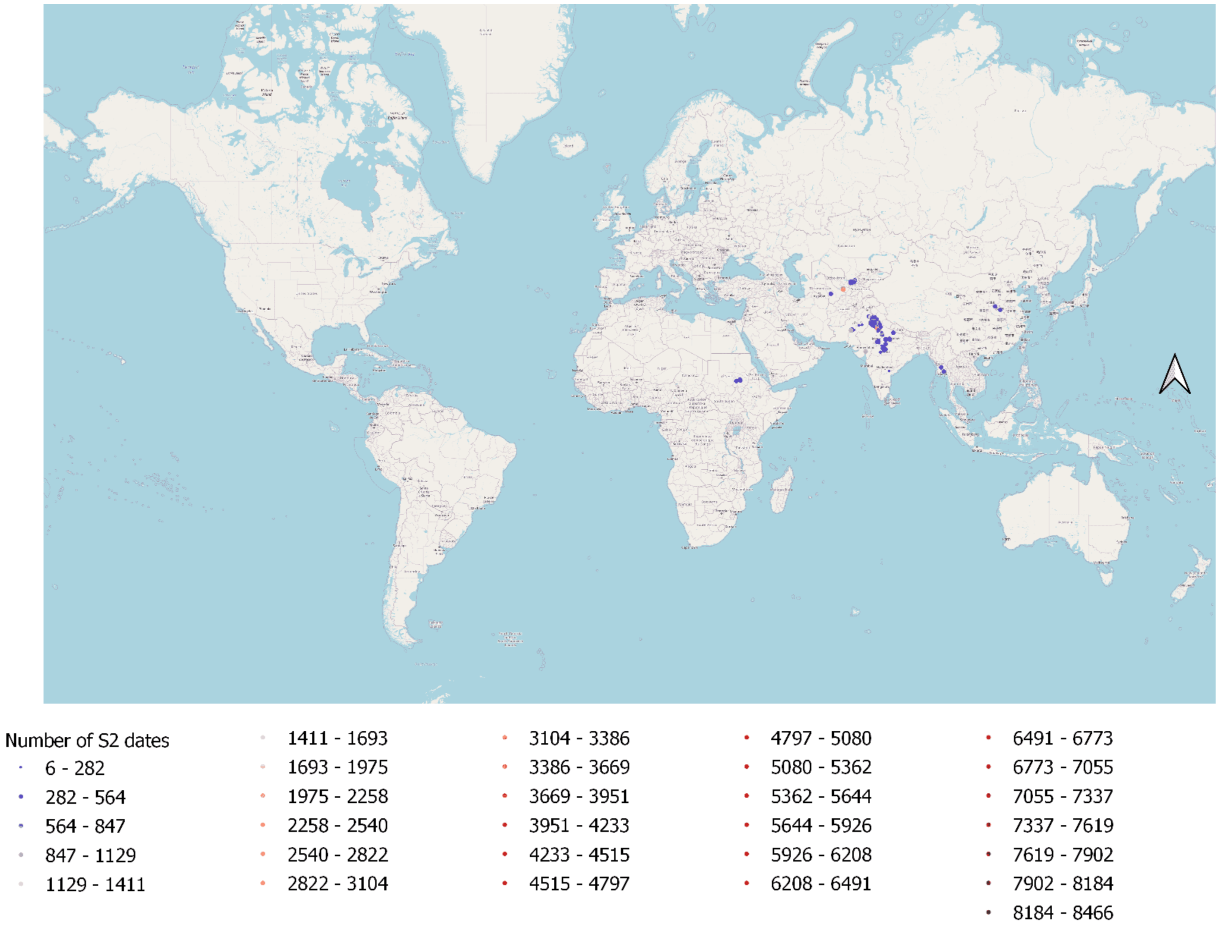

Figure 25. The number of Sentinel-2 images used to build each image in C25 CropCereaIrri. This number is represented under different intervals.

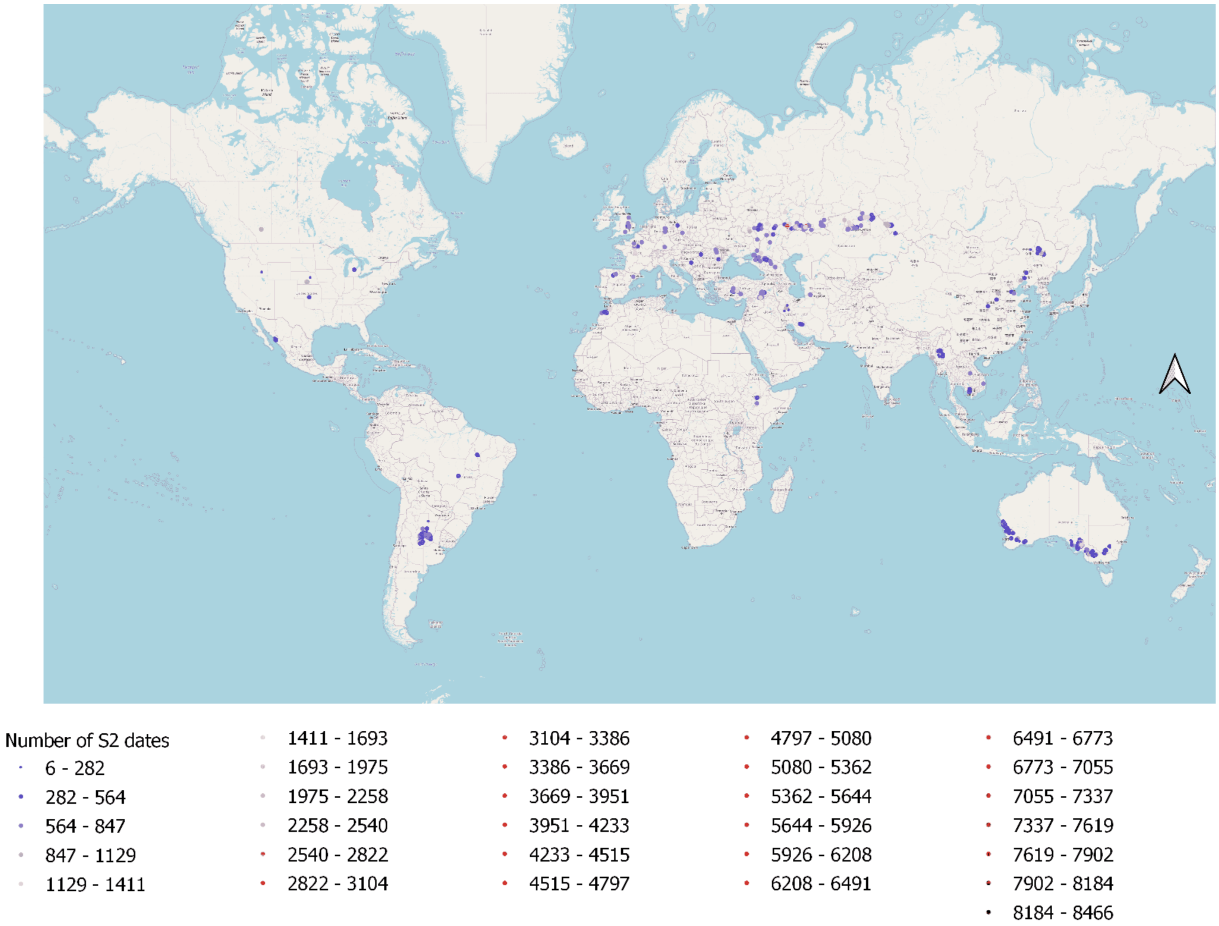

Figure 26. The number of Sentinel-2 images used to build each image in C26 CropCereaRain. This number is represented under different intervals.

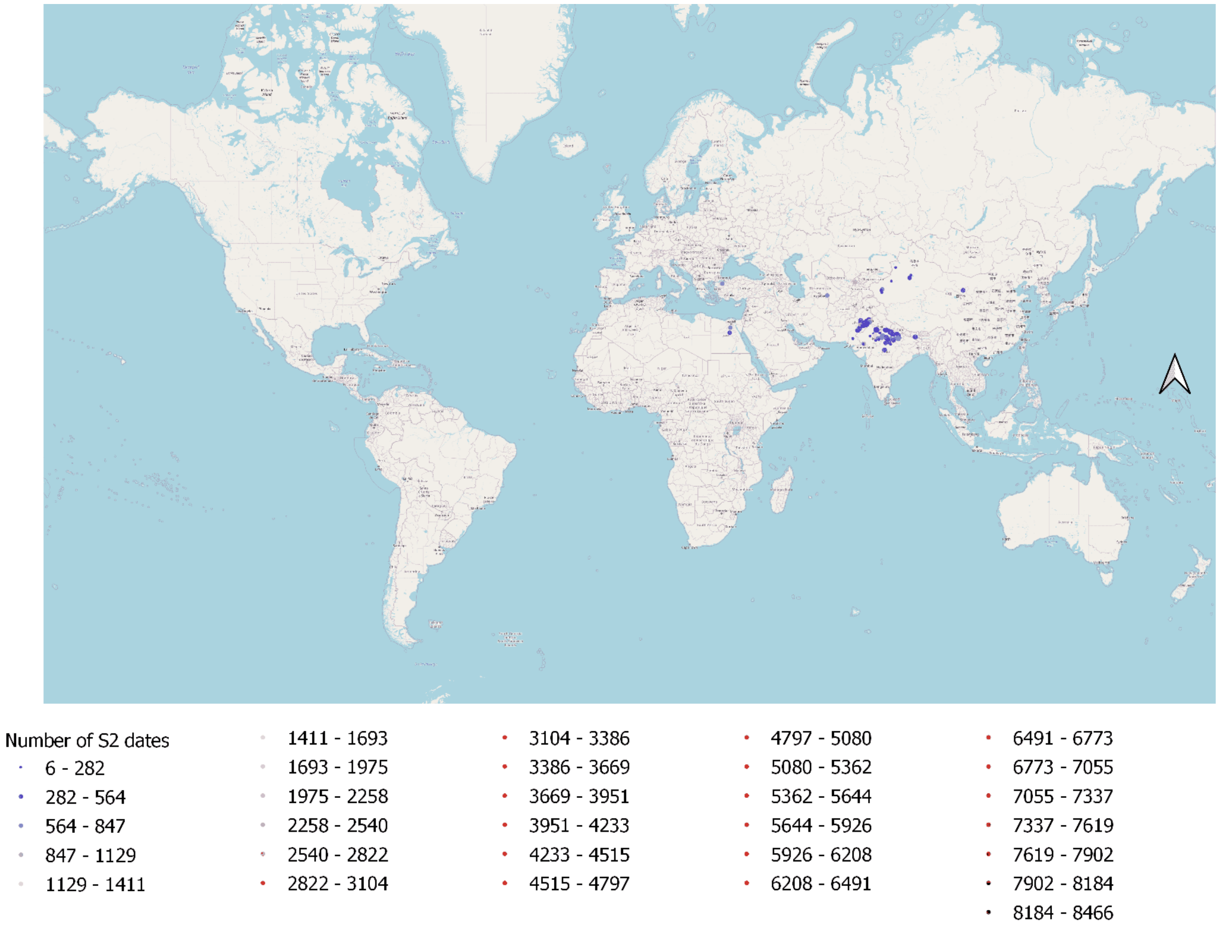

Figure 27. The number of Sentinel-2 images used to build each image in C27 CropBroadIrri. This number is represented under different intervals.

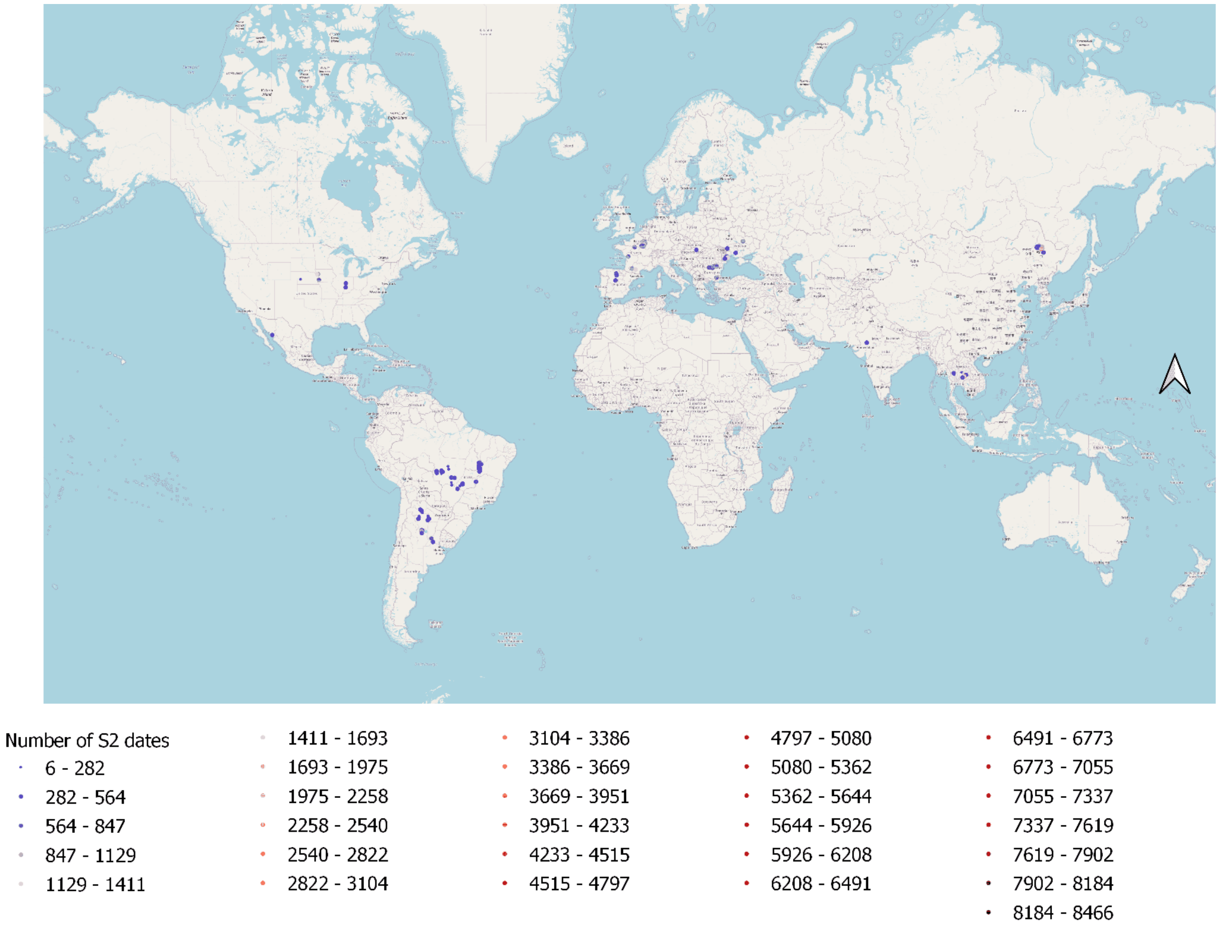

Figure 28. The number of Sentinel-2 images used to build each image in C28 CropBroadRain. This number is represented under different intervals.

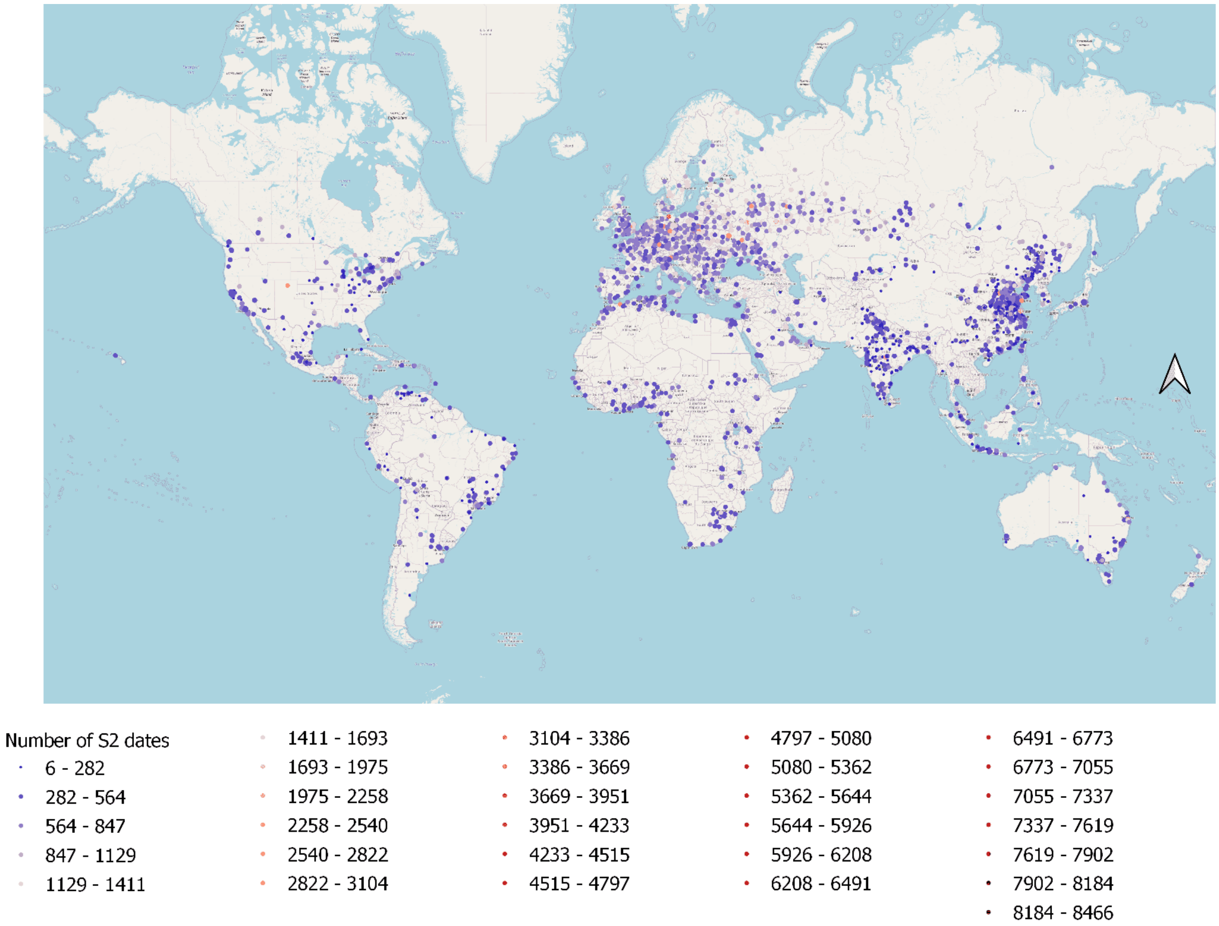

Figure 29. The number of Sentinel-2 images used to build each image in C29 UrbanBlUpArea. This number is represented under different intervals.
